# Inherited *MUTYH* mutations cause elevated somatic mutation rates and distinctive mutational signatures in normal human cells

**DOI:** 10.1038/s41467-022-31341-0

**Published:** 2022-07-08

**Authors:** Philip S. Robinson, Laura E. Thomas, Federico Abascal, Hyunchul Jung, Luke M. R. Harvey, Hannah D. West, Sigurgeir Olafsson, Bernard C. H. Lee, Tim H. H. Coorens, Henry Lee-Six, Laura Butlin, Nicola Lander, Rebekah Truscott, Mathijs A. Sanders, Stefanie V. Lensing, Simon J. A. Buczacki, Rogier ten Hoopen, Nicholas Coleman, Roxanne Brunton-Sim, Simon Rushbrook, Kourosh Saeb-Parsy, Fiona Lalloo, Peter J. Campbell, Iñigo Martincorena, Julian R. Sampson, Michael R. Stratton

**Affiliations:** 1 Cancer, Ageing and Somatic Mutation (CASM), Wellcome Sanger Institute, Hinxton, CB10 1SA UK; 2 Department of Paediatrics, University of Cambridge, Cambridge, CB2 0QQ UK; 3 Institute of Life Science, Swansea University, Swansea, SA28PP UK; 4 Institute of Medical Genetics, Division of Cancer and Genetics, Cardiff University School of Medicine, Cardiff, UK; 5 Hereditary Gastrointestinal Cancer Genetic Diagnosis Laboratory, Department of Pathology, The University of Hong Kong, Queen Mary Hospital, Pokfulam, Hong Kong; 6 Department of Haematology, Erasmus University Medical Centre, 3015 CN Rotterdam, The Netherlands; 7 Nuffield Department of Surgical Sciences, Medical Sciences Division, University of Oxford, Oxford, UK; 8 Department of Oncology, University of Cambridge, Cambridge, UK; 9 Department of Pathology, University of Cambridge, Cambridge, UK; 10 Cambridge University Hospitals NHS Foundation Trust, Cambridge, UK; 11 Norfolk and Norwich University Hospital, Norwich, UK; 12 Norwich Medical School, University of East Anglia, Norwich, UK; 13 Department of Surgery, University of Cambridge, Cambridge, UK; 14 Cambridge NIHR Biomedical Research Centre, Cambridge Biomedical Campus, Cambridge, UK; 15 Manchester Centre for Genomic Medicine, Saint Mary’s Hospital, Oxford Road, Manchester, UK

**Keywords:** Cancer genetics, Cancer genomics, Cancer genomics, Colorectal cancer, Intestinal diseases

## Abstract

Cellular DNA damage caused by reactive oxygen species is repaired by the base excision repair (BER) pathway which includes the DNA glycosylase MUTYH. Inherited biallelic *MUTYH* mutations cause predisposition to colorectal adenomas and carcinoma. However, the mechanistic progression from germline *MUTYH* mutations to MUTYH-Associated Polyposis (MAP) is incompletely understood. Here, we sequence normal tissue DNAs from 10 individuals with MAP. Somatic base substitution mutation rates in intestinal epithelial cells were elevated 2 to 4-fold in all individuals, except for one showing a 31-fold increase, and were also increased in other tissues. The increased mutation burdens were of multiple mutational signatures characterised by C > A changes. Different mutation rates and signatures between individuals are likely due to different *MUTYH* mutations or additional inherited mutations in other BER pathway genes. The elevated base substitution rate in normal cells likely accounts for the predisposition to neoplasia in MAP. Despite ubiquitously elevated mutation rates, individuals with MAP do not display overt evidence of premature ageing. Thus, accumulation of somatic mutations may not be sufficient to cause the global organismal functional decline of ageing.

## Abstract (web summary)

Inherited mutations in *MUTYH* have been shown to predispose patients to colorectal cancers. Here, the authors show that *MUTYH* mutations lead to an increased somatic base substitution mutation rate in normal intestinal epithelial cells, which is the likely cause for the increased cancer risk.

## Introduction

The genomes of all normal human cells are thought to acquire mutations during the course of life. However, the mutation rates of normal cells and the processes of DNA damage, repair and replication that underlie them are incompletely understood^1–8^. A ubiquitous source of potential mutations is DNA damage caused by reactive oxygen species (ROS) which are formed as by-products of aerobic metabolism^9^. ROS cause a variety of DNA lesions, the most common being 8-oxoguanine (8-OG)^10^. As a consequence of mispairing with adenine during DNA replication, 8-OG can cause G:C > T:A (referred to as C > A for brevity) transversion mutations^11^. Under normal circumstances, 8-OG and its consequences are efficiently mitigated by the Base Excision Repair (BER) pathway effected by DNA glycosylases; oxoguanine DNA glycosylase (OGG1) removes 8-OG^12^ and MutY DNA glycosylase (MUTYH) removes adenines misincorporated opposite 8-OG^13^.

Mutations in *MUTYH* engineered in experimental systems can impair its glycosylase activity, reducing its ability to excise mispaired bases and leading to an increased rate of predominantly C > A mutations^14–18^. *MUTYH* mutations inherited in the germline in humans cause an autosomal recessive syndrome (MUTYH-associated polyposis, MAP) characterised by intestinal adenomatous polyposis and an elevated risk of early onset colorectal and duodenal cancer^19–22^. The age of onset and the burden of intestinal polyps are highly variable between individuals, ranging from 10 s to 100 s leading to a substantially increased incidence of colorectal cancer^23–27^. Risks of other cancer types are also thought to be increased^28^.

Colorectal adenomas and carcinomas from individuals with MAP show a predominance of C > A mutations consistent with the presence of an elevated mutation rate attributed to defective MUTYH function^29–33^. However, whether there is an increased mutation rate in normal cells from individuals with biallelic germline *MUTYH* mutations is unknown. If present in normal cells, understanding the magnitude of the increase in mutation rate, the tissues and cell types in which it occurs, the proportion of cells which show it, the mutational processes responsible and the effects of early neoplastic change would provide insight into the genesis of the elevated cancer risk observed in these individuals.

In this study we perform whole-genome sequencing of normal cells from individuals with MAP. Using whole-genome sequencing we characterise the mutation rates and mutational processes in healthy tissues at a near-single-cell resolution. This study identifies the mutational processes that are associated with neoplastic transformation and are likely to underpin the increased cancer risk observed in this population of high-risk individuals.

## Results

### Clinical information

Ten individuals aged 16 to 79 years with biallelic germline *MUTYH* mutations were studied. These included five missense mutation homozygotes (four MUTYH^Y179C+/+^, one MUTYH^G286E+/+^), three compound heterozygotes for the same pair of missense mutations (MUTYH^Y179C+/− G396D+/−^), and two siblings homozygous for a nonsense mutation (MUTYH^Y104*+/+^). These *MUTYH* germline mutations have all been previously recognised as predisposing to MAP^22,23^. All 10 individuals had colorectal polyposis, with between 16 and >100 colonic adenomas, six were known to have duodenal polyps, five had colorectal cancer and one developed jejunal and pancreatic neuroendocrine cancer (Supplementary Data 1).

### Mutation rates in normal intestinal stem cells

An intestinal crypt is constituted predominantly of a population of epithelial cells arising from a single recent common ancestor^34–36^. The somatic mutations which have accumulated over the course of the individual’s lifetime in the ancestral crypt stem cell are present in all its descendant cells^3^. Thus, by sequencing individual crypts, somatic mutations present in the ancestral stem cell can be identified. Using laser-capture microdissection, 144 individual normal intestinal crypts (large intestine *n* = 107 and small intestine *n* = 37) were isolated from the 10 individuals with germline *MUTYH* mutations (Supplementary Data 2). DNA libraries were prepared from individual crypts using a bespoke low-input DNA library preparation method^37^ and were whole-genome sequenced at a mean 28-fold coverage.

**Fig. 1:**
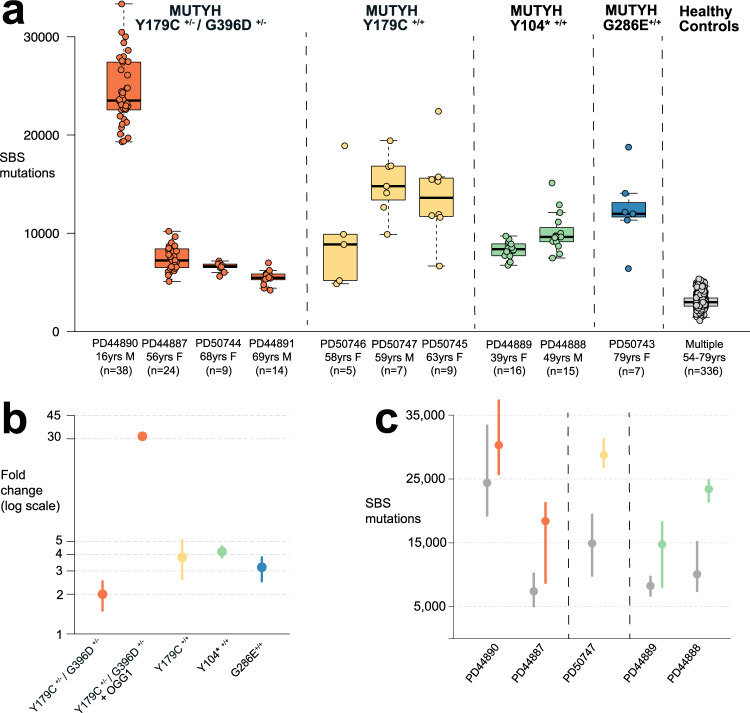
Somatic mutation burdens in cells with *MUTYH* mutations. Elevated mutation burdens in normal intestinal cells with *MUTYH* mutations. **a** Genome-wide single base substitution (SBS) mutation burden of individual intestinal crypts (*n* = 144 biologically independent samples) grouped according to patient. Each dot represents an individual intestinal crypt. *MUTYH* genotypes are displayed separately. Boxplots display median, inter-quartile range (IQR) from 1^st^ to 3^rd^ quartiles and whiskers extend from the last quartile to the last data point that is within 1.5x IQR. **b** Fold-change in SBS rate in intestinal crypts (*n* = 144) with *MUTYH* mutations compared with wild-type controls^3^ (*n* = 445). Fold changes are represented by the dot, whiskers represent the 95% confidence interval (Methods). Dots are coloured according to germline genotype: orange; MUTYH^Y179C+/− G396D+/−^ (*n* = 47), MUTYH^Y179C+/− G396D+/−^ & OGG1 (*n* = 38), yellow; MUTYH^Y179C+/+^ (*n* = 21), green; MUTYH^Y104*+/+^ (*n* = 31), blue; MUTYH^G286E+/+^ (*n* = 7). **c** Genome-wide single base substitution burden in histologically normal crypts (grey) and adenoma crypts (orange, yellow and green) arranged by patient and germline mutation. Data was available for 5 individuals who had adenoma glands sequenced. Dot represents the median and whiskers indicate the range from lowest to highest mutation burden per patient. Source data are provided as a Source Data file.

The single base substitution (SBS) mutation burdens of individual crypts ranged from a median for each individual of 2294 to 33,350, equating to mutation rates of 92-1446 SBS/year, 2-31-fold higher than normal crypts from wild-type individuals (~46 SBS/year) (Fig. 1b, Methods)(linear mixed-effects model 95% confidence interval (C.I.), 69-1520 SBS/yr). Therefore, all normal crypts from all MAP individuals studied showed elevated somatic mutation rates (Fig. 1a, b).

Differences in mutation rate were observed between individuals with MAP (Fig. 1b). A 31-fold higher rate of SBS accumulation than in wild-type crypts^3^ was observed in PD44890, a 16 year old male with MUTYH^Y179C+/− G396D+/−^ who had an aggressive clinical phenotype with a very large number of adenomas and two different primary cancers at an early age. By contrast, the nine other individuals showed only 2- to 4-fold increases in mutation rate compared to wild type. The reason for this substantial difference is not clear. However, in addition to the *MUTYH* mutations, PD44890 carried two heterozygous germline missense variants in *OGG1* (Supplementary Fig. 1a, b), one inherited from the father and the other from the mother (Supplementary Fig. 2). One of these mutations, R46Q, is reported to impair OGG1 activity in experimental systems^38,39^ and has been observed as somatically mutated in human cancer^40^. Germline *OGG1* mutations are not currently recognised as causing cancer predisposition in humans^41^. However, if either or both of these mutations results in defective 8-OG excision they could account for the substantially elevated mutation rate in PD44890, particularly in the context of defective MUTYH activity. The brother of PD44890 shared the same *MUTYH* and *OGG1* germline mutations and demonstrated a similar early onset clinical phenotype, whereas the parents of these siblings were heterozygous for the *OGG1* and *MUTYH* variants and did not show adenomas or cancers.

There was also evidence of differences in mutation rates between the various *MUTYH* germline genotypes studied (Fig. 1b). Excluding the outlier individual PD44890, mutation rates were lower in individuals with the compound heterozygous MUTYH^Y179C+/− G396D+/−^ (93 SBS/year, 95% C.I. 68-116) than individuals with MUTYH^Y179C+/+^ (177 SBS/year, 95% C.I. 121-236), MUTYH^Y104*+/+^ (193 SBS/year, 95% C.I. 173-212) or MUTYH^G286E+/+^ (145 SBS/year, 95% C.I. 117–172) (*P* = 10^−10^, *P* = 10^−7^, *P* = 10^−23^ and *P* = 10^−13^ respectively). The results, therefore, indicate that different *MUTYH* genotypes confer differentially elevated mutation rates and that the extent of the mutation rate increase can be modified by other factors.

SBS mutation rates in coding exons in normal intestinal crypts from MAP individuals were also elevated compared to wild-type individuals (Supplementary Fig. 3a, b). These increases were, however, slightly smaller than those observed in the genome-wide mutation rate (Supplementary Fig. 3a, b). Nonsense, missense and synonymous mutation rates were all increased compared with wild-type crypts, with the greatest increase observed in nonsense mutations (~10-fold more nonsense than wild-type vs ~3.5-fold more missense and ~2.6-fold more synonymous) (Supplementary Fig. 3c). This is attributable to the mutational signatures present (see below) and the tendency of specific mutations at particular trinucleotide contexts to preferentially generate protein-truncating mutations^42,43^.

Neoplastic glands from 13 intestinal adenomas from five individuals with MAP showed SBS mutation burdens that were, on average, ~2-fold higher (range 1.2 to 2.5-fold) (Fig. 1c) than normal crypts from the same individuals sampled at the same time. Therefore, the elevated mutation rate observed in histologically normal intestinal crypts in individuals with germline *MUTYH* mutations is further increased during the process of neoplastic transformation, as previously observed in wild type individuals^44,45^.

Small insertion and deletion (ID) mutations accumulated at a rate of 2.1 ID/yr (linear mixed-effects model, 95% confidence interval (C.I.) 1.2–3.0, *P* = < 10^−4^), which is higher than in wild-type controls (1.3 ID/yr, linear mixed-effects model, C.I. 0.54–2.0, *P* = 0.0011)^3,42^. The cause of this modestly elevated ID rate is not clear. In two MAP individuals additional mutational processes could explain the higher burdens observed in these cases. In PD44890 the high ID mutation rate (ID rate 6/yr) was, at least partially, explained by the presence of an additional ID generating mutational process associated with exposure to the mutagen colibactin produced by a strain of *E*.*Coli*^3,46,47^ present in the colonic microbiome of some people (see below). In PD50747 (ID rate 6/yr), a previously undescribed sporadic ID signature IDA was identified which was not present in other MAP individuals (described below). Structural rearrangements and copy number changes were only observed in a small number of normal intestinal crypts, at similar frequencies to those in wild-type controls (Supplementary Data 2)^3^. Telomere shortening occurred at similar rates in individuals with *MUTYH* mutations compared to wild-type controls (Methods).

### Mutational signatures

**Fig. 2:**
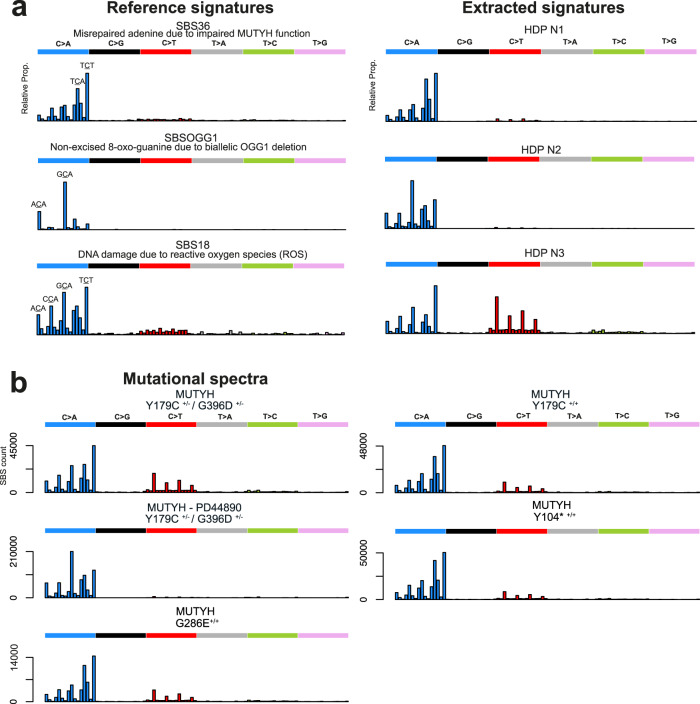
Mutational spectra and signature components from normal cells with MUTYH mutations. **a** Probability distribution for COSMIC reference signature Single Base Substitution (SBS) signature SBS36^40^, recently described *OGG1* deletion signature SBSOGG1^51^ and COSMIC reference signature SBS18^40^. Mutational signature components N1-3 from Hierarchical Dirichlet Process (HDP) de novo signature extraction (see Supplementary Information for all components and Methods for further explanation). **b** Mutational spectra in normal tissues displayed by the germline *MUTYH* mutation. Aggregate mutational spectra of unique somatic mutations from normal crypts with MUTYH^Y179C+/− G396D+/−^ (*n* = 47), MUTYH^Y179C+/− G396D+/−^ - PD44890 (*n* = 38), MUTYH^Y179C+/+^ (*n* = 21), MUTYH^Y104*+/+^ (*n* = 31) and MUTYH^G286E+/+^ (*n* = 7). Distinctive peaks are annotated with their trinucleotide context (mutated base is underlined). PD44890 is displayed separately to highlight the difference in spectrum observed in this individual. Source data are provided as a Source Data file.

Mutational signatures were extracted from the combined catalogues of SBS mutations from all normal and neoplastic intestinal crypts and glands using two independent methods. We then decomposed each de novo extracted signature into known COSMIC reference mutational signatures. Finally, we used these decompositions to estimate the contribution of each reference signature to each sample (Methods, Supplementary Note). Three de novo extracted signatures, N1-N3, accounted for the majority of mutations, all of which were mainly characterised by C > A mutations (Fig. 2a). Two of these (N1 and N3) closely corresponded to the reference mutational signatures SBS18 and SBS36. The third (N2) was abundant only in individual PD44890 (the individual with a high mutation rate carrying *OGG1* germline variants) (Fig. 2b and Supplementary Note) but could also be accounted for by a combination of SBS18 and SBS36 according to the standard parameters used for decomposition (Supplementary Information).

**Fig. 3:**
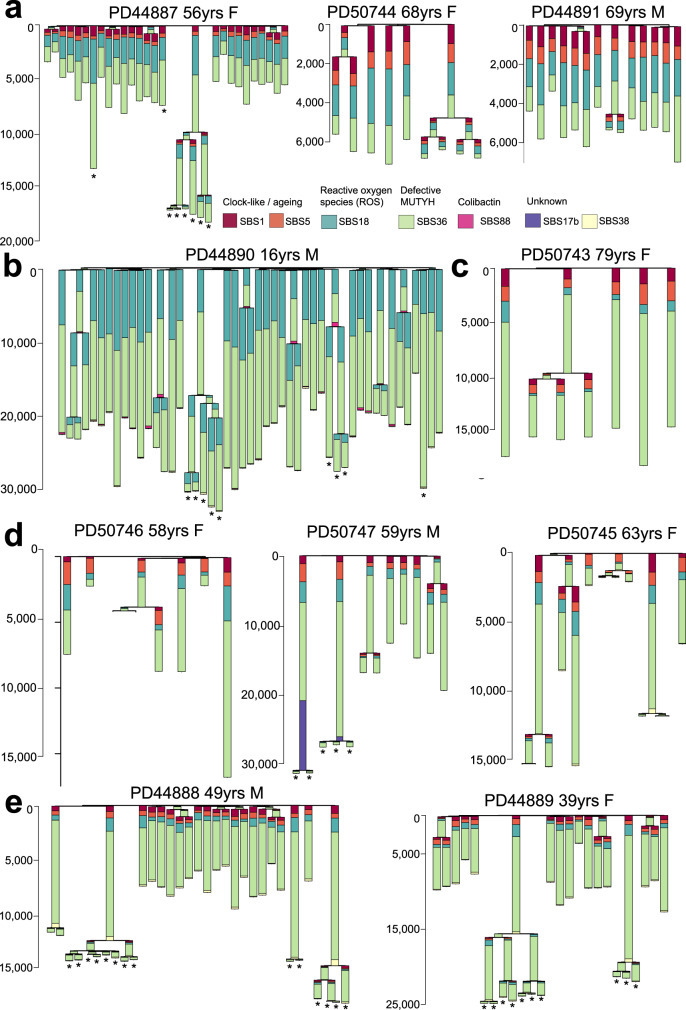
Phylogenetic trees and mutational signatures in intestinal cells with germline *MUTYH* mutations. Phylogenetic trees per-individual reconstructed from SBS mutations in individual intestinal crypts showing the number of SBS mutations per branch. Stacked barplots are overlaid onto each branch to represent the proportion of each mutational signature contributing to that branch. Phylogenetic trees are arranged by *MUTYH* germline mutation; **a** MUTYH^Y179C+/− G396D+/−^ **b** MUTYH^Y179C+/− G396D+/−^ with *OGG1* germline mutations, **c** MUTYH^G286E+/+^, **d** MUTYH^Y179C+/+^ and **e** MUTYH^Y104*+/+^. Adenoma glands bearing cancer driver mutations are indicated with an asterisk ‘*’. Source data are provided as a Source Data file.

Following decomposition and signature attribution, four reference SBS mutational signatures, SBS1, SBS5, SBS18 and SBS36 were identified in all samples (Fig. 3, Supplementary Note). SBS1, due to deamination of 5-methlycytosine at CG dinucleotides and SBS5, of unknown aetiology, have both been found ubiquitously in normal and cancer cells and accumulate in a more or less linear fashion with age^2–4,7,33,48–50^. SBS18, thought to result from DNA damage due to reactive oxygen species, has previously been reported in normal colorectal cells^3^ and many types of cancer^33^ and is characterised by C > A mutations predominantly at ACA, CCA, GCA and TCT trinucleotide contexts (mutated base underlined) (Figs. 2a and Fig. 3). SBS36 has previously been found in cancers with germline or somatic *MUTYH* mutations and is also characterised by C > A mutations, albeit with a different profile of preferred trinucleotide contexts from SBS18^30–33^(Fig. 2a). SBS88, which is predominantly characterised by T > C and T > G mutations, and is due to early life exposure to the mutagenic agent colibactin produced by some strains of *E.Coli*^3,46,47^, was observed in a subset of crypts from PD44890 (16 year old with high mutation rate and *OGG1* mutations, Fig. 3b). The SBS88 mutation burdens were consistent with those previously seen in wild type individuals indicating that MUTYH is unlikely to be implicated in the genesis of SBS88.

The increased SBS mutation burdens in normal crypts from individuals with *MUTYH* germline mutations appeared to be due to the contributions of SBS18 and SBS36 mutations (Fig. 3a–e). The proportions of SBS18 and SBS36, however, differed between *MUTYH* germline genotypes. SBS18 accounted for a substantially higher proportion of mutations in crypts and glands from individuals with the MUTYH^Y179C+/− G396D+/−^ genotype (*n* = 85 crypts) than in individuals with the MUTYH^Y179C+/+^, MUTYH^Y104*+/+^ and MUTYH^G286E+/+^ genotypes (*n* = 59 crypts, Supplementary Fig. 4a). Since MUTYH^Y104*^ causes MUTYH protein truncation, it is conceivable that SBS36 is the consequence of complete loss of MUTYH function and therefore that this is also effected by MUTYH^Y179C^ and MUTYH^G286E^. Conversely, MUTYH^G396D^ may retain partial activity^14,21^ and thus generates a signature more closely resembling SBS18 which is found in normal tissues with fully active MUTYH.

The de novo extracted mutational signature N2, which primarily contributes to the mutational spectra of crypts from PD44890 (MUTYH^Y179C+/− G396D+/−^), resembled reference signature SBS18 (https://cancer.sanger.ac.uk/cosmic/signatures) but showed differences, notably with over representation of C > A mutations at GCA and, to a lesser extent, CCA and ACA trinucleotides (mutated base underlined) (Fig. 2a, b and Supplementary Fig. 1). A signature reported in human cells with in vitro engineered biallelic *OGG1* deletion is also primarily characterised by C > A mutations at GCA and ACA trinucleotides^51^. It is, therefore, possible that mutagenesis due to the germline *OGG1* variant(s) in PD44890 (see above) is superimposed on the mutational signature produced by the *MUTYH* germline mutations to generate N2 (see Supplementary Information for further analysis and discussion).

The mutational signatures in adenoma glands were similar to those seen in normal crypts from the same individuals (Fig. 3a, b, d, e). SBS36 and SBS18 were principally responsible for the increased mutation burdens observed in adenomas compared to normal crypts.

Candidate cancer driver mutations, defined as known or likely oncogenic hotspot mutations and truncating mutations in tumour suppressor genes (Methods, Supplementary Data 3), were observed in 15% of normal crypts (22/144), more than double the rate observed in wild-type crypts from comparable healthy controls; 6% (25/449)^3,42^. A substantial proportion of candidate drivers (16/22) were nonsense mutations, mirroring the broader exome-wide increase in nonsense mutations (Supplementary Fig. 3c), and reflecting the proclivity of certain mutation types to generate truncating mutations^29,42,43^. The mutational spectrum of driver mutations in normal crypts and neoplastic glands resembled the genome-wide spectra with substantial contributions from SBS18 and SBS36 (Supplementary Fig. 5). Hence, the mutational processes resulting from defective MUTYH activity appear to promote the accumulation of putative cancer driver mutations in normal and neoplastic tissues^52,53^.

Three known ID signatures were identified. ID1 and ID2 are characterised predominantly by insertions and deletions of single T bases at T mononucleotide repeats which are associated with strand slippage during DNA replication and are seen in most human cancers and normal tissues^1–4,7,33^. ID18 is associated with colibactin exposure, is found in normal intestinal stem cells and certain cancers, usually associated with SBS88^3,47^. ID1 was the dominant signature in normal cells whereas ID2 predominated in neoplastic cells (Supplementary Fig. 6). ID18 was principally observed in samples from PD44890 (16 years old with the high mutation burden and *OGG1* mutations) and is responsible for the elevated ID rate in this individual (Supplementary Fig. 7). A further ID signature, IDA, identified in PD50747, was characterised by single C insertions at C mononucleotide repeats (Supplementary Figs. 7, 31 and 35). IDA was present in both normal crypts (~5% of total ID burden) and to a greater extent in adenoma glands (~20% of total ID burden). The cause of this previously undescribed signature is unclear but may be associated with previous capecitabine treatment in this individual and seems unlikely to be related to germline *MUTYH* mutations.

### Mutations in other cell types

To investigate whether the elevated mutation rates and mutational signatures observed in intestinal epithelium caused by defective MUTYH are present in other cell types, peripheral blood and tissue lymphocyte DNAs from individuals with biallelic *MUTYH* mutations were whole genome sequenced using a duplex sequencing method (NanoSeq)^50^ that allows mutation calling from single DNA molecules and thus accurately discovers somatic mutations in tissues in which multiple clonal lineages are intimately mixed.

**Fig. 4:**
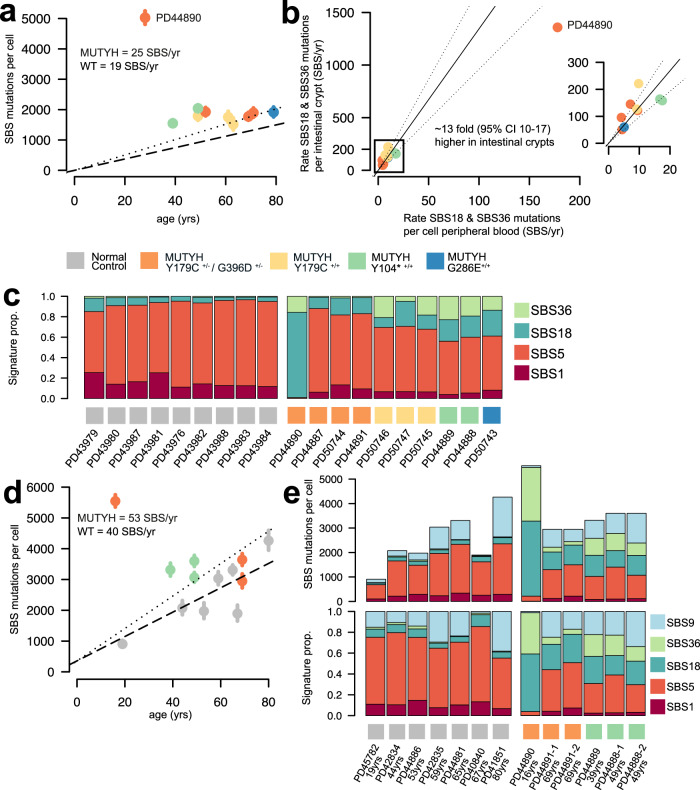
Mutation burdens and mutational signatures in blood and immune cell populations. **a** SBS mutation burden in peripheral blood per cell (*x*-axis) plotted against the age of the individual in years (*y*-axis). Dots are coloured according to the individual’s germline mutation; orange; MUTYH^Y179C+/− G396D+/−^, yellow; MUTYH^Y179C+/+^, green; MUTYH^Y104*+/+^ and blue; MUTYH^G286E+/+^. Whiskers represent the 95% confidence interval. Dashed line represents the mutation rate in wild-type (WT) normal control samples, dotted line represents the mutation rate in *MUTYH* samples (linear mixed-effects-model)^50^. **b** Mutation rate of *MUTYH* associated mutational signatures; SBS18 and SBS36 per cell for peripheral blood (SBS/yr)(x-axis) against the SBS18 & SBS36 mutation rate of normal intestinal crypts (SBS/yr)(y-axis). Each dot represents one individual and they are coloured according to the individual’s germline mutation; orange; MUTYH^Y179C+/− G396D+/−^, yellow; MUTYH^Y179C+/+^, green; MUTYH^Y104*+/+^ and blue; MUTYH^G286E+/+^. The rate of *MUTYH* associated mutational processes is ~13x fold (linear model, 95% C.I.; 10-17) higher in intestine vs blood. Black line indicates the ratio, and dotted lines the 95% C.I.. Plot inset shows the mutation rate for *n* = 9 patients excluding the outlier, PD44890. **c** Stacked bar plot displaying the mutational signature contribution in each peripheral blood sample organised by patient. Coloured squares indicate the *MUTYH* germline mutation. Normal control data from granulocytes sequenced using the same method (data from Abascal et al 2021)^50^. Significantly higher proportion of SBS18 and SBS36 is observed in individuals with *MUTYH* mutations vs normal healthy controls (two-sided Wilcoxon rank sum, *P* = 0.00004). **d** SBS mutation burden in intestinal lymphocyte cells from wild-type healthy individuals (grey) and individuals with *MUTYH* mutations (coloured according to the germline *MUTYH* genotype) plotted against age (years). Dots represent median values and whiskers represent the 95% confidence interval. Dashed line indicates the rate of increase of SBS burden in wild-type lymphocytes (40 SBS/yr, linear mixed-effects model, *R*^2^ = 0.68, 95% C.I., 13-66, *P* = 0.01) and dotted line indicates the rate of increase in SBS burden in lymphocytes from individuals with *MUTYH* mutations (53 SBS/yr, linear mixed-effects model, *R*^2^ = 0.68, 95% C.I., 21–85, *P* = 0.01). **e** Stacked bar plots showing the absolute (above) and relative (below) contributions of each mutational signature in tissue lymphocytes from wild-type healthy individuals and individuals with *MUTYH* mutations. Source data are provided as a Source Data file.

The blood cell SBS mutation rates of all individuals with *MUTYH* mutations were higher than wild-type controls (*n* = 15 granulocyte samples from 9 healthy individuals aged 20-80 yrs)(Fig. 4)(25 SBS/yr vs 19 SBS/yr, linear mixed-effects model, *R*^2^ = 0.89, *MUTYH*; 95% C.I., 19-31, *P* = 10^−7^ and wild-type; 95% C.I., 14-24, *P* = 10^−6^). The relative increases in blood mutation rates were lower than in intestinal crypts from each individual (Fig. 4b). Nevertheless, the relative increases paralleled the differential increases observed between individuals in intestinal crypts. SBS mutation rates in tissue lymphocytes were modestly raised compared with wild-type healthy individuals (Fig. 4d) (53 SBS/yr vs 40 SBS/yr, linear mixed-effects model, *R*^*2*^ = 0.68, *MUTYH*; 95% C.I., 21-85, *P* = 0.01 and wild-type; 95% C.I., 13-66, *P* = 0.01). The signatures associated with defective MUTYH, SBS18 and SBS36, contributed the excess mutations in all samples (Fig. 4c, e). An additional mutational signature was seen in lymphocytes. SBS9, which is associated with DNA polymerase eta mediated somatic hypermutation and is a key process in the physiological maturation of B-cells, was observed in most lymphocyte samples indicating that the lymphocyte cell populations contained mature B-cells (Fig. 4e).

## Discussion

This study shows elevated base substitution somatic mutation rates due to SBS18 and/or SBS36 in normal tissues from individuals with *MUTYH* mutations. The results are compatible with all intestinal, and potentially all other cells in the body, showing elevated mutation rates. The relative increases in mutation rate and mutational signature composition differed between individuals, probably due to different *MUTYH* mutations and perhaps to other modifying influences.

We have previously highlighted the capability of normal human cells to tolerate substantially elevated mutation rates^42^. Carriers of *POLE* and *POLD1* exonuclease domain germline mutations exhibited elevated somatic mutation burdens without evident cellular or organismal consequences, other than an increased cancer risk^42,54^. This capability is confirmed in *MUTYH* germline mutation carriers. It is further emphasised by the observation of a 31-fold genome-wide elevated base substitution mutation burden in the 16 year old PD44890, which would confer a “mutational age” of ~500 years, without overt evidence of premature ageing. The increase in mutation burden in coding exons is lower than genome-wide in *POLE*/*POLD1* mutation carriers. Similarly, in individuals with *MUTYH* mutations there is a smaller increase of coding exon than genome-wide mutation burdens (Supplementary Fig. 3a, b). Nevertheless, in PD44890 the increase is still ~29-fold, and therefore equivalent to a “mutational age” of ~450 years. Whilst lesser increases in mutation rates compared to wild-type individuals were observed in other tissues from PD44890, ~8-fold in white blood cells and ~7 fold in tissue lymphocytes, these still conferred substantially elevated “mutational ages” in the absence of features of premature ageing. Thus, direct deleterious effects of base substitutions accumulated over the course of a lifetime may not be an important cause of ageing.

The elevated mutation rate in normal intestinal epithelium likely contributes to the increased risk of colorectal adenomas and cancers in individuals with *MUTYH* mutations. Indeed, there appears to be a correlation between the extent of elevation of mutation rate and the rate of acquisition of colorectal adenomas. Individuals with the MUTYH^Y104*+/+^ and MUTYH^Y179C+/+^ genotypes exhibited greater increases in somatic mutation rates than individuals with the MUTYH^Y179C+/− G396D+/−^ genotype. Previous detailed clinical phenotyping of large series indicates that individuals with biallelic truncating mutations or MUTYH^Y179C+/+^ show higher rates of accumulation of adenomas and earlier age of onset of carcinoma^22,23^ than MUTYH^Y179C+/− G396D+/−^. The correlation between elevation of mutation rate and severity of clinical phenotype is further highlighted by individual PD44890 (16 years of age, MUTYH^Y179C+/− G396D+/−^) who exhibited a substantially higher mutation rate than others of this genotype, and showed a much accelerated rate of colorectal adenoma development (Supplementary Data 1, 2). We previously described ~7-fold elevated genome-wide base substitution mutation rates in intestinal cells of *POLE* germline exonuclease domain mutation carriers^42^. *POLE* mutation carriers, however, show lower colorectal adenoma rates than *MUTYH* biallelic mutation carriers who generally only show 2-4 fold increased mutation rates. This apparent discrepancy may, however, be explained by the genomic distribution of mutations. In *POLE* mutation carriers there is relative sparing of coding sequences, with only a three to four-fold increase in exonic mutations in intestinal cells, whereas this sparing is less pronounced in *MUTYH* mutation carriers leading to similar increases in exonic mutation rates (Supplementary Fig. 8a, b). These observations lead to the proposition that measurements of somatic coding mutation rates undertaken early in life could, in future, be used to refine individual cancer risk predictions for *POLE/POLD* and *MUTYH* germline mutation carriers.

As for many other cancer predisposition syndromes, it is unclear why *MUTYH* germline mutations lead particularly to intestinal neoplasia. Elevated somatic mutation rates are also found in white blood cells in MAP individuals (and may therefore be present in other tissues) although the increases appear lesser in extent than in intestinal cells. The propensity to generate SBS18 mutations appears greater in wild type intestinal cells than in other cell types^49^ and this may also be contributory. Extending this study to investigate somatic mutagenesis in a greater number of tissues with varying cancer incidence and in larger cohorts of individuals may offer further insight into the role mutation rates and mutational signatures play in tissue specific cancer risk.

In summary, we report elevated somatic base substitution rates characterised by distinctive mutational signatures in normal tissues from individuals with MAP. These findings underscore previous observations that elevated somatic base substitution rates are largely tolerated by cells and do not overtly accelerate the process of ageing. It is likely, however, that increased mutation rates in normal intestinal cells throughout life lead to increased rates of accumulation of driver mutations and, hence, the procession of neoplastic clones culminating in cancer.

## Methods

### Ethical approval and study participants

This research complies with all relevant ethical regulations. MAP patients were recruited as part of Wales Research Ethics Committee (REC) 12-WA0071 and 15-WA0075 and samples collected were approved for use in this project by REC 18/ES/0133. Normal healthy controls were recruited as part of the following UK Research Ethics Committee (REC) studies; 15/WA/0131, 15/EE/0152, 18/ES/0133 and 08/h0304/85 + 5.

Informed consent was obtained from all participants and no monetary compensation was offered for their participation. Consent was obtained for publication of demographics including age, sex, phenotypic features and other potentially identifiable data. A complete list of study participants and tissue samples is summarised in Supplementary Data 1, 2.

### DNA extraction from bulk samples

Frozen whole blood underwent DNA extraction using the Gentra Puregene Blood Kit (Qiagen). Briefly, 1–2 ml of frozen blood were thawed, lysed in RBC lysis solution and centrifuged. Cell pellet was resuspended in cell lysis solution and incubated at 37 °C for 2 h. RNA and protein were degraded using RNase A solution and protein precipitation solution. DNA was precipitated with isopropanol.

### Tissue preparation

Tissues were embedded in Optimal Cutting Temperature (OCT) compound, frozen histological sections were cut at 25–30 µm and mounted on polyethylene naphthalate (PEN) slides and fixed in 70% ethanol for 5 minutes followed by two washes with phosphate buffered saline for 1 min each. Slides were manually stained in haematoxylin and eosin using a conventional staining protocol. A subset of samples were fixed in RNAlater (Sigma Aldrich) according to manufacturer’s instructions. Fixed tissue samples were embedded in paraffin using a Tissue-Tek tissue processing machine (Sakura). No formalin was used in the preparation, storage, fixation or processing of samples. Processed tissue blocks were embedded in paraffin wax, sectioned to 10 µm thickness and mounted onto PEN slides (Leica). Tissue slides were stained using a standard haematoxylin and eosin (H&E) protocol. Slides were temporarily cover-slipped and scanned on a NanoZoomer S60 Slide Scanner (Hamamatsu), images were viewed with NDP.View2 software (Hamamatsu).

### Histopathological review of tissues

All tissues studied were carefully reviewed using the following approach: (1) tissue blocks were reviewed by a pathologist / clinician at the time of sampling and classified based on their macroscopic appearance as being normal or adenoma. (2) Following histological sectioning and high-resolution scanning, tissue sections were categorised as being normal, adenoma or cancer. Slides that were indeterminate were referred to a gastrointestinal pathologist for review. (3) Prior to laser-capture microdissection, each crypt was carefully inspected using the 40x digital scan and classified as being normal or dysplastic / adenoma. Initial review, in stages 2 and 3, was principally undertaken by an experienced clinician with a special interest in gastrointestinal pathology. Glands were classified as adenomatous if they bore histological features indicating them to be dysplastic or likely dysplastic and harboured a cancer “driver” mutation.

### Laser capture microdissection

Laser capture microdissection was undertaken using a LMD7000 microscope (Leica) into a skirted 96-well PCR plate. Cell lysis was undertaken using 20 µl proteinase-K PicoPureⓇ DNA Extraction kit (ArcturusⓇ). Samples were incubated at 65 °C for 3 h followed by proteinase denaturation at 75 °C for 30 min. Thereafter samples were stored at −20 °C prior to DNA library preparation.

### Low-input DNA library preparation and sequencing

DNA library preparation of micro-dissected tissue samples was undertaken using a bespoke low-input enzymatic-fragmentation-based library preparation method^2–4,37^. This method was employed as it allows for high quality DNA library preparation from a very low starting quantity of material (from 100-500 cells). In brief, gDNA was purified from cell lysates using bead purification. Enzymatic fragmentation, end-repair and dA-tailing was performed using NEBNext Ultra II FS DNA Library Prep Kit (New England BioLabs). Indexing and PCR amplification was subsequently performed (12 cycles). DNA library concentration was assessed after library preparation and used to guide choice of samples to take forward to DNA sequencing. The minimum library concentration was 5 ng/µL and libraries with >15 ng/µL were preferentially chosen. 150 bp paired-end Illumina reads were prepared with Unique Dual Index barcodes (Illumina). DNA sequencing was undertaken on a NovaSeq 6000 platform using an XP kit (Illumina). Samples were multiplexed in pools of 6-24 samples. Pools were sequenced to achieve a coverage of ~30x per sample.

### Mutation calling and post-processing filters

Sequencing reads were aligned to NCBI human genome GRCh37 using the Burrow-Wheeler Alignment (BWA-MEM). Single Base Substitutions (SBS) were called using the ‘Cancer Variants through Expectation Maximization’ algorithm (CaVEMan)^55^. Mutations were called using an unmatched normal synthetic bam file to retain early embryonic and somatic mutations. Post-processing filters were applied to remove low-input library preparation specific artefacts and germline mutations using a previously described method^1,2,37,56^. Filters applied were: (1) common single nucleotide polymorphisms were removed by filtering against a panel of 75 unmatched normal samples^57^ (2) to remove mapping artefacts, mutations were required to have a minimum median read alignment score of mutant reads (ASMD ≥ 140) and fewer than half of the reads supporting the mutation should be clipped (CLPM = 0) (3) a filter to remove overlapping reads that result from the relatively short insert size, which could lead to double counting of variant reads; and (4) a filter to remove cruciform DNA structures that can arise during the low-input library preparation method.

Next, we applied multiple filters to remove germline variants and potential artefacts whilst retaining bona fide embryonic and somatic variants. This approach has been detailed in previous publications and the code for these filters can be found at https://github.com/TimCoorens/Unmatched_NormSeq. Mutations were aggregated per patient and a read pile-up was performed using an in-house algorithm (cgpVAF) to tabulate the read count of mutant and reference reads per sample for each mutation locus. Germline mutations were filtered out using an exact binomial test which distinguishes germline from somatic variants using the aggregate read counts across all samples from the same patient^1,56^. In brief, the read depth across all samples from each individual was calculated (median in this study 496-fold). This high coverage yields a very precise estimate of the true VAF of each mutation. While the VAF estimates of the earliest embryonic SBS and germline variants from samples sequenced at 30x might overlap, the VAFs from the much higher coverage achieved by aggregating all samples from each individual, are distinguishable using statistical testing. To achieve this, the beta-binomial test was applied. The over dispersion parameter (rho) threshold for genuine variants of rho > 0.1 was used.

Phylogenetic trees were created using MPBoot (version 1.1.0 bootstrapped – 1000 times) and mutations were mapped to branches using maximum likelihood assignment.

Indels (ID) were called using Pindel^58^ using the same synthetic unmatched normal sample employed in SBS mutation calling. ID calls were filtered to remove calls with a quality score of <300 (‘Qual’; sum of mapping qualities of the supporting reads) and a read depth of less than 15. Thereafter, ID filtering was performed in a similar manner as SBS to remove germline variants and library preparation / sequencing artefacts.

### Copy-number alteration calling

Somatic copy-number variants (CNVs) were called using the Allele‐Specific Copy number Analysis of Tumours (ASCAT) algorithm^59^ as part of the in the ascatNGS package^60^ (https://github.com/Crick-CancerGenomics/ascat). Bulk blood samples or phylogenetically unrelated normal samples were used as matched normals. ASCAT was run with default parameters. A bespoke filtering algorithm - ascatPCA - was used to reduce the number of false-positive calls that can arise when analysing genome sequences from normal tissue (https://github.com/hj6-sanger/ascatPCA). ascatPCA extracts a noise profile by aggregating the LogR ratio from across a panel of normal unrelated samples and subtracts this signature from that observed in the sample being analysed using principal component analysis.

### Structural variant calling

Whole-genome sequences were analysed for somatic structural variants (SVs) using the Genomic Rearrangement Identification Software Suite (GRIDSS). In preparation for this analysis, genomes were remapped to Human Genome Version 38 and GRIDSS was run using the same matched normal as used for CNV analysis. Coordinates for SV calls were subsequently converted back to GRCh37. SV calls in L1 transposon donor regions and fragile sites were excluded from the final SV analysis.

### Mutational signature analysis

The R package, HDP (https://github.com/nicolaroberts/hdp), based on the hierarchical Dirichlet process^61^, was used to extract mutational signatures. Analysis of mutational signatures using this package has been applied to normal tissues previously^1–4^. In brief, this nonparametric Bayesian method models categorical count data using the hierarchical Dirichlet process. A hierarchical structure is established using patients as the first tier (parent nodes) and individual samples as the second tier (dependent nodes). Uniform Dirichlet priors were applied across all samples. The algorithm creates a mutation catalogue for each sample and infers the distribution of signatures in any one sample using a Gibbs sampler. We performed mutational signatures analysis per-branch, counting each branch of the phylogenetic tree as a distinct sample to avoid double counting of mutations. Since the MCMC process scales linearly with the number of counts, we randomly subsampled each branch to a maximum of 2500 total substitutions. Branches with fewer than 100 mutations were excluded from the mutational signature extraction. No reference signatures were included as priors.

Next, to estimate the contribution of each mutational process, mutational signatures were refitted to all mutation counts using the R package sigfit (https://github.com/kgori/sigfit)^62^. To avoid overfitting, a limited subset of reference mutational signatures were included for each patient corresponding to the HDP signatures that were identified in that individual.

Ageing signatures SBS1 and SBS5 are present in all normal intestinal crypts^3^. Lower than expected burdens of SBS1 and SBS5 were observed in most individuals in this study due to: (1) the inherent challenges of accurately estimating mutation burden in highly mutated samples and (2) the appreciable contamination of reference signatures with SBS1 and SBS5. To partially address this, we used the extracted HDP component corresponding to SBS36 in the refitting stage which has lower SBS1 and SBS5 contamination than the COSMIC reference SBS36 signature. Nevertheless, in individual PD44890 where SBS18 and SBS36 exposures are many tens of times greater than the normal mutation rate, the estimates of SBS1 and SBS5 are substantially lower than would be expected.

Settings / parameters used for mutational signature extraction:

SBS Signature Extraction - Hierarchical Dirichlet Process (HDP)

Chains: 20 MCMC chains

Iterations: 40,000

Burn-in: 20,000

Samples: 100 / chain

Signature components identified: 9

Component Names: HDP N0-HDP N8

SBS Signature Extraction – Sigprofiler

input_type: vcf

startProcess: 1

endProcess: 15

totalIterations: 1000

cpu: −1

hierarchy: True

refgen: GRCh37

genome_build: GRCh37

mtype: [‘default’]

init: random

### Mutational signature extraction

Signature extraction was performed using two independent methods; the Hierarchical Dirichlet Process (HDP) and SigProfiler. De novo extraction was performed to extract / identify mutational signatures. HDP signature extraction yielded 9 signature components (N0-N8) (Supplementary Figs. 9–17). Components showing close similarity to known reference signatures and were retained as their reference signature (HDP N0 as SBS5, HDP N1 as SBS36, HDP N5 as SBS5, HDP N6 as SBS38, HDP N8 as SBS17b). To deconvolute the other signature components and equate them to known COSMIC reference signatures, an expectation maximisation algorithm was used. Decomposed signature components are shown in Supplementary Figs. 18–21. HDP N2 was broken down into SBS18 and SBS36, HDP N3 into SBS1, SBS18 and SBS36 and HDP N7 into SBS18 and SBS88. A further component, HDP N4 was unable to be fully deconvoluted into known mutational signatures so was retained in its original format for the next stage of analysis.

Signatures were re-fitted to the mutation counts for each branch of the phylogenetic tree to establish the absolute contributions of each mutational signature using the R package SigFit (https://github.com/kgori/sigfit). To prevent overfitting, a limited subset of reference signatures was used corresponding to HDP components identified in that patient. Furthermore, any signatures occurring at less than 10% exposure were excluded to prevent over-fitting. Therefore 7 mutational signatures were refitted; SBS1, SBS5, SBS17b, SBS18, SBS36, SBS38 and SBS88.

### Validation of mutational signatures

To validate the mutational signatures extracted using the HDP method, we used the non-negative matrix factorisation (NNMF) based algorithm SigProfiler. Using the same input data, SigProfiler generated 5 signature components (Sigprofiler A-E, Supplementary Fig. 23). SigProfiler generated fewer signature components than HDP (5 vs 9). SigProfiler components SigProfiler.A, SigProfiler.B, SigProfiler.C which accounted for the majority of mutations in the data set, had clear counterparts among the HDP signature components (HDP N1-3) (Supplementary Figs. 22–26). Additional signature components were stably extracted by HDP but not by SigProfiler.

### ID mutational signature analysis

ID mutational signatures were extracted using the HDP method identifying a total of 5 signatures (HDP N0-N4 (Supplementary Figs. 27–31). Component HDP N1 bore close similarity to COSMIC reference signature ID1, component HDP N2 to ID1 and ID2; and HDP N3 to ID18 (Supplementary Fig. 32–34). Component HDP N4 had no clear comparator among known reference signatures, and deconvolution was unable to adequately recapitulate the original signature component.

ID mutational signature extraction was validated using SigProfiler, which identified 4 signature components that closely correspond to those identified by HDP (Supplementary Fig. 35).

### Cancer driver mutations

Cancer driver mutations were identified using two methods aiming to identify genes and mutations in this cohort that are subject to positive selection. Firstly, to identify mutations in cancer genes under positive selection in an unbiased manner, we ran a modified dNdS method^63^. To avoid double-counting of mutations, only unique mutations (SBS and ID) mapped to branches of the phylogenetic trees were analysed. dNdScv was run using the following parameters; max_coding_muts_per_sample = 5000 and max_muts_per_gene_per_sample = 20. Genes with a qval of <0.05 were considered to be under positive selection.

A second phase of cancer driver mutation analysis was undertaken, identifying mutations in this cohort that are codified in cancer mutation databases and exhibit characteristic traits of driver mutations, an approach that has been previously employed in the study of normal tissues^1,2^. Analysis was restricted to somatic mutations (SBS and ID) in protein coding regions and mutations were filtered using lists of known cancer genes; mutations in samples from intestinal epithelium were filtered using a list of 90 genes associated with colorectal cancer which includes genes that are common in small bowel adenocarcinoma^3^. Samples from all other tissues, including blood, were filtered using a pan-cancer list of 369 driver genes^63^. Genes were then characterised according to their predominant molecular behaviour: dominant, recessive or intermediate (those demonstrating aspects of both types of behaviour), using the COSMIC Cancer Gene Census^64^. Potential hotspot mutations were annotated using the cBioportal MutationMapper database (https://www.cbioportal.org/mutation_mapper). Mutations meeting the following criteria were considered to be driver mutations: truncating mutations (those that cause a shortened RNA transcript i.e. nonsense, essential splice-site, splice region and frameshift ID) in recessively acting genes, known activating hotspot mutations in dominant (and recessive) genes. Lastly, mutations that were in neither of the above categories but were characterised by the MutationMapper database as being ‘likely oncogenic’ were also included in the final driver mutation catalogue. We then compared the frequency of driver mutations in histologically normal crypts with *MUTYH* mutations to a cohort of *n* = 445 normal intestinal crypts^3^ from wild-type individuals that were analysed using the same method.

### Generation and processing of data from the wild-type control cohort

Data generated in this study was compared to a cohort of healthy individuals with no germline *MUTYH* mutation. The wild-type cohort was generated as part of a previously published study^3^ and comprises *n* = 445 normal intestinal crypts that were processed using the same laboratory methods employed in this paper. While most of the bioinformatic analysis in the original paper followed the same pipeline employed in this study, there were some small differences in the methods used for filtering. Therefore, we re-filtered the mutations in the wild-type cohort using the same parameters employed in the *MUTYH* cohort. Mutation burden estimates were corrected for sensitivity in the same manner as the *MUTYH* cohort (described below). This was particularly important as the wild-type cohort was sequenced at a lower median coverage than the *MUTYH* cohort (~16-fold vs ~28-fold). In the original study, Lee-Six et al report a mutation burden of 43.6 SBS/yr in normal crypts. Here, using data that was re-filtered, we obtain an estimate of the mutation rate that is highly concordant (46 SBS/yr).

### Mutation calling sensitivity

The sensitivity of calling mutations in a genome sequence is strongly influenced by the depth of sequencing coverage and clonality of the sample. Natural variation in sequencing coverage and the clonality of samples may, therefore, influence the sensitivity to call mutations and hence the genome-wide mutation burden estimate. To account for these differences, we calculated the sensitivity of mutation calling from its two principal determinants, sequencing coverage and clonality using a previously validated method^3,37,47^. First, we sampled a range of sequence coverage values from a Poisson distribution centred around the mean coverage for the sample being analysed. Next, using these values we simulated the number of sequencing reads at each site using a truncated binomial distribution based on the median VAF of each sample. The sensitivity was then calculated as the fraction of simulated mutation calls with 4 or more reads, which is the minimum number of reads that the SBS mutation calling algorithm, CaVEMan, requires to call a mutation. The genome-wide mutation SBS burden was then corrected by dividing it by the estimated sensitivity to give the corrected SBS mutation count.

### Mutation burden estimates, modelling and fold-changes

Statistical modelling was performed to assess the mutation rate associated with each germline genotype. A linear mixed-effects model was used to assess the SBS mutation rate for each of the main *MUTYH* germline genotypes and the re-filtered wild-type control data. Fold-changes in the SBS mutation rate were calculated by dividing the modelled mutation rates for the five different *MUTYH* germline mutation groupings by the modelled mutation rate for the wild-type group.

### Telomere content estimation

Telomere attrition is a hallmark of ageing observed in normal cells^65,66^. To assess whether telomere shortening is altered in normal tissues from individuals with MAP compared with wild-type controls, we used a bioinformatic method to assess the telomere content of DNA sequencing files called TelomereHunter. TelomereHunter has previously been applied to the study of telomere biology in cancer samples and normal tissues^42,67,68^. We applied TelomereHunter to estimate the telomere content in normal intestinal crypts with *MUTYH* mutations (*n* = 144) and in wild-type controls (*n* = 445). Next, we used linear mixed-effects modelling to assess the rate of telomere attrition in the *MUTYH* and wild-type cohorts. No significant difference was observed, thus implying that telomere maintenance is not overtly dysregulated in individuals with germline *MUTYH* mutations.

### Modified duplex sequencing (NanoSeq)

DNA from bulk blood samples from individuals with germline *MUTYH* mutations was extracted as outlined above. Samples from normal healthy control was obtained and processed using the following method. Whole blood was diluted with PBS and mononuclear cells (MNC) were isolated using lymphoprepTM (STEMCELL Technologies) density gradient centrifugation. The red blood cell and granulocyte fraction of the blood was then removed. The MNC fraction was depleted of red blood cells by lysis steps involving 3 incubations at room temperature for 20 mins/10 mins/10 mins respectively with RBC lysis buffer (BioLegend). Tissue lymphocytes were isolated from Peyer’s patches in intestinal mucosa using laser capture microdissection and subjected to protein lysis as outlined above. Cell lysates were processed and whole genome sequenced using the NanoSeq protocol.

Our modified duplex sequencing method, called NanoSeq, relies on blunt-end restriction enzymes to fragment the genome in order to avoid errors associated to the filling of 5′ overhangs and the extension of internal nicks during end repair after sonication. Our modified method has error rates < 5e-9^50^.

Given the uneven frequencies of trinucleotides in the digested genome, the strong filtering of common SNPs sites (typically occurring at CpG), and the strong dependence of mutation rates on trinucleotide contexts, our estimates of mutation burdens are normalised and projected onto genomic trinucleotide frequencies.

Let *t* denote the count of a given trinucleotide of type *i* = 1…32. The frequency of each trinucleotide is calculated separately for the genome ${f}_{i}^{g}$and for the NanoSeq experiment ${f}_{i}^{e}$ where (Formula 1):

1 $${f}_{i}=\frac{{t}_{i}}{{\sum }_{i=1}^{32}{t}_{i}}$$

The ratio of genomic to experimental frequencies for a given trinucleotide is (Formula 2):

2 $${r}_{i}=\frac{{f}_{i}^{g}}{{f}_{i}^{e}}$$

There are *j* = 1…6 classes of substitution where the mutated base is a pyrimidine. Let *s*_*ij*_ denote the count of substitution *j* in trinucleotide context *i*, giving a total of 96 substitution classes. Each substitution count is corrected as follows (Formula 3):

3 $${s}_{{ij}}^{{\prime} }={s}_{{ij}}{r}_{i}$$

The corrected substitution counts provide a substitution profile projected onto the human genome, and are also used to calculate the corrected mutation burden (Formula 4):

4 $${\beta }^{{\prime} }=\frac{{\sum }_{i=1}^{32}{\sum }_{j=1}^{6}{s}_{{ij}}^{{\prime} }}{{\sum }_{i=1}^{32}{t}_{i}}$$

Software used in this study is publicly available at the following locations:

- Mutation calling algorithms are available at https://github.com/cancerit.
- Code for filtering mutation calls is available at https://github.com/TimCoorens/Unmatched_NormSeq.
- Software for mutational signature analysis is available at https://github.com/nicolaroberts/hdp and https://github.com/kgori/sigfit and https://github.com/AlexandrovLab.
- Software for analysis of duplex / NanoSeq data is provided at https://github.com/cancerit/NanoSeq.
- Parameters used for these various pieces of software have been included in the manuscript methods section and supplementary information.

### Reporting summary

Further information on research design is available in the Nature Research Reporting Summary linked to this article.

## Supplementary information

Supplementary Information

Supplementary Data Table 1

Supplementary Data Table 2

Supplementary Data Table 3

Reporting Summary

## Data Availability

Source data are provided with this paper. Raw DNA sequencing data are deposited in the European Genome-Phenome Archive (EGA) with accession codes: EGAD00001007958 and EGAD00001007997. To ensure the data is used for academic and research purposes, the DNA sequencing data are available via controlled access. Applications to access the data should be directed to the WTSI CGP Data access committee via the contact details listed at the above links. Indefinite access to the data will be made upon request. Further details of the access policy are available at https://ega-archive.org/submission. The cBioPortal MutationMapper database was accessed at: https://www.cbioportal.org/mutation_mapper?standaloneMutationMapperGeneTab=ATM. The COSMIC Cancer Gene Census is available to download at: https://cancer.sanger.ac.uk/census. There are no restrictions to accessing the MutationMapper or COSMIC databases.

## Source data

Source Data

## Code availability

Code/software required to reproduce the analyses in this paper are available online and are listed in Methods. Code required to reproduce the figures in this manuscript are available online^69^ at: https://github.com/PhilipSRobinson/mutyh; 10.5281/zenodo.6504797.
