# Supplementary material for: Inherited MUTYH mutations cause elevated somatic mutation rates and distinctive mutational signatures in normal human cells
Source: Nat Commun. 2022 Jul 8;13:3949. doi: 10.1038/s41467-022-31341-0 (PMC9270427; doi:10.1038/s41467-022-31341-0)
Supplement: Supplementary file 1 — Supplementary Information [file 41467_2022_31341_MOESM1_ESM.pdf]

Inherited *MUTYH* mutations cause elevated somatic mutation rates and distinctive mutational signatures in normal human cells

P Robinson et al.

29/04/2022

Contents

Supplementary Figures 3
Supplementary Figure 1 3
Supplementary Figure 2 5
Supplementary Figure 3 6
Supplementary Figure 4 8
Supplementary Figure 5 9
Supplementary Figure 6 10
Supplementary Figure 7 12
Supplementary Figure 8 14
Supplementary Note 1 - Mutational Signature Analysis 15
HDP SBS Signature components 15
Supplementary Figure 9 - SBS HDP N0 16
Supplementary Figure 10 - SBS HDP N1 17
Supplementary Figure 11 - SBS HDP N2 18
Supplementary Figure 12 - SBS HDP N3 19
Supplementary Figure 13 - SBS HDP N4 20
Supplementary Figure 14 - SBS HDP N5 21
Supplementary Figure 15 - SBS HDP N6 22
Supplementary Figure 16 - SBS HDP N7 23
Supplementary Figure 17 - SBS HDP N8 24
HDP SBS Signature components - deconvolution 25
Supplementary Figure 18 - SBS HDP N2 26
Supplementary Figure 19 - SBS HDP N3 27
Supplementary Figure 20 - SBS HDP N4 28
Supplementary Figure 21 - SBS HDP N7 29
Comparison of HDP and Sigprofiler *de novo* signature extraction 30

Supplementary Figure 22 - HDP Components . . . 31
Supplementary Figure 23 - SigProfiler Components . . . 36
Supplementary Figure 24 - HDP SBS signature exposure . . . 37
Supplementary Figure 25 - SigProfiler SBS signature exposure . . . 37
Supplementary Figure 26 - HDP vs SigProfiler cosine similarity matrix . . . 38
HDP ID Signature components . . . 39
Supplementary Figure 27 - ID HDP N0 . . . 40
Supplementary Figure 28 - ID HDP N1 . . . 41
Supplementary Figure 29 - ID HDP N2 . . . 42
Supplementary Figure 30 - ID HDP N3 . . . 43
Supplementary Figure 31 - ID HDP N4 . . . 44
HDP ID Signature components - deconvolution . . . 45
Supplementary Figure 32 - ID HDP N0 . . . 46
Supplementary Figure 33 - ID HDP N2 . . . 47
Supplementary Figure 34 - ID HDP N4 . . . 48
SigProfiler ID Signature components . . . 49
Supplementary Figure 35 . . . 50
Supplementary Data - Legends . . . 51
Supplementary Data 1 . . . 51
Supplementary Data 2 . . . 51
Supplementary Data 3 . . . 51
Supplementary Note 2 - Mutational signatures in PD44890 . . . 52
Supplementary Table 1 . . . 52

Supplementary Figures

Supplementary Figure 1

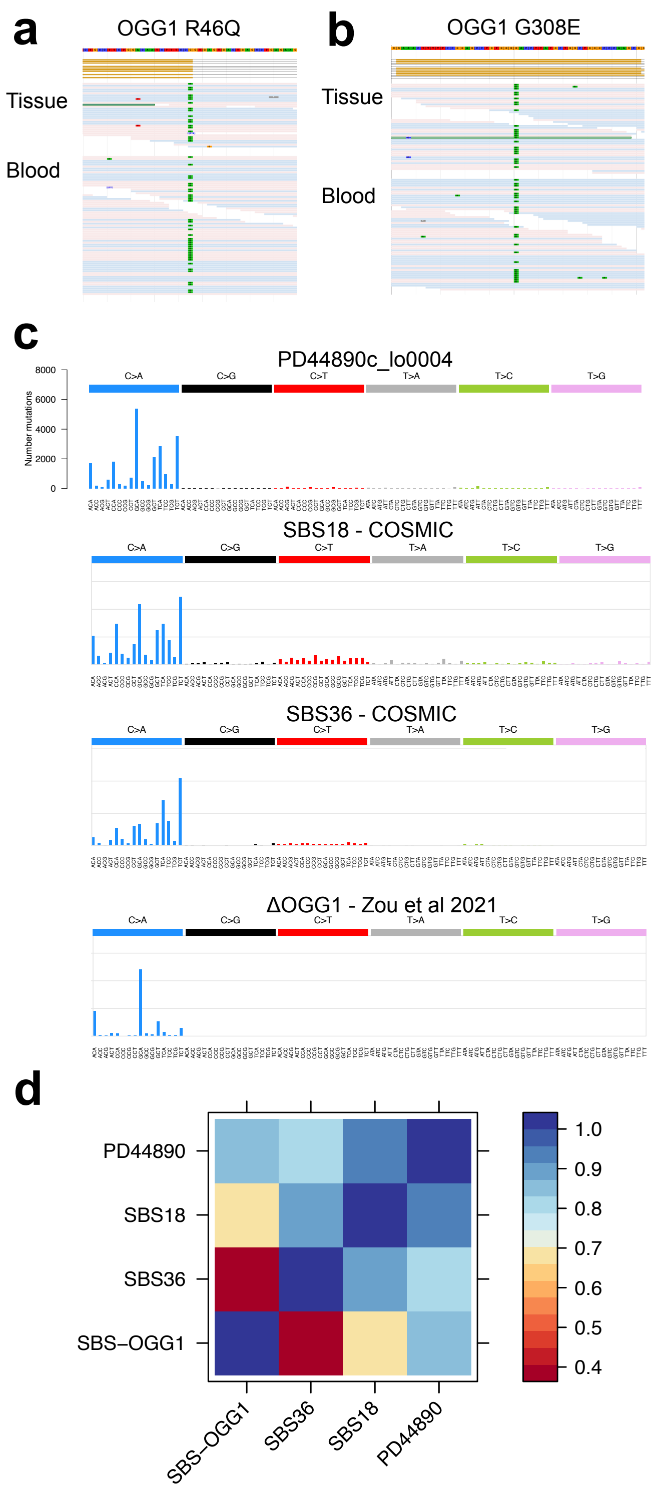

Supplementary Figure 1 | Germline mutations and mutational spectrum of somatic mutations in PD44890
Two candidate germline modifier mutations were identified in individual PD44890 both of which are in the oxoguanine glycosylase

(OGG1) gene which is a key component of the base excision repair pathway and a partner of MUTYH in the repair of 8-oxo-guanine DNA lesions. Both mutations are heterozygous nonsynonymous mutations. JBrowse images are displayed showing the reads for each mutation; **(a)** OGG1 R46Q **(b)** OGG1 R308E. **(c)** Mutational spectrum of SBS mutations from a representative intestinal crypt from individual PD44890, mutational spectra for the COSMIC reference signatures SBS36 and SBS18 are displayed below for comparison (https://cancer.sanger.ac.uk/cosmic/signatures). Mutational spectrum of IPS cells with homozygous *OGG1* deletion (Zou et al 2021, Nature Cancer)[51] denoted SBSOGG1. **(d)** Matrix displaying cosine similarity between the observed spectrum in individual PD44890 and reference signatures SSB18, SBS36 and SBSOGG1.

Supplementary Figure 2

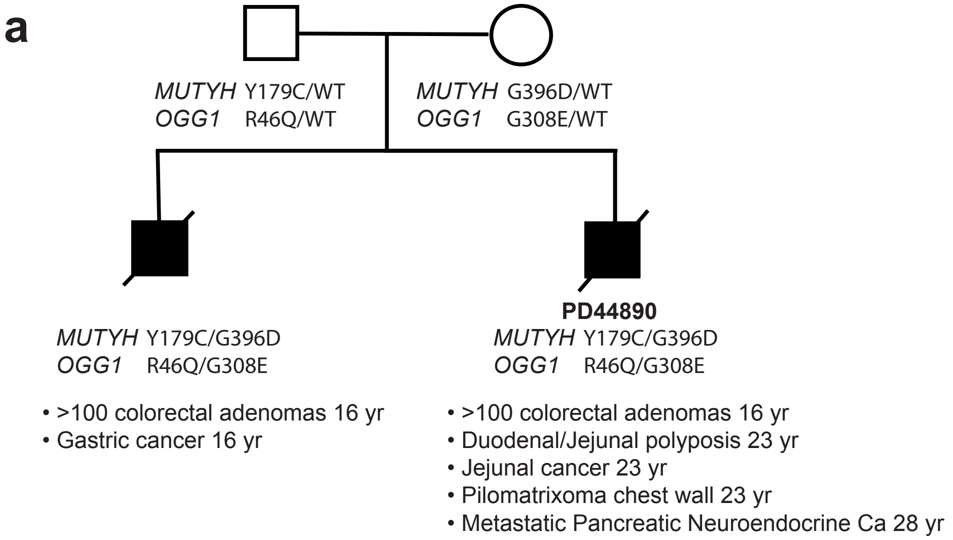

Supplementary Figure 2 | Family pedigree, genotype and phenotype for individual PD44890
(a) Pedigree for the family of individual PD44890. *MUTYH* and *OGG1* genotypes are displayed. The parents of PD44890 carried heterozygous *MUTYH* and *OGG1* mutations and did not display a clinical phenotype. The brother shared the same *MUTYH* and *OGG1* genotype as PD44890 and also developed very early onset gastrointestinal neoplasia.

Supplementary Figure 3

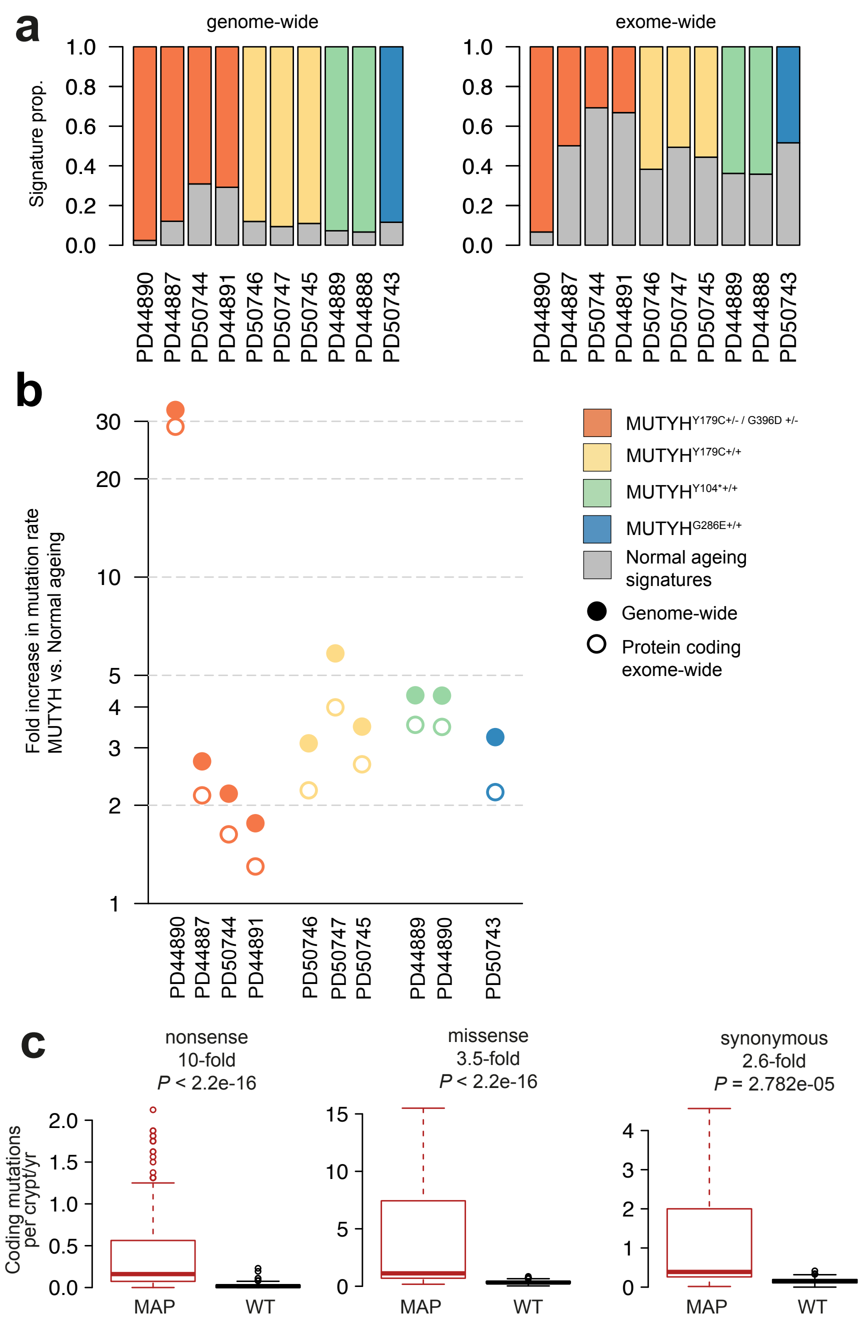

Supplementary Figure 3 | Genome-wide and exome-wide mutation burdens in intestinal crypts

(a) Stacked bar plots showing the proportion of SBS mutations contributed by MUTYH associated signatures, SBS18 and SBS36 (coloured bar) and normal ageing signatures (SBS1 and SBS5) (grey bar). Genome-wide signature proportions (left) protein-coding exome-wide signature proportions (right). **(b)** Fold-increase in the genome- and exome-wide mutation burden of samples from individuals with MUTYH-Associated Polyposis (MAP) compared with normal controls plotted on a logarithmic scale. **(c)** Boxplots showing nonsense, missense and synonymous coding mutation rates (SBS mutations/crypt/year) in histologically normal intestinal crypts from individuals with MUTYH-Associated Polyposis (MAP) (red) and healthy individuals; wild type (WT) (black). Boxplots display median, inter-quartile range (IQR) from 1st to 3rd quartiles and whiskers extend from the last quartile to the last data point that is within 1.5x IQR. Fold changes compared to WT, are shown above each pair, *P*-values calculated using a two-sided Wilcoxon test. *P*-values, $<2.2*10^{-16}$, $<2.2*10^{-16}$ and $2.8*10^{-05}$ for nonsense, missense and synonymous mutations

respectively. Data for healthy wild-type individuals is from Lee-Six et al 2019[3]

Supplementary Figure 4

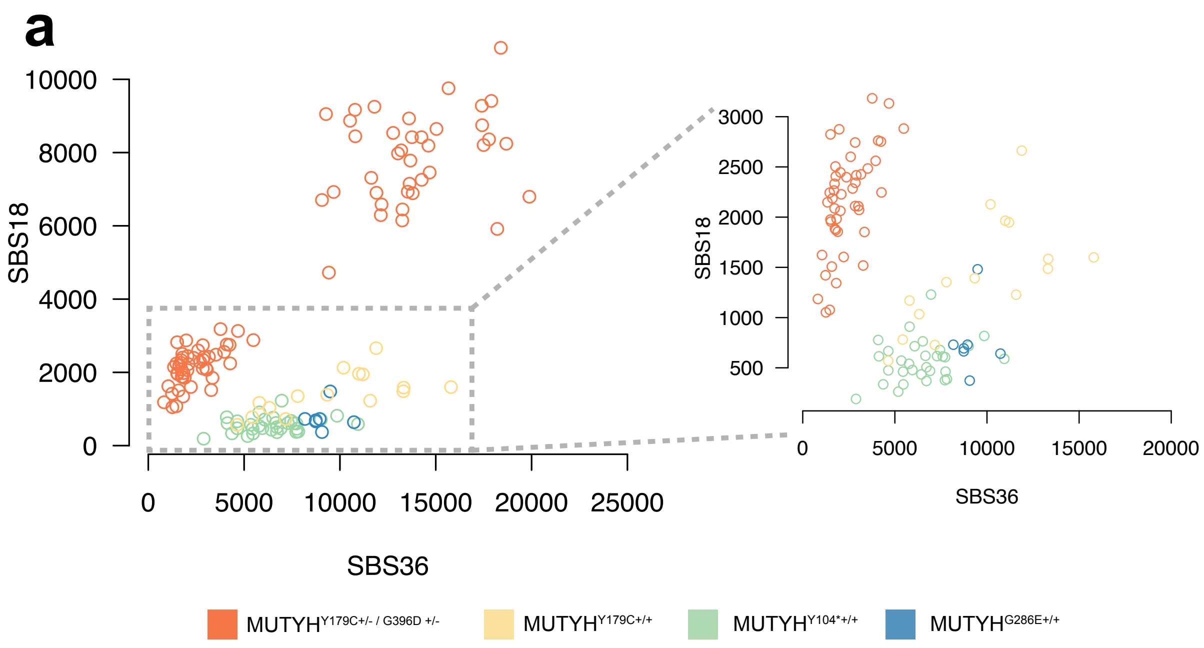

Supplementary Figure 4 | Mutational signatures in normal intestinal crypts

(a) Somatic SBS mutation burden in normal intestinal crypts due to SBS36 (x-axis) and SBS18 (y-axis) coloured according to germline mutation (orange; MUTYH$^{Y179C+/- \ G396D+/-}$, yellow; MUTYH$^{Y179C+/+}$, green; MUTYH$^{Y104*+/+}$, blue; MUTYH$^{G286E+/+}$). Crypts with MUTYH$^{Y179C+/+}$, MUTYH$^{Y104*+/+}$ and MUTYH$^{G286E+/+}$ show greater relative increases in SBS36 whereas those with MUTYH$^{Y179C+/- \ G396D+/-}$ show greater increases in SBS18. Crypts from individual PD44890 are a clear outlier with much greater SBS18 burdens. Magnified plot inset.

Supplementary Figure 5

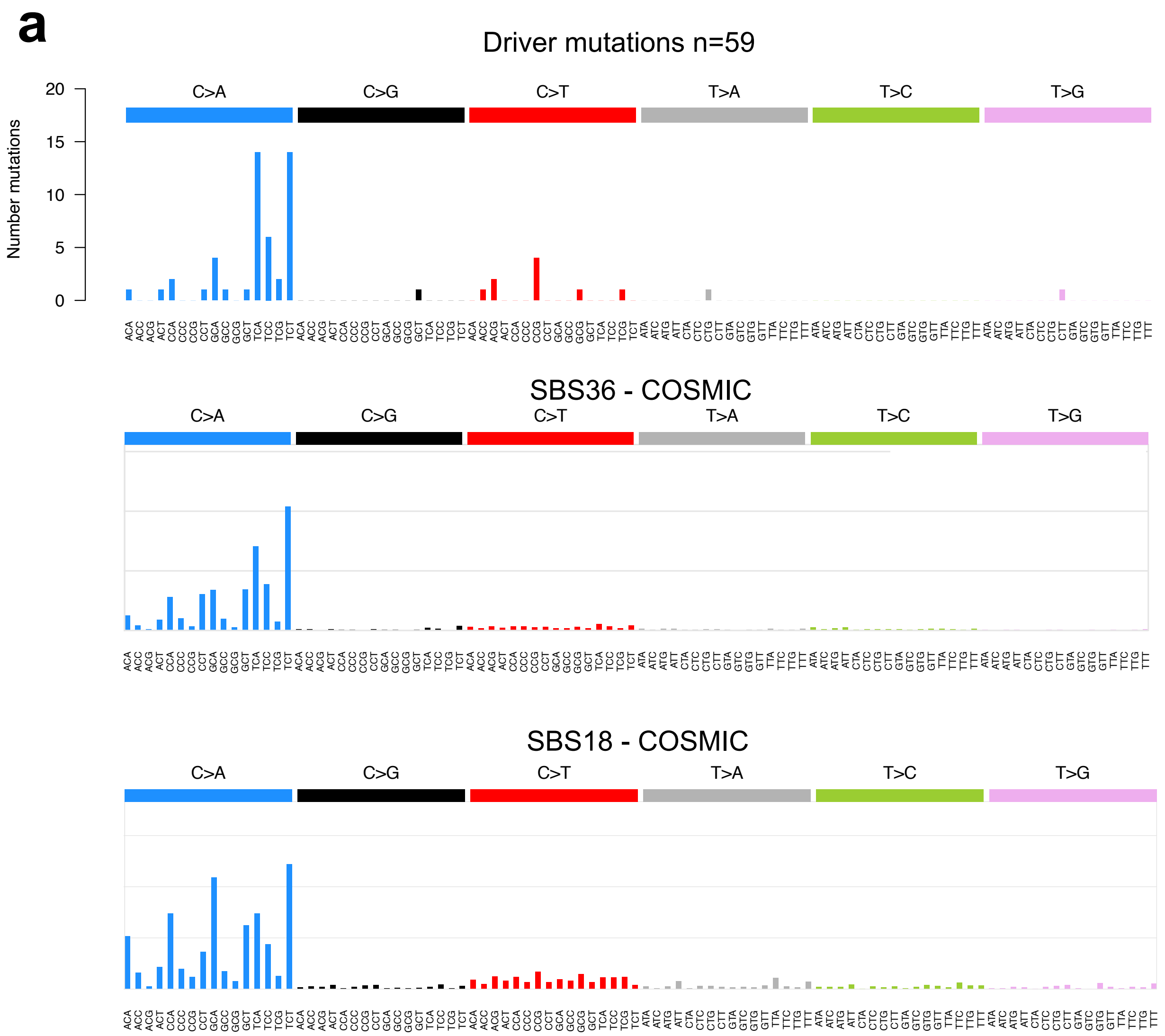

Supplementary Figure 5 | Mutational spectrum of driver mutations in normal and neoplastic intestinal crypts
(a) Mutational spectrum of driver mutations identified in normal and neoplastic intestinal stem cells in this cohort (n=59). Mutational spectra for the COSMIC reference signatures SBS36 and SBS18 are displayed below for comparison. The spectrum of driver mutations is dominated by SBS36 and SBS18. COSMIC reference signatures available at: https://cancer.sanger.ac.uk/cosmic/signatures.

Supplementary Figure 6

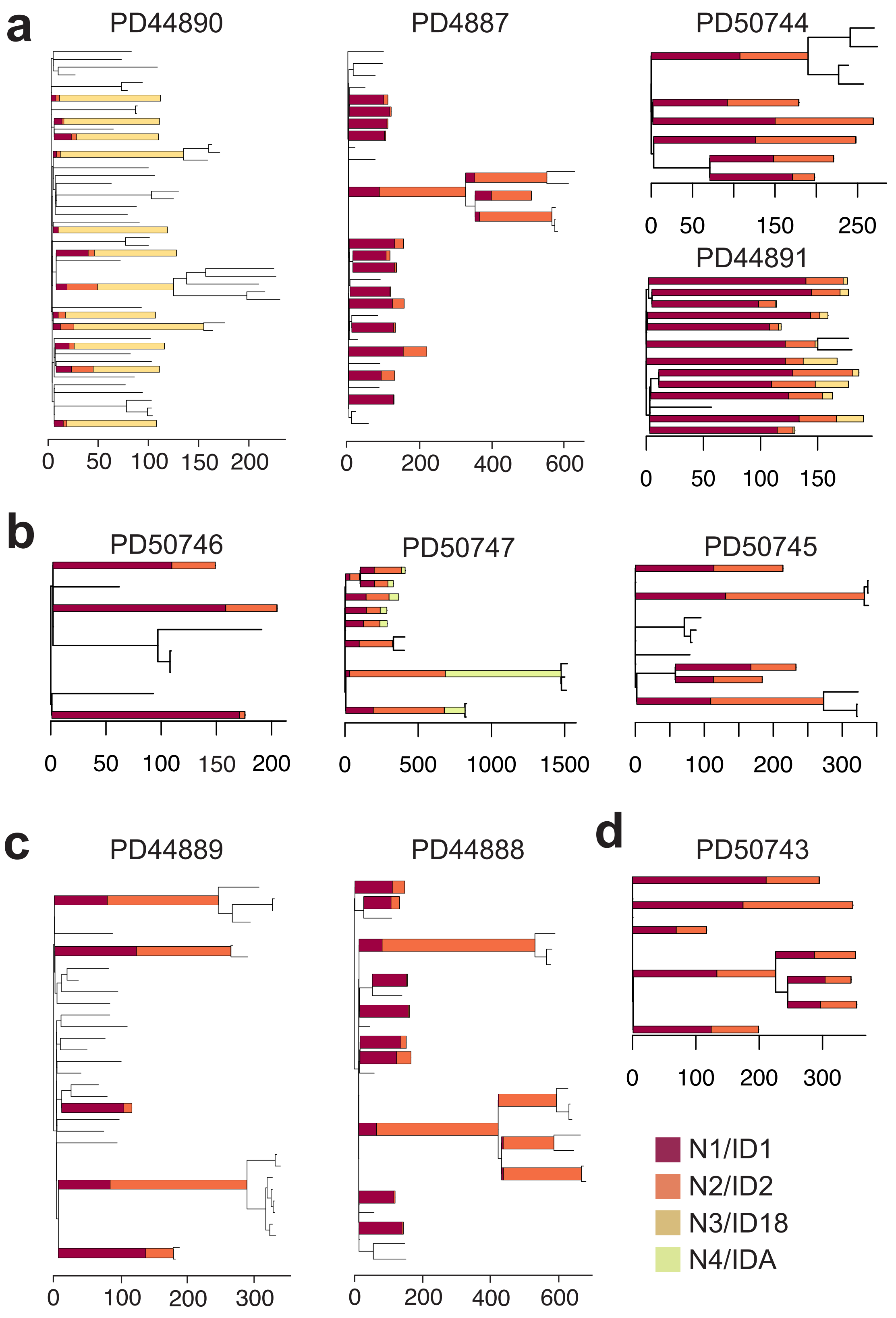

Supplementary Figure 6 | Phylogenetic trees showing ID signature exposure

Phylogenetic trees with ID signature exposure overlaid. Branches of the phylogenetic tree with >100 somatic ID mutations have been converted into stacked bar plots displaying the contribution of each HDP signature component / mutational process to that branch: N1/ID1 shown in dark red, N2/ID2 in orange, N3/ID18 in mustard yellow and N4/IDA in lime green. Phylogenetic trees are arranged according to their germline genotype, **(a)** $MUTYH^{Y179C+/-\ G396D+/-}$, **(b)** $MUTYH^{Y179C+/+}$, **(c)** $MUTYH^{Y104*+/+}$ and **(d)** $MUTYH^{G286E+/+}$

Supplementary Figure 7

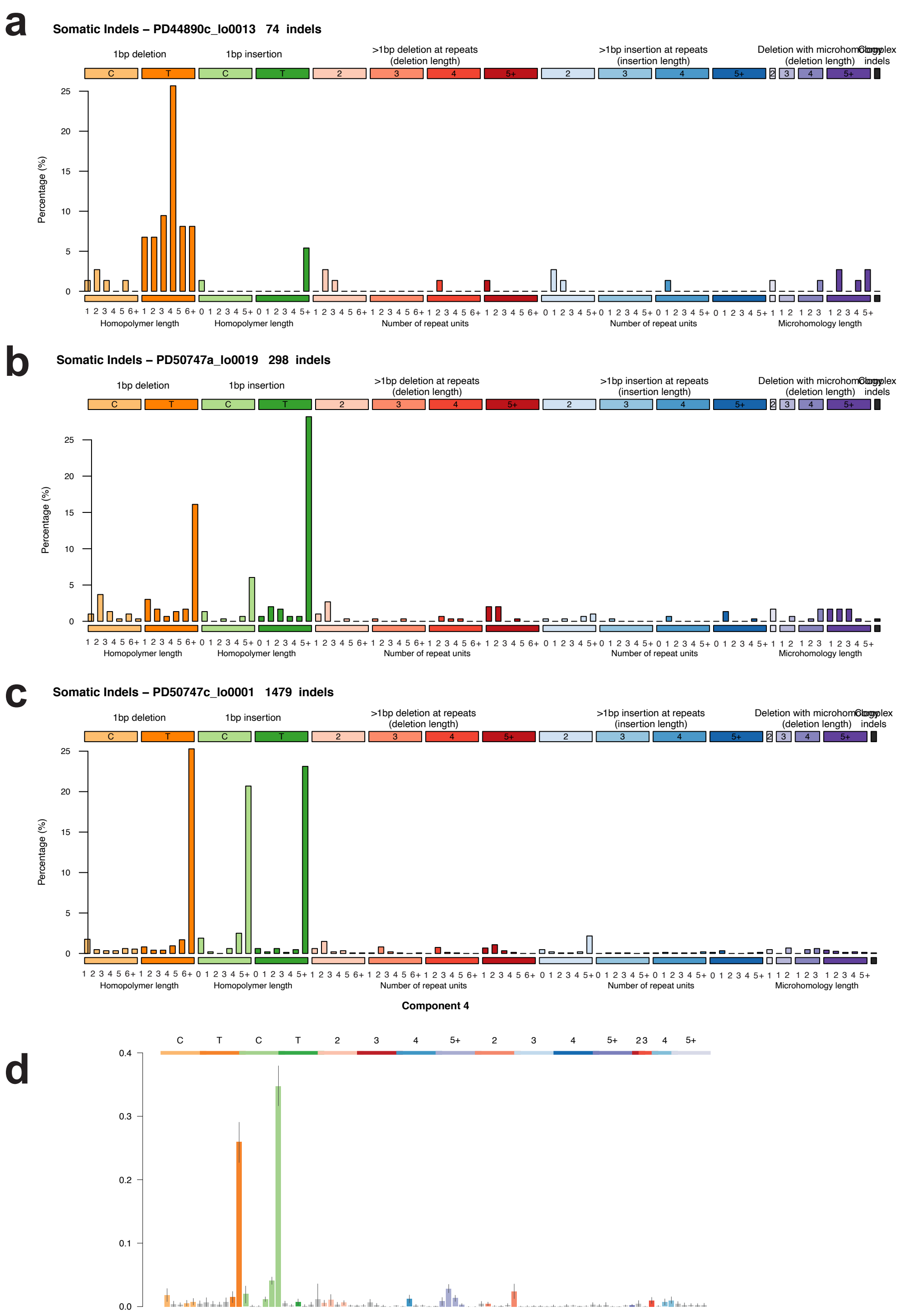

Supplementary Figure 7 | ID mutational spectra

ID mutational spectra for crypts with sporadic ID signature exposures. **(a)** Representative sample from PD44890 with high ID18 exposure. Representative samples from PD50747 showing IDA exposure in **(b)** normal crypt and **(c)** adenoma gland. **(d)** ID signature component arising from unconditioned *de novo* HDP signature extraction (Methods) corresponding to signature IDA. Credibility intervals (95%) are displayed for each bar which are calculated from the set of posterior samples (n=2500) collected

during the algorithm’s computation.

Supplementary Figure 8

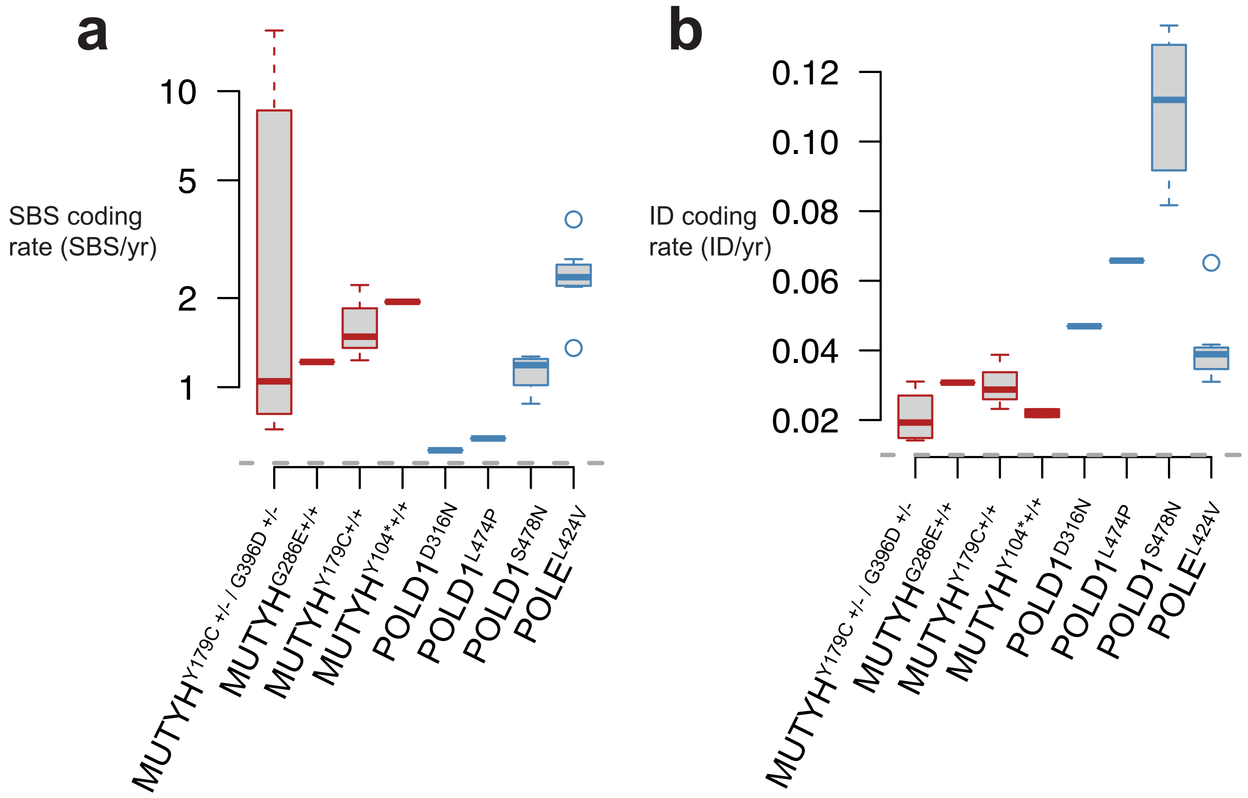

Supplementary Figure 8 | Coding mutation burdens in intestinal crypts from individuals with germline MUTYH and POLE/D1 mutations

Coding mutation burdens in individuals with germline MUTYH and POLE/D1 mutations.**(a)** SBS coding mutation rate (SBS/year) coloured according to cohort; MUTYH; red, POLE/D1; blue. **(b)** ID coding mutation rate (ID/year). Boxplots display median, inter-quartile range (IQR) from 1st to 3rd quartiles and whiskers extend from the last quartile to the last data point that is within 1.5x IQR.

Supplementary Note 1 - Mutational Signature Analysis

HDP SBS Signature components

A total of 9 signature components were extracted HDP0-HDP8. Plots show probability distributions of extracted components. Trinucleotide context (x-axis) and proportion (y-axis). Credibility intervals (95%) are displayed for each bar which are calculated from the set of posterior samples (n=2500) collected during the algorithm's computation. Trinucleotide contexts which are not statistically significant are shown in light grey.

Supplementary Figure 9 - SBS HDP N0

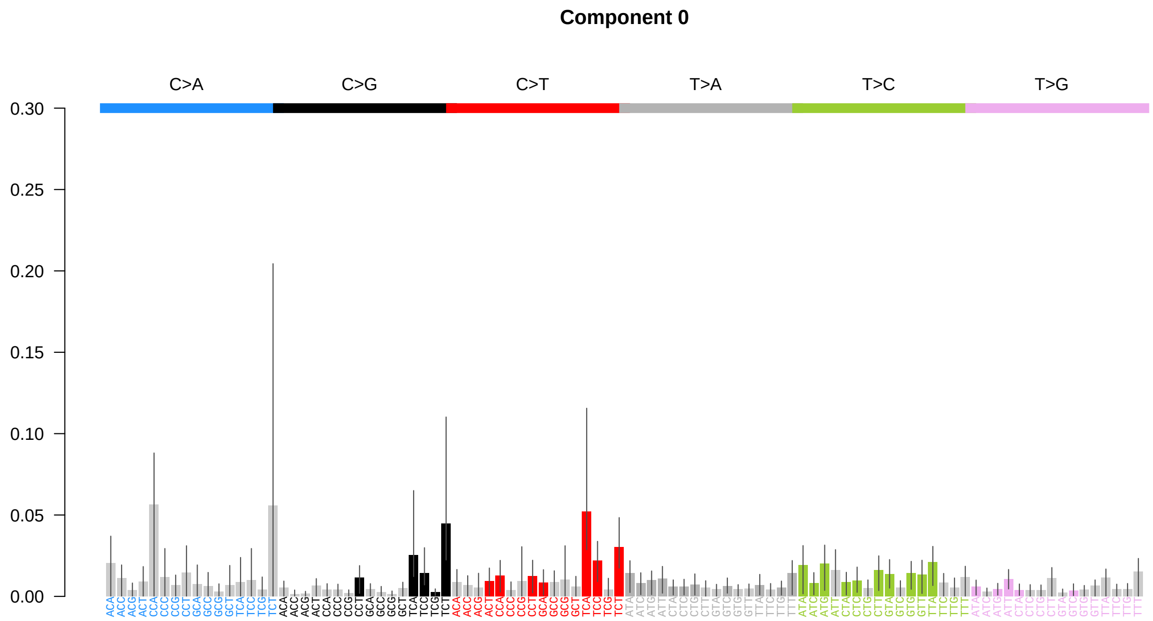

Supplementary Figure 10 - SBS HDP N1

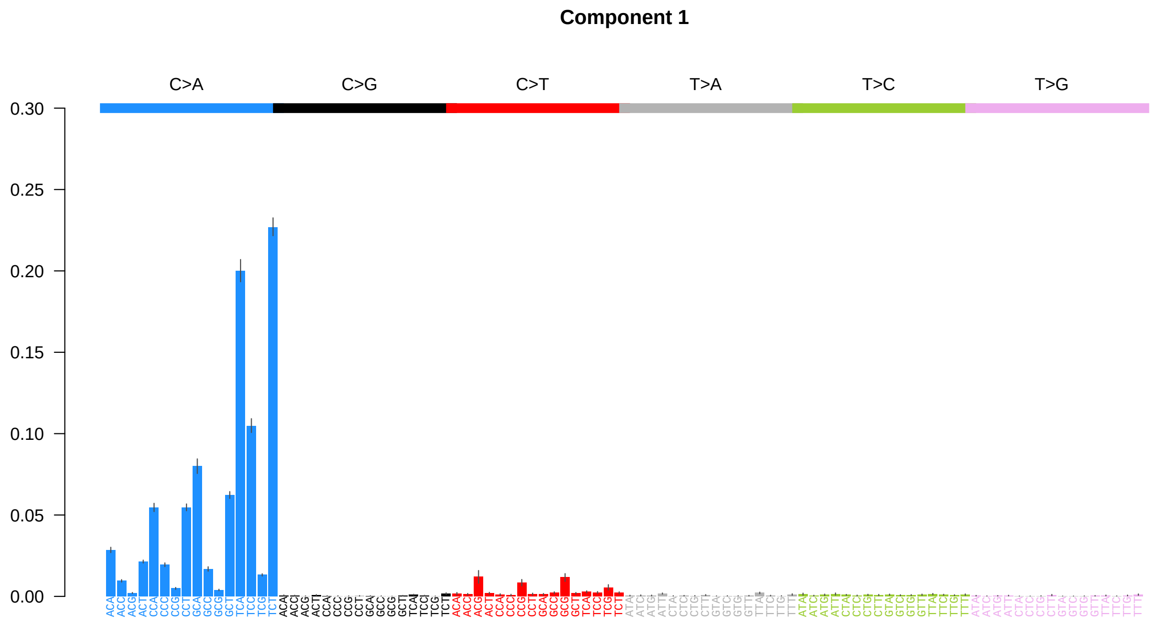

Supplementary Figure 11 - SBS HDP N2

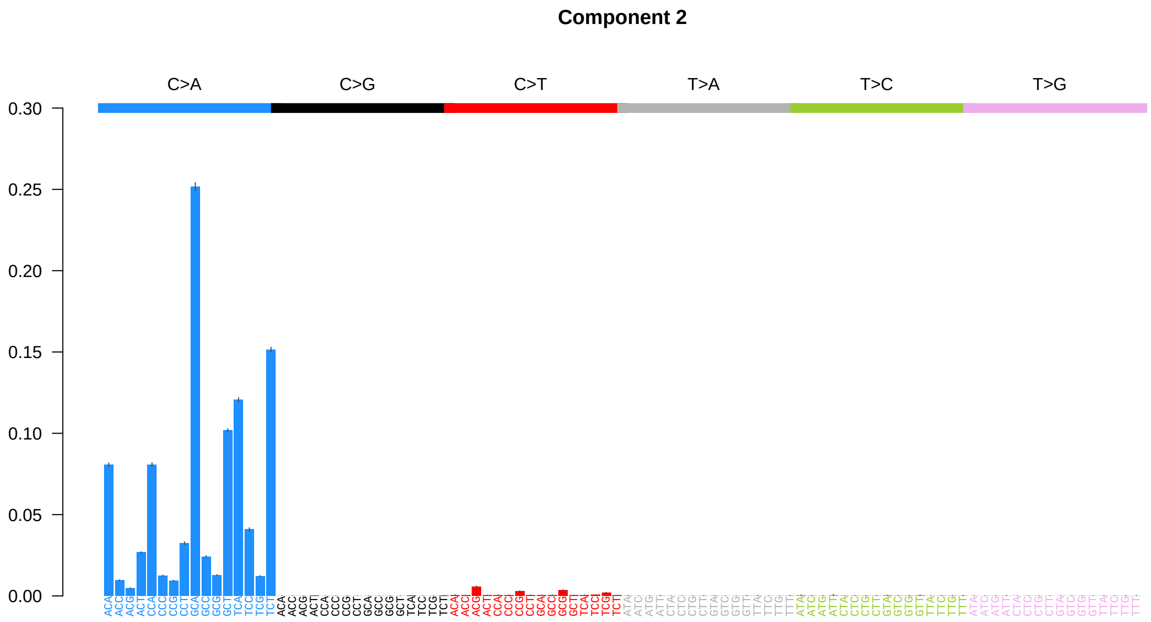

Supplementary Figure 12 - SBS HDP N3

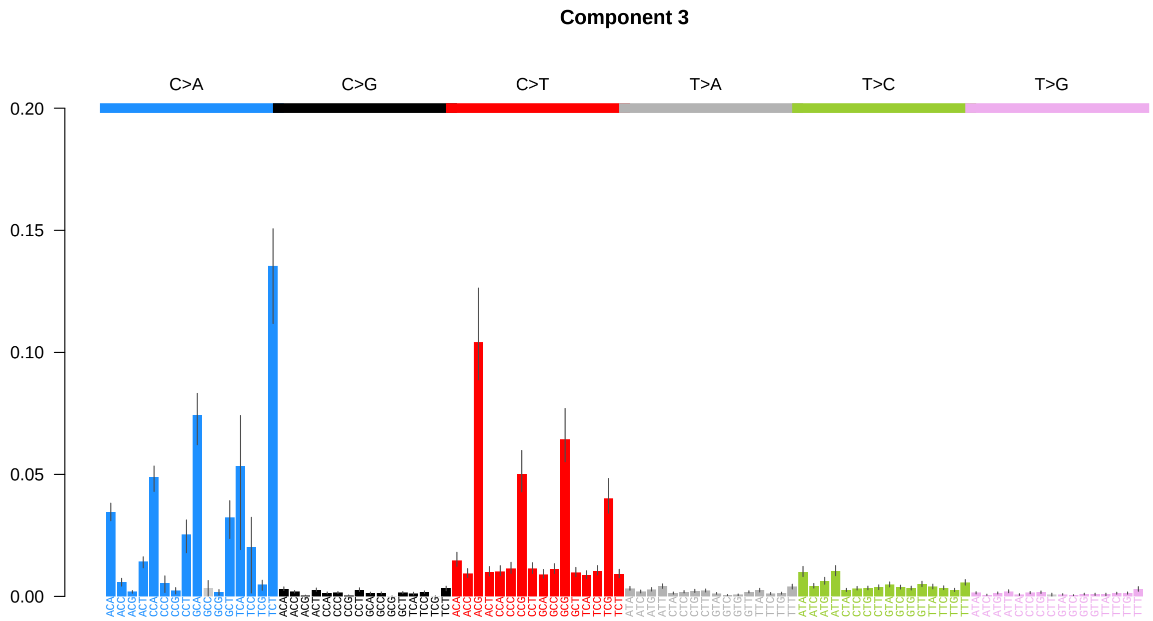

Supplementary Figure 13 - SBS HDP N4

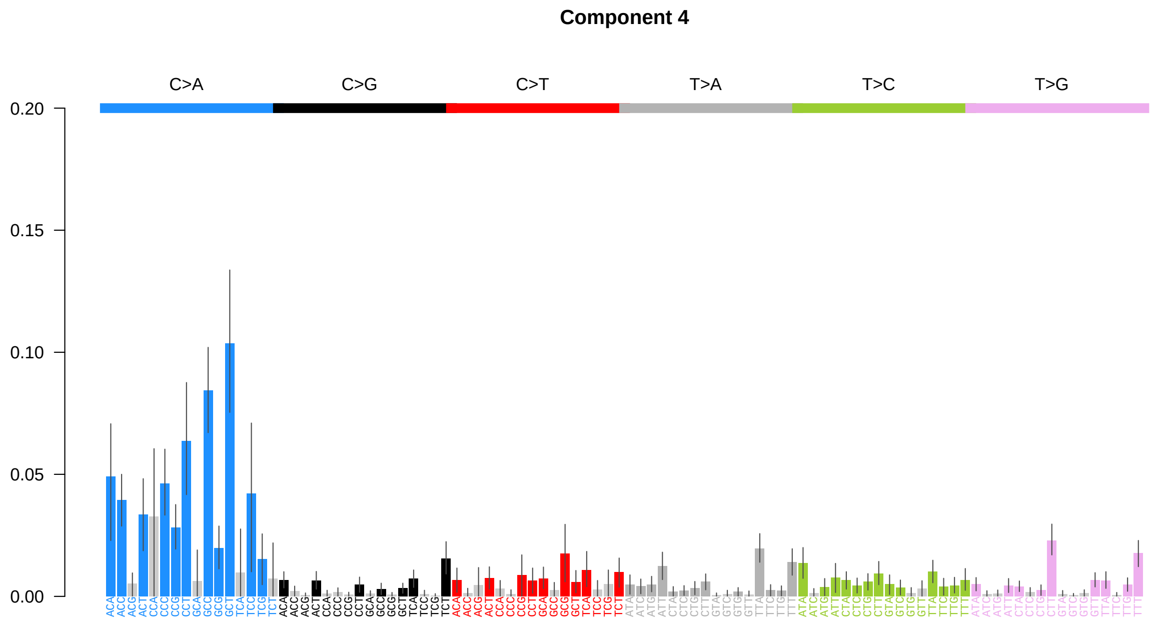

Supplementary Figure 14 - SBS HDP N5

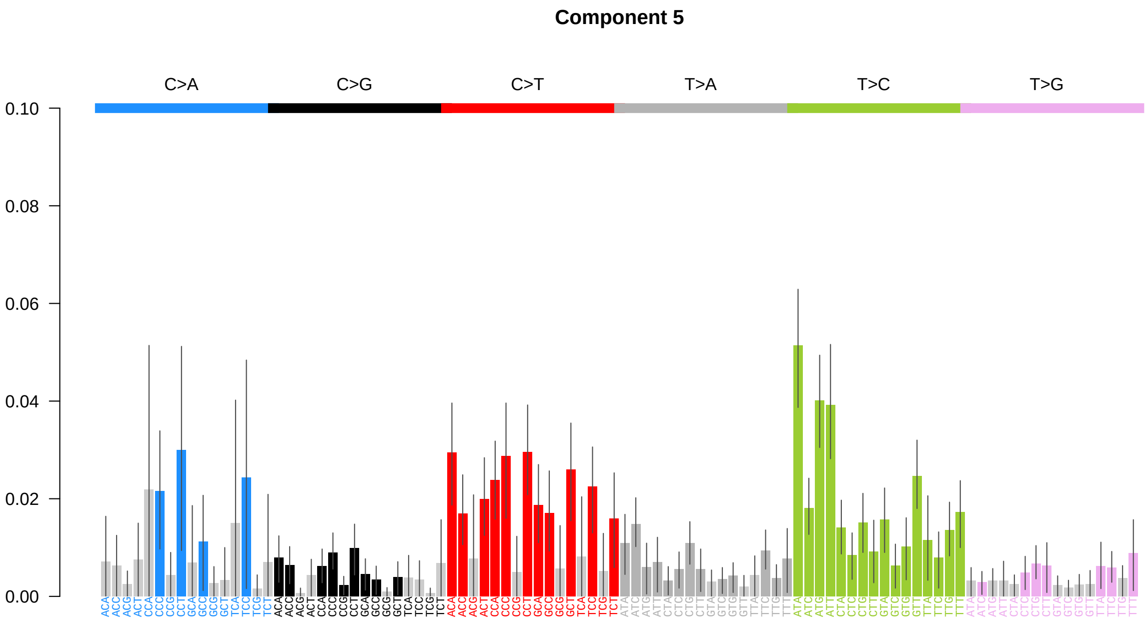

Supplementary Figure 15 - SBS HDP N6

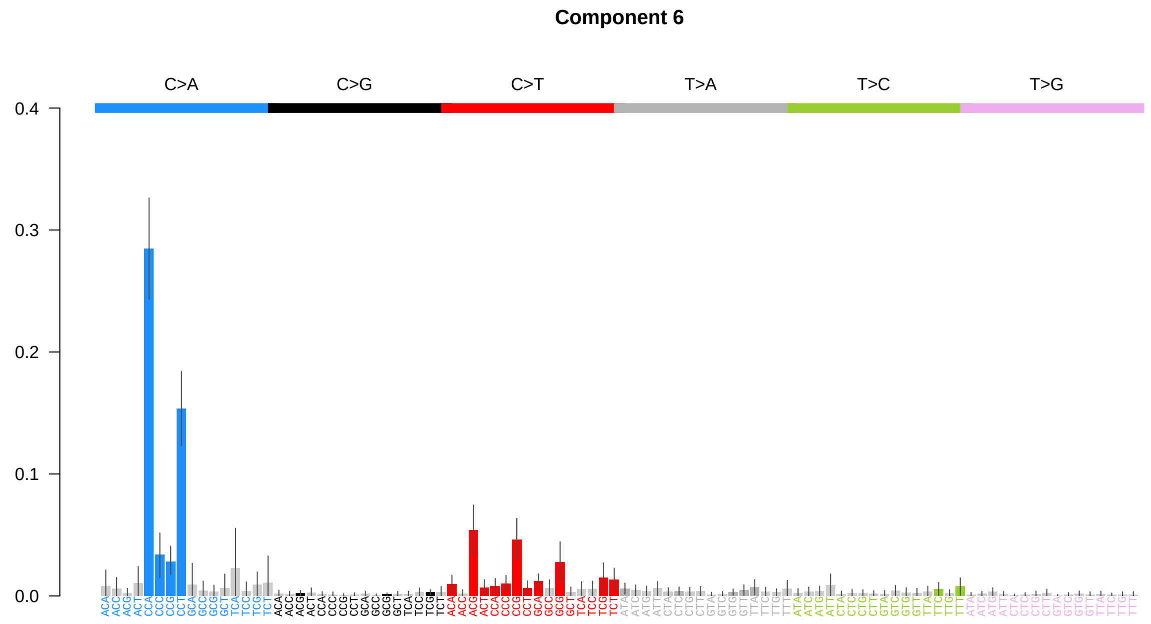

Supplementary Figure 16 - SBS HDP N7

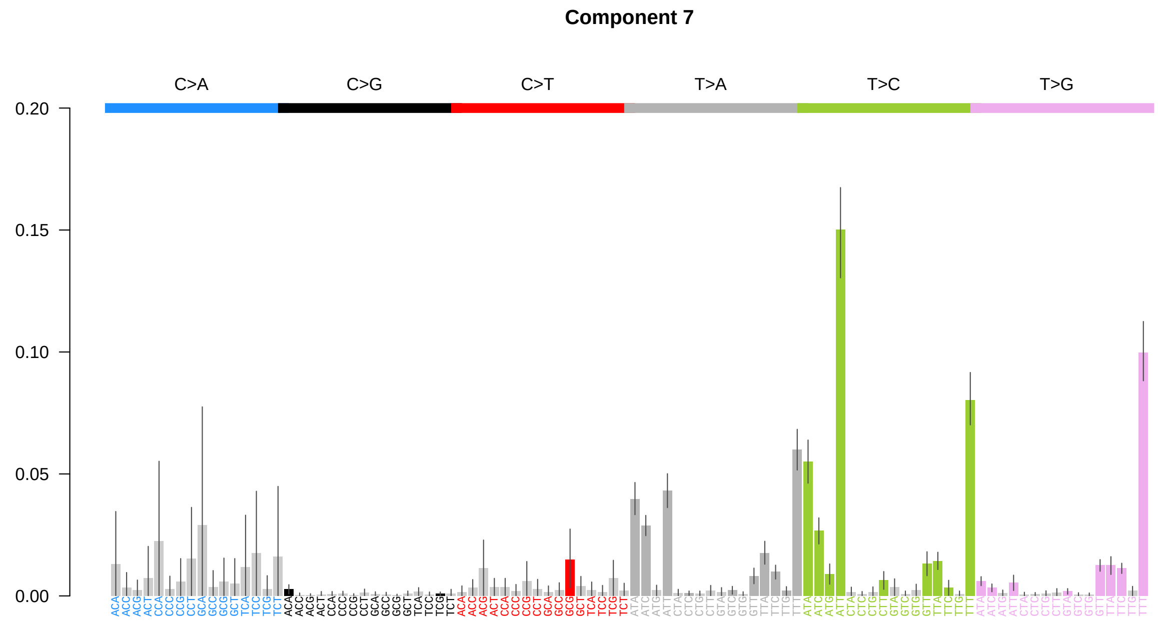

Supplementary Figure 17 - SBS HDP N8

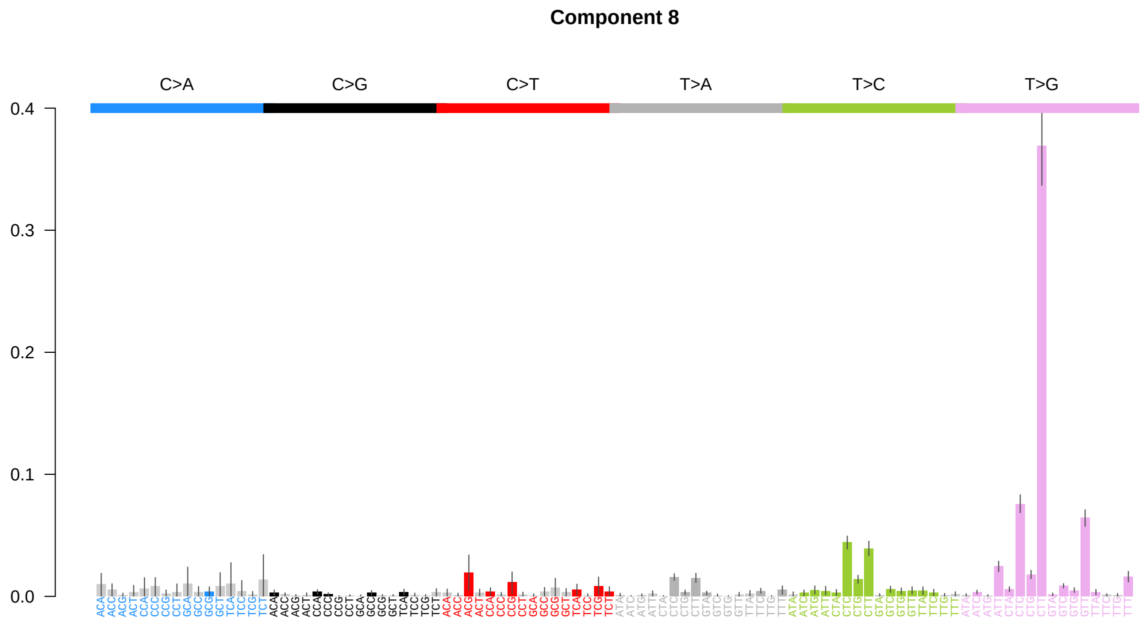

HDP SBS Signature components - deconvolution

Signature deconvolution was performed on signature components identified in signature extraction. Deconvolution was performed on HDP components that contained known signatures. Reference signatures from the PCAWG database were used for deconvolution (Methods).

Plots show mutational spectra of extracted component; trinucleotide context (x-axis) and proportion (y-axis). Each HDP component is displayed in the uppermost panel followed by the reconstituted component after deconvolution and its cosine similarity to the original HDP component. PCAWG components and their relative contributions are displayed on the lower panels.

Supplementary Figure 18 - SBS HDP N2

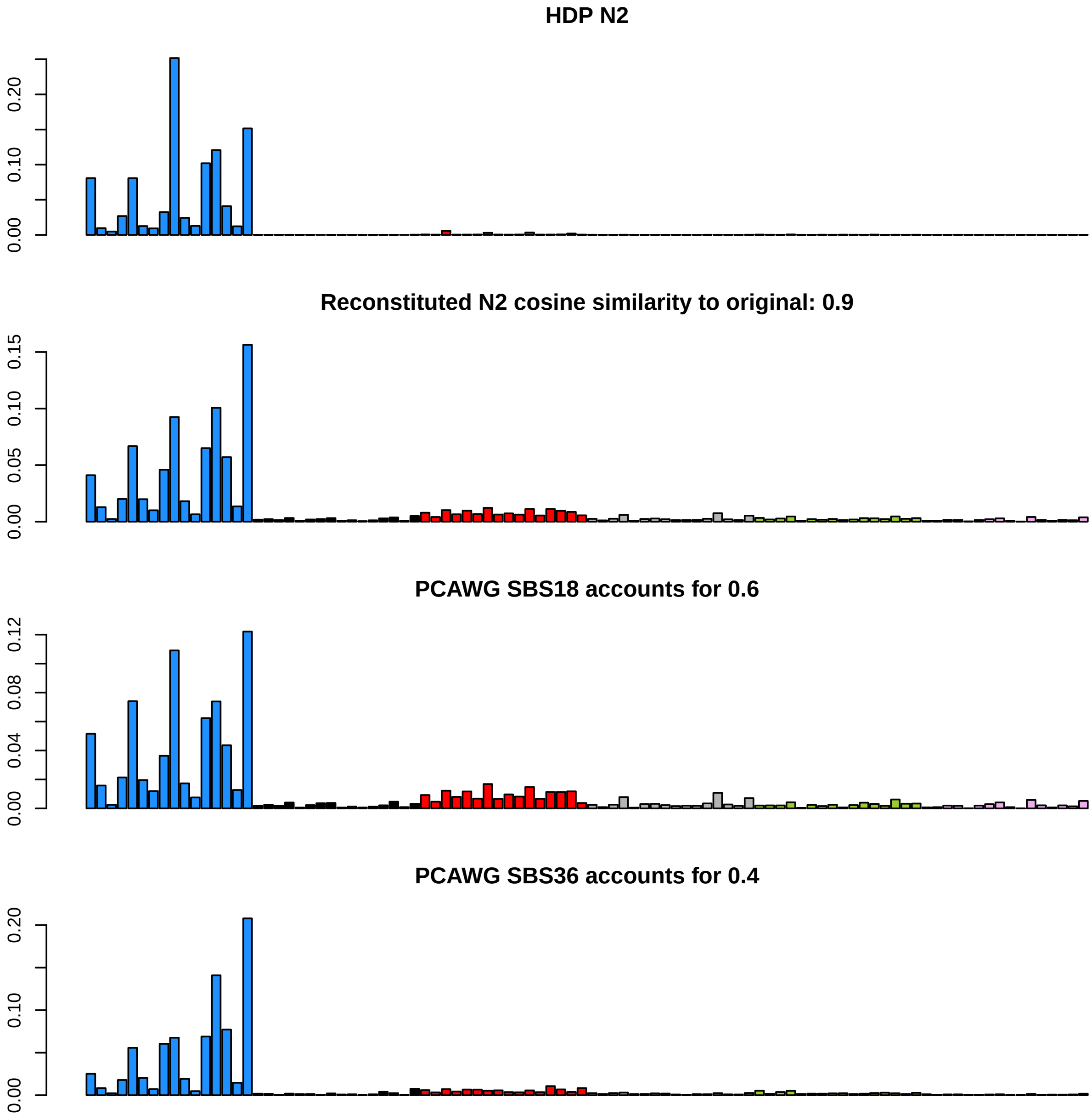

Supplementary Figure 19 - SBS HDP N3

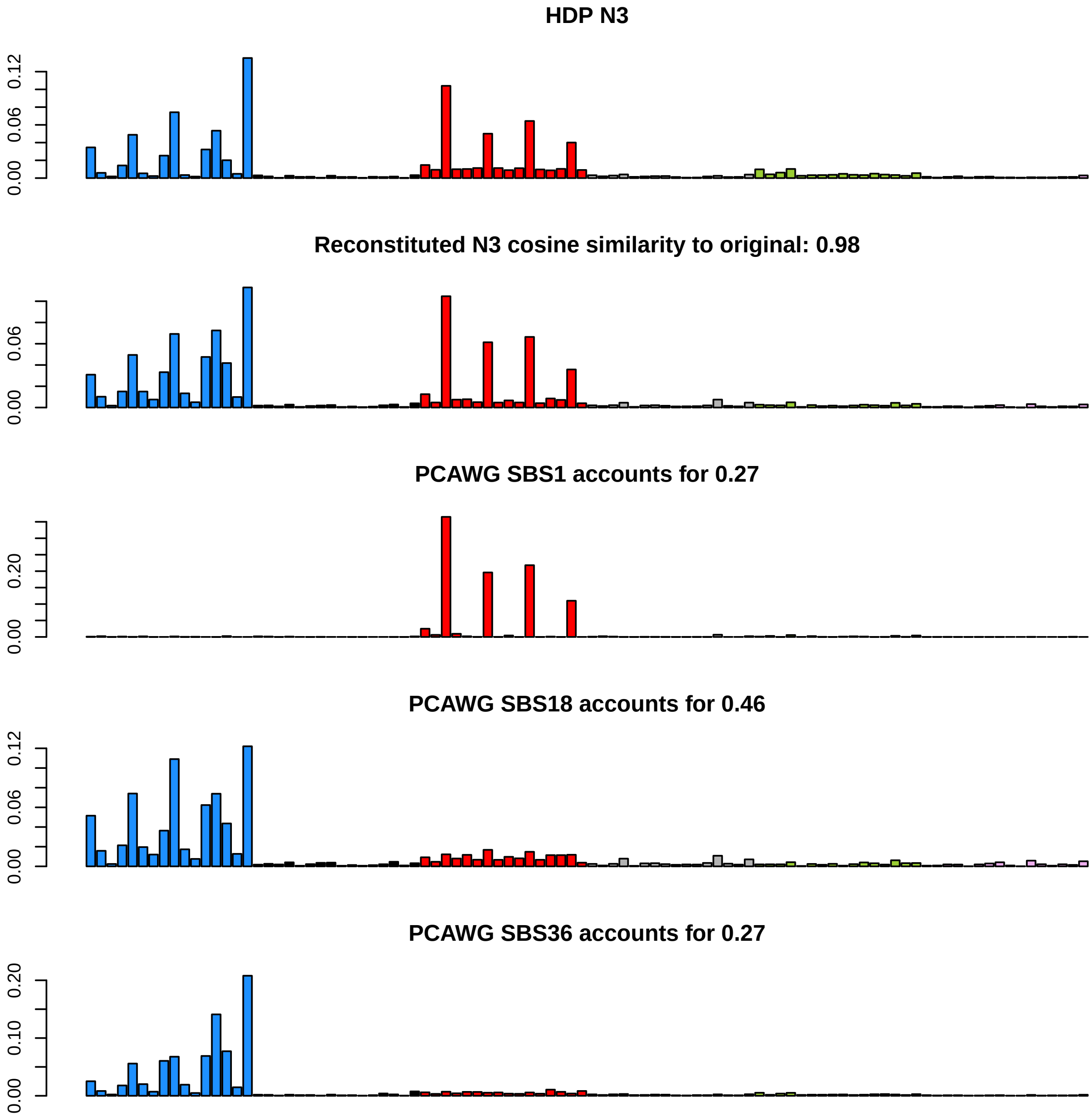

Supplementary Figure 20 - SBS HDP N4

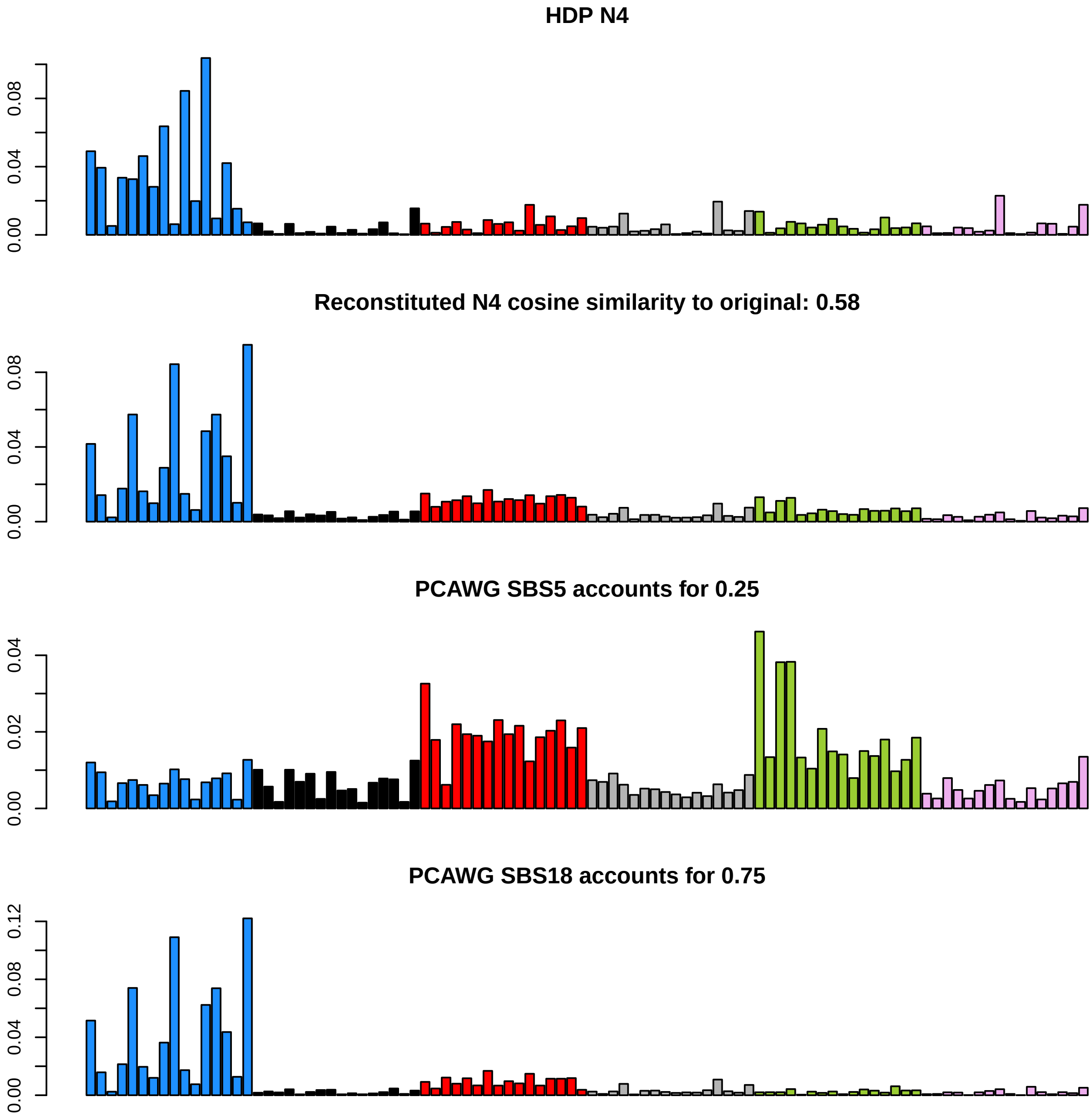

Supplementary Figure 21 - SBS HDP N7

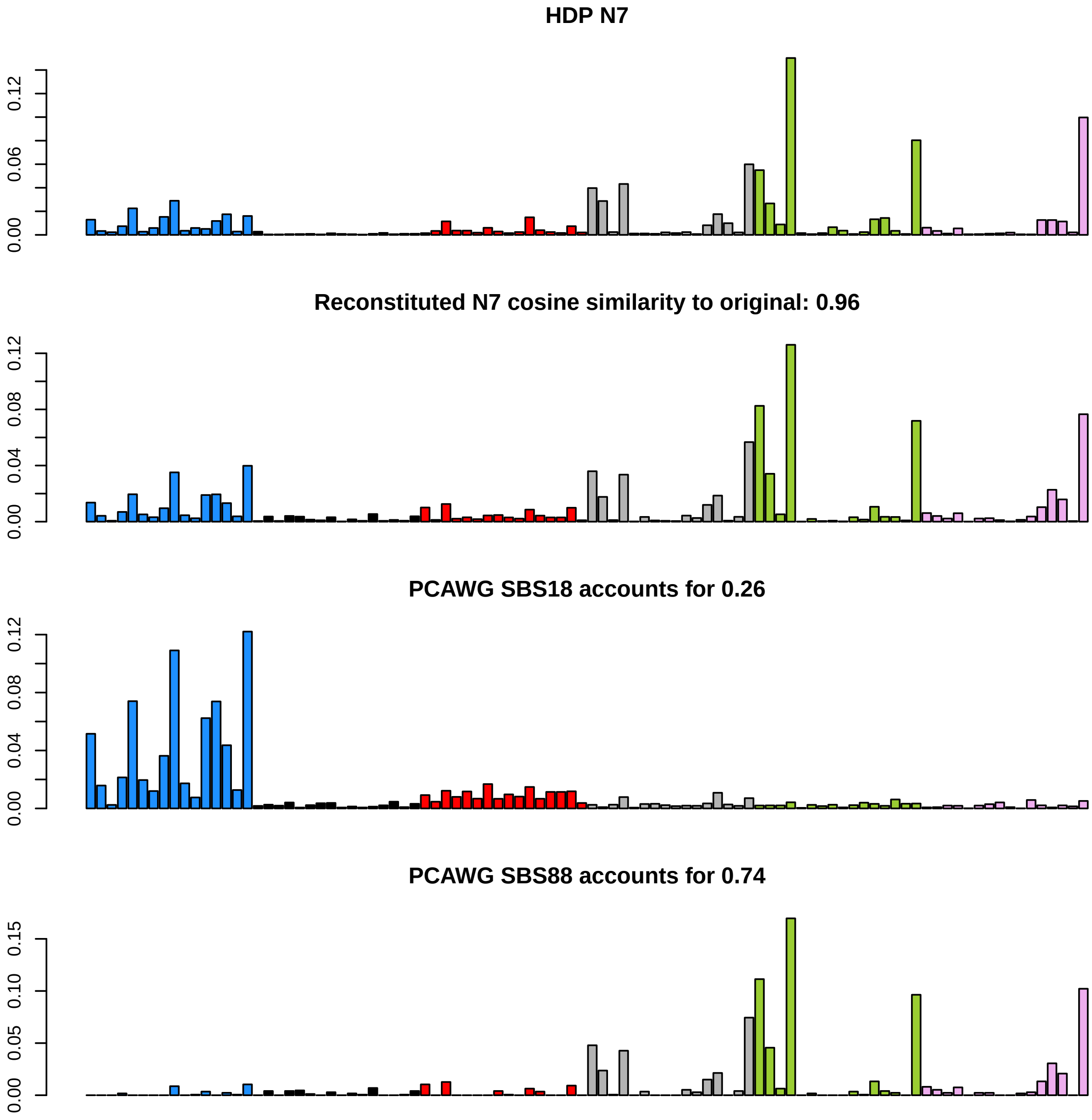

Comparison of HDP and Sigprofiler *de novo* signature extraction

Comparison of signature extraction with HDP and SigProfiler was performed (methods). No new signatures were identified using SigProfiler and more mixing between signature components was observed using Sigprofiler compared with HDP.

HDP and Sigprofiler parameters are outlined above.

Plots show mutational spectra of extracted components showing trinucleotide context (x-axis) and proportion (y-axis). Credibility intervals (95%) are displayed for each bar which are calculated from the set of posterior samples (n=2500) collected during the algorithm's computation. Trinucleotide contexts whose contribution is not statistically significant are shown as light grey bars.

Supplementary Figure 22 - HDP Components

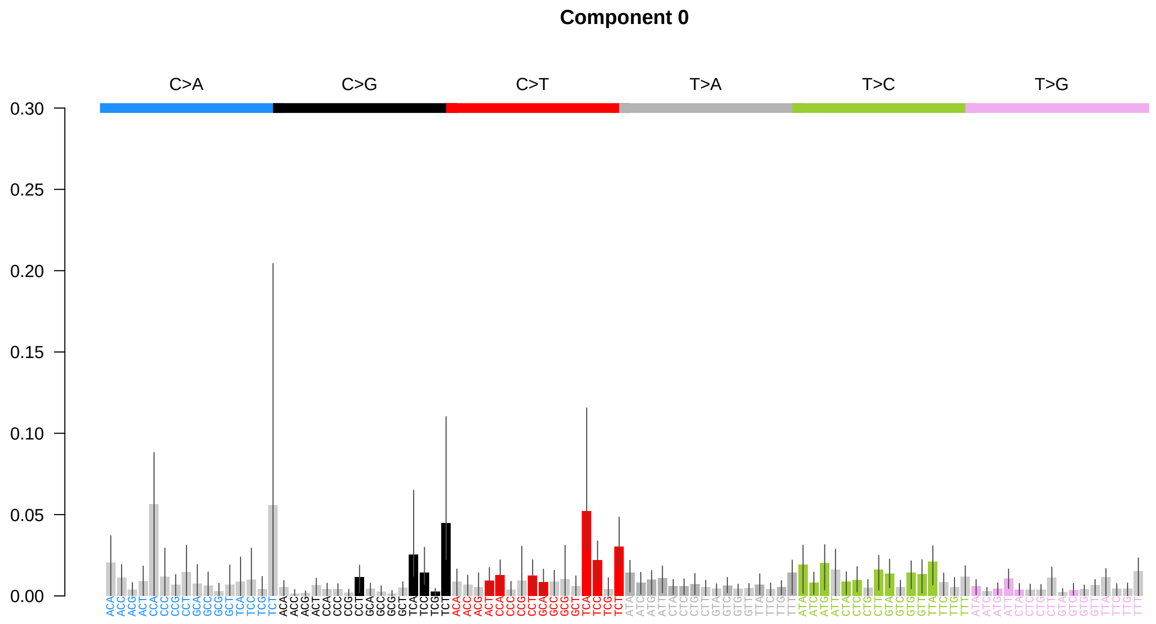

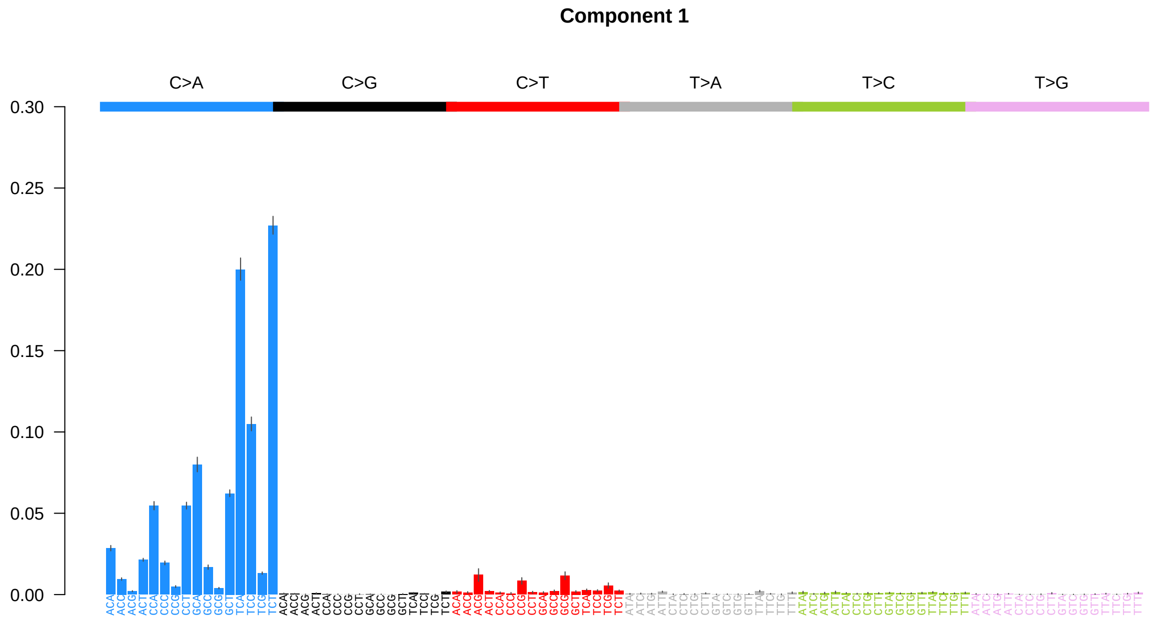

Component 2

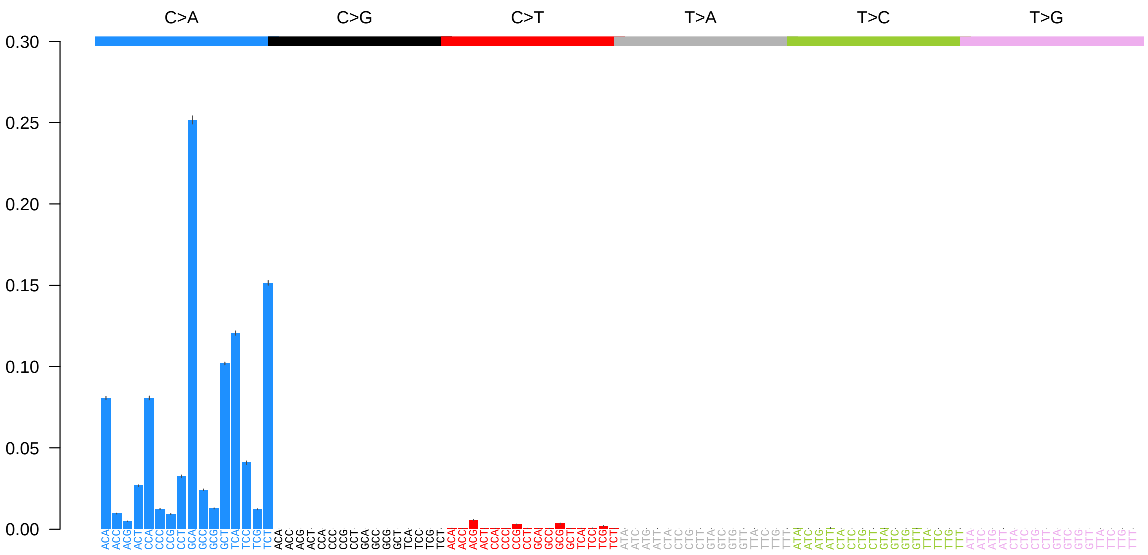

Component 3

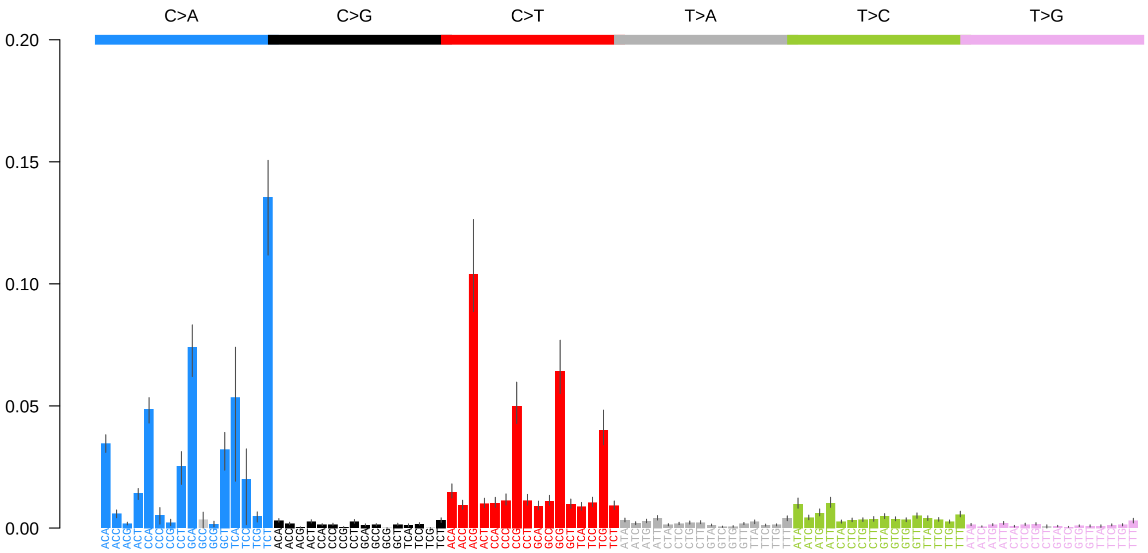

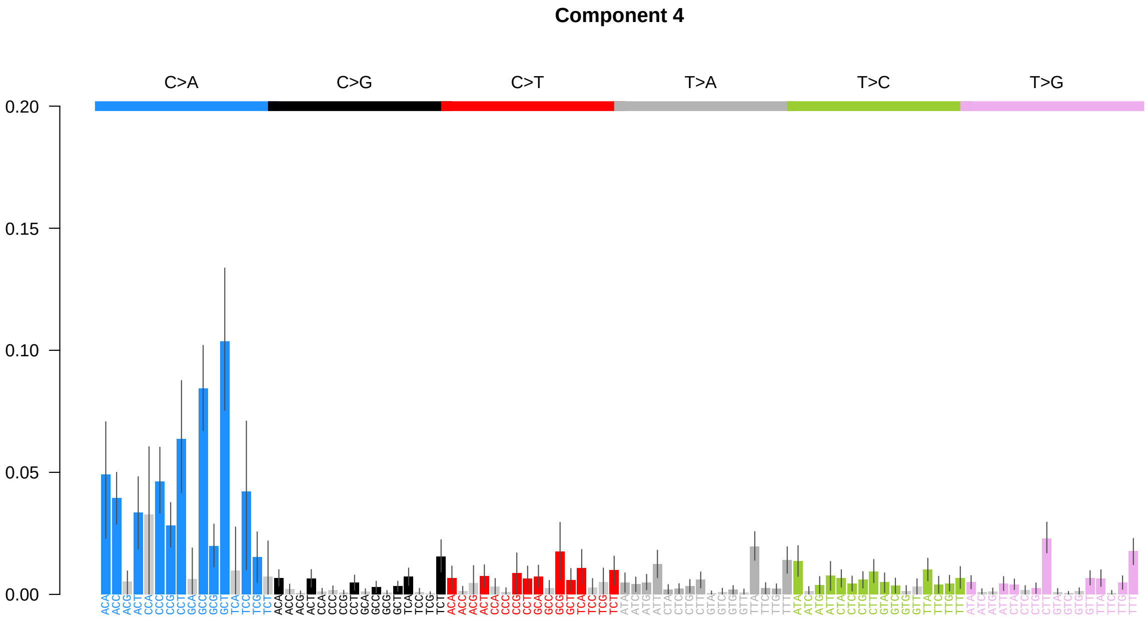
Component 4
C>A
C>G
C>T
T>A
T>C
T>G
0.20
0.15
0.10
0.05
0.00

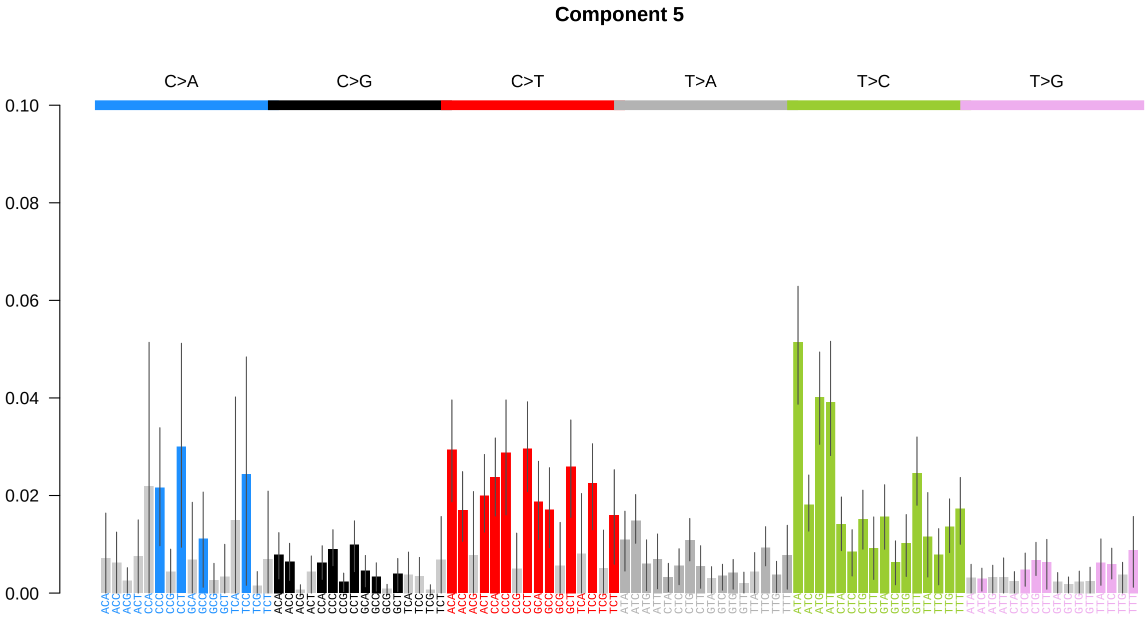
Component 5
C>A
C>G
C>T
T>A
T>C
T>G
0.10
0.08
0.06
0.04
0.02
0.00

Component 6

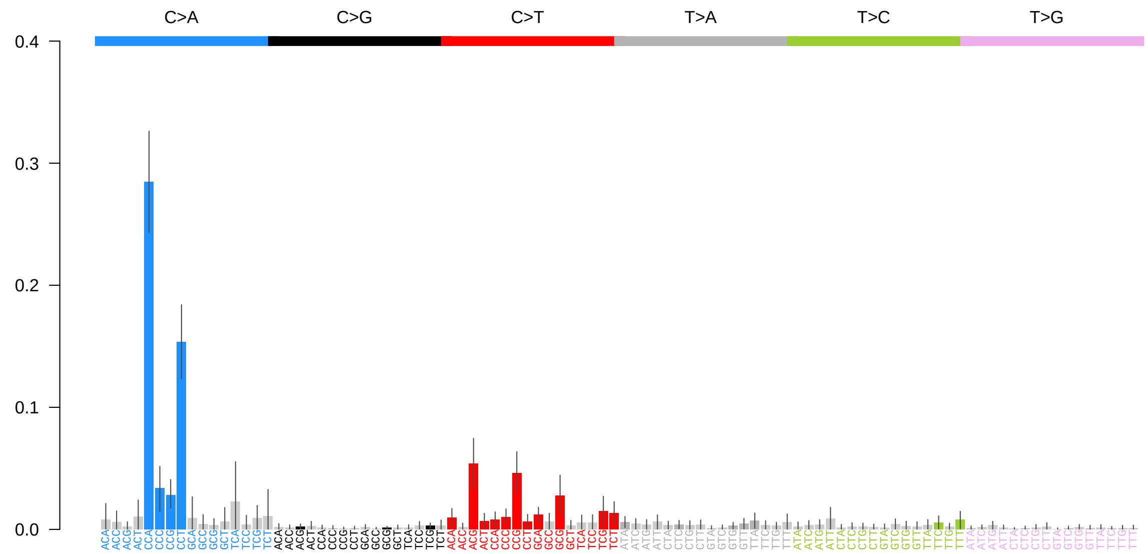

Component 7

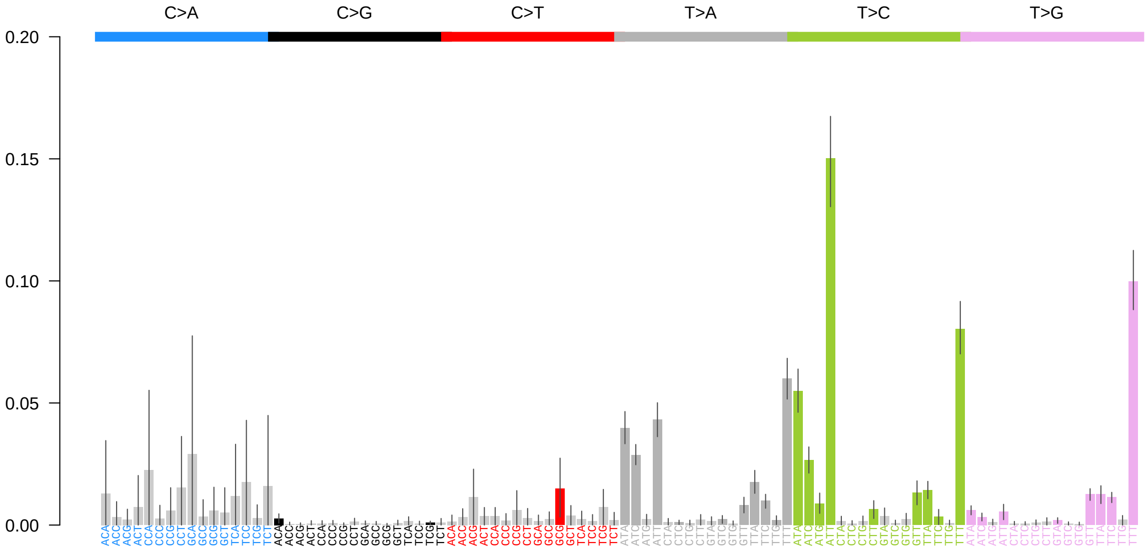

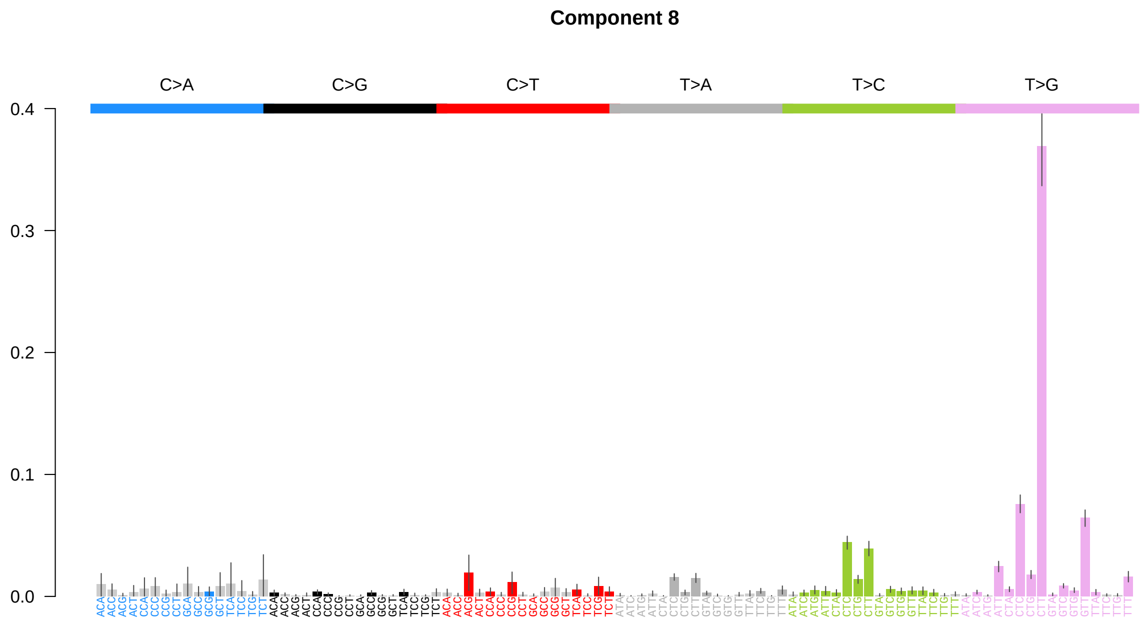
Component 8
C>A
C>G
C>T
T>A
T>C
T>G
0.4
0.3
0.2
0.1
0.0

Supplementary Figure 23 - SigProfiler Components

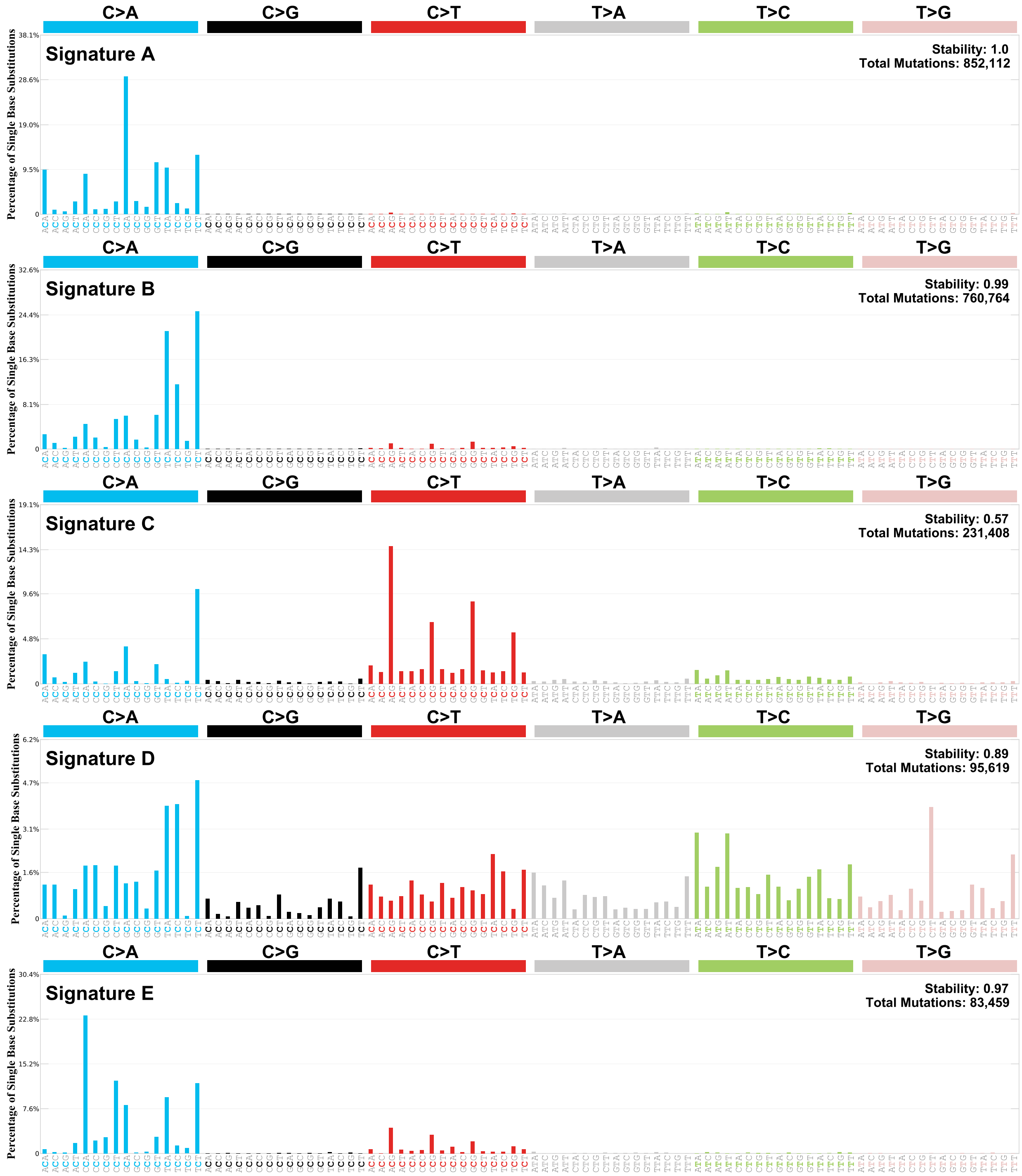

Summary of SBS signature component exposure

Heatmaps showing the proportion of mutations attributable too each extracted signature component organised per-patient. HDP (above) and SigProfiler (below).

Supplementary Figure 24 - HDP SBS signature exposure

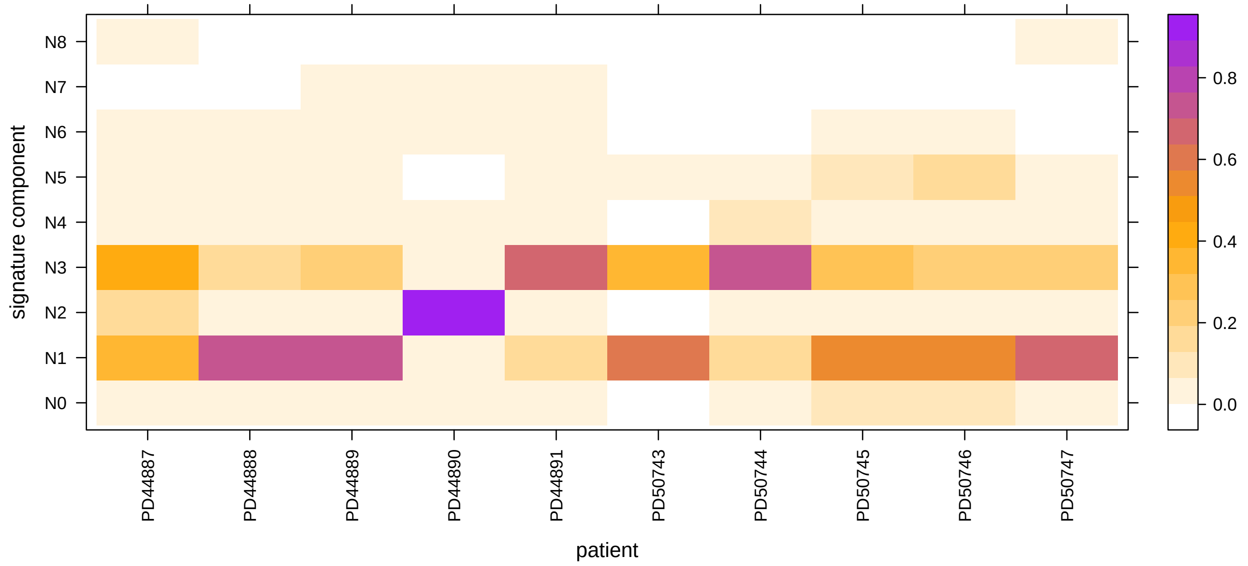

Supplementary Figure 25 - SigProfiler SBS signature exposure

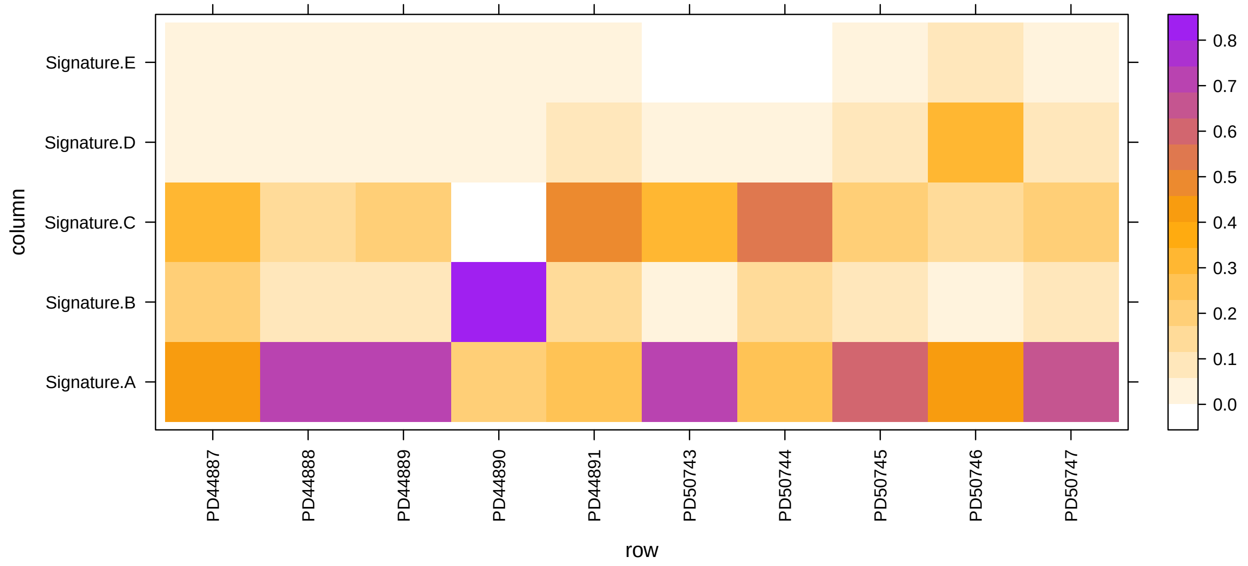

Supplementary Figure 26 - HDP vs SigProfiler cosine similarity matrix

Heatmap showing cosine similarity of HDP and SigProfiler signature components. HDP components plotted (x-axis) against SigProfiler components (y-axis).

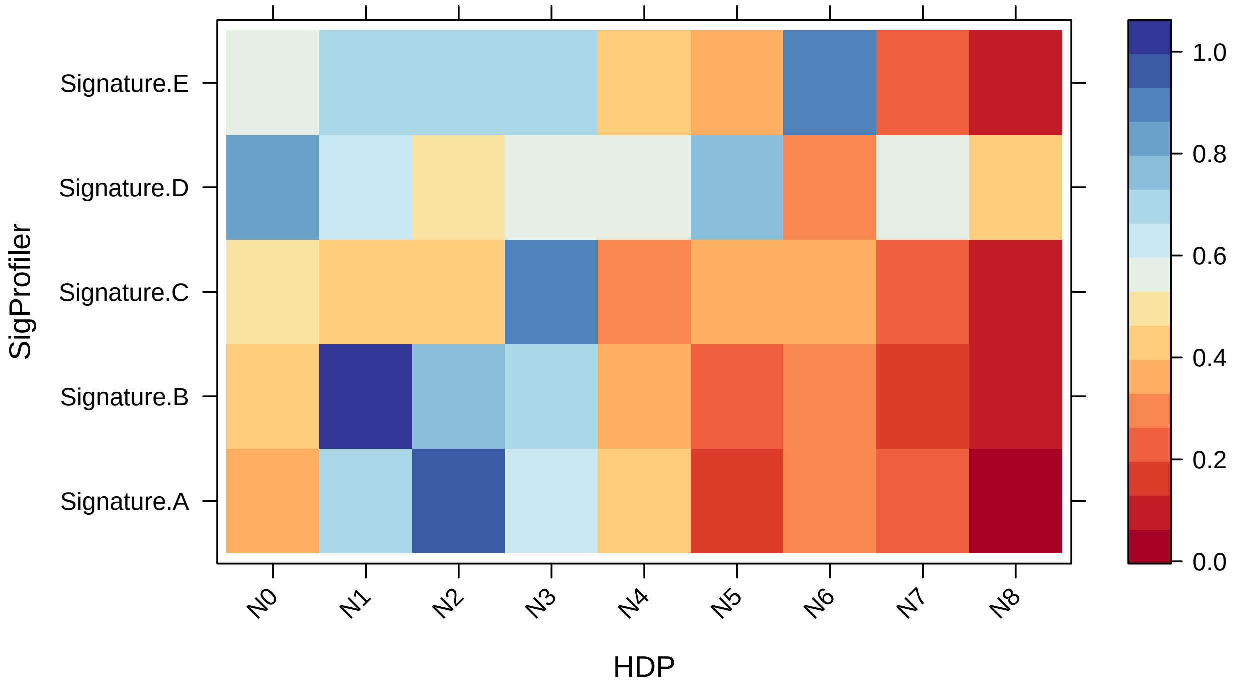

HDP ID Signature components

Plots show probability distribution of extracted components, trinucleotide context (x-axis) and proportion (y-axis). Credibility intervals (95%) are displayed for each bar which are calculated from the set of posterior samples (n=2500) collected during the algorithm's computation. Mutation contexts whose contribution is not statistically significant are shown as light grey bars.

Supplementary Figure 27 - ID HDP N0

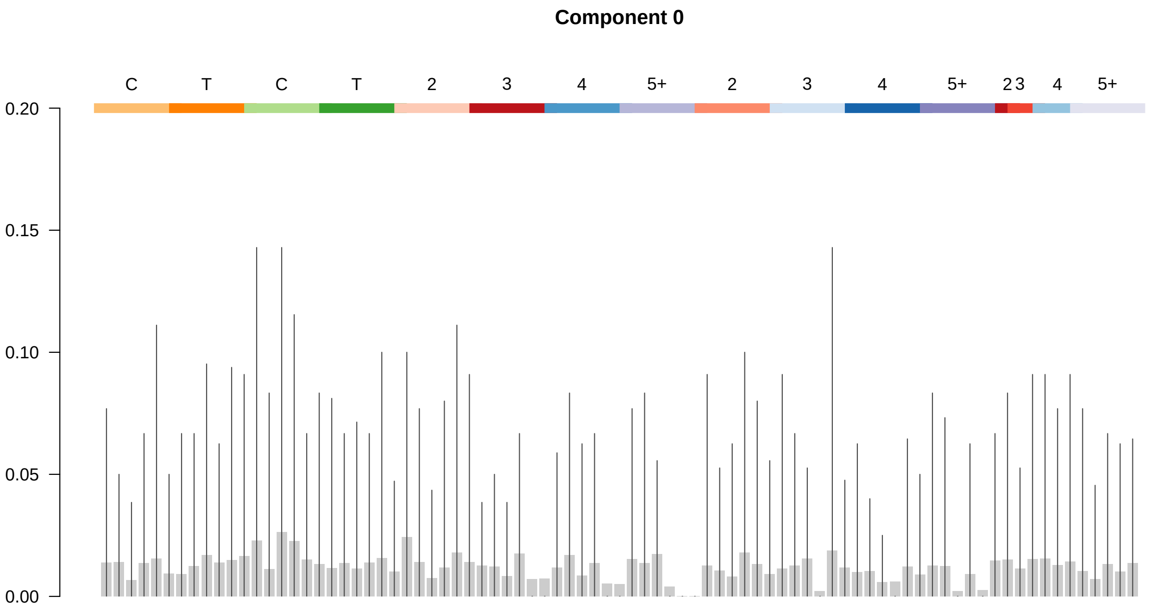

Supplementary Figure 28 - ID HDP N1

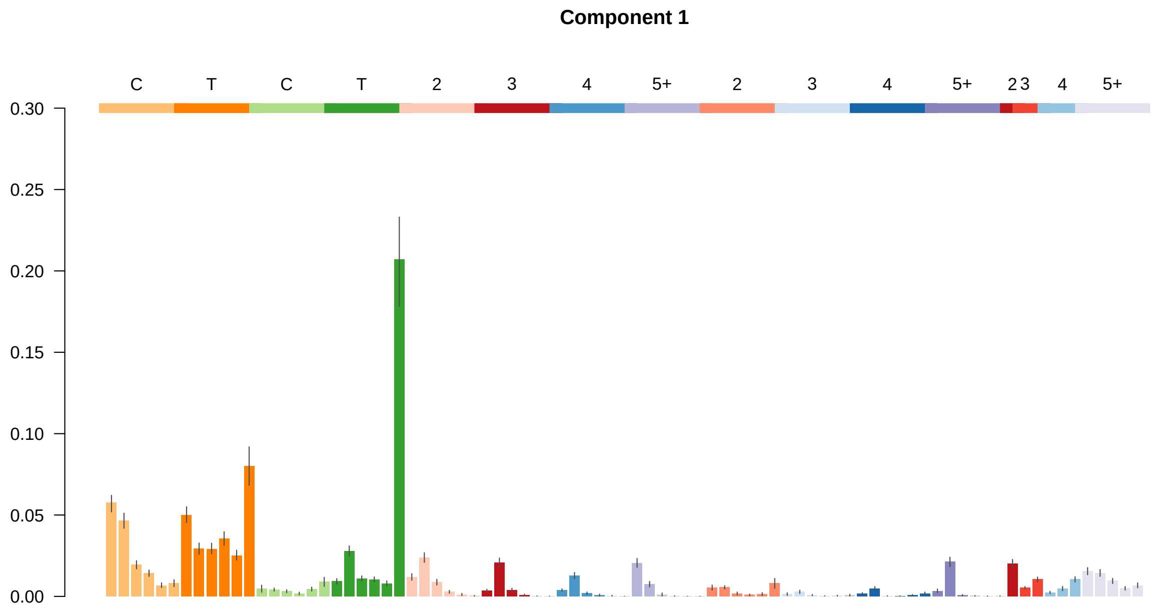

Supplementary Figure 29 - ID HDP N2

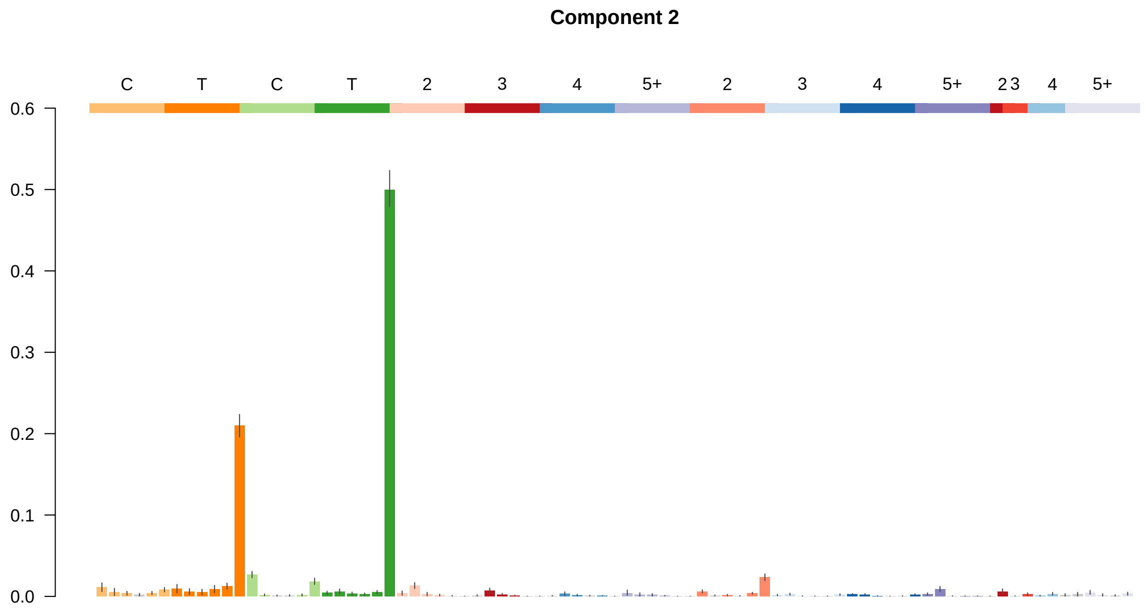

Supplementary Figure 30 - ID HDP N3

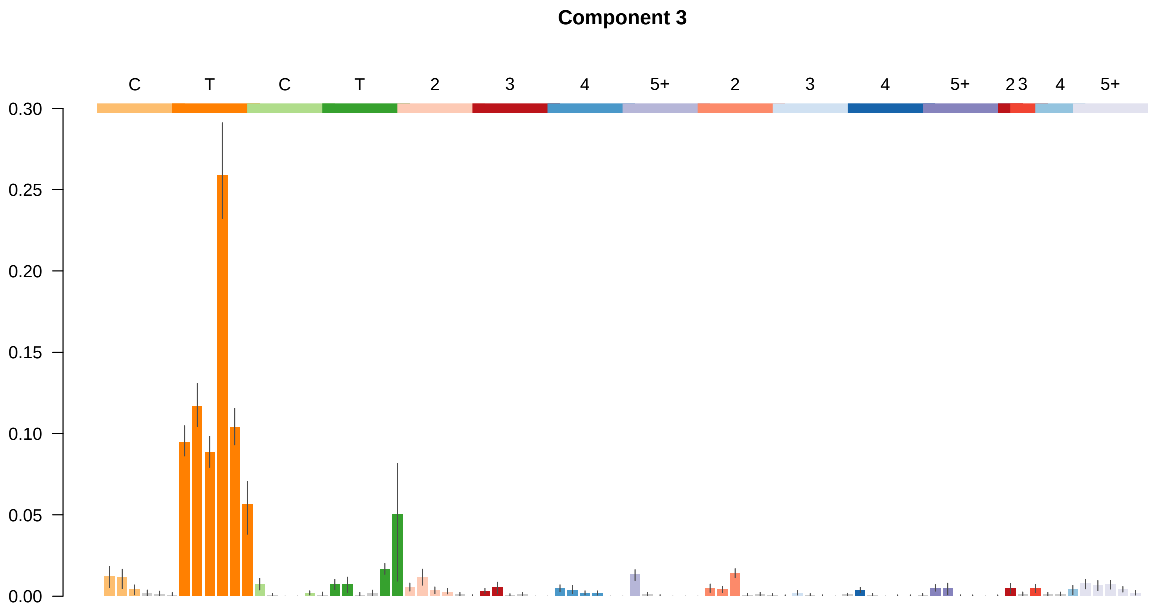

Supplementary Figure 31 - ID HDP N4

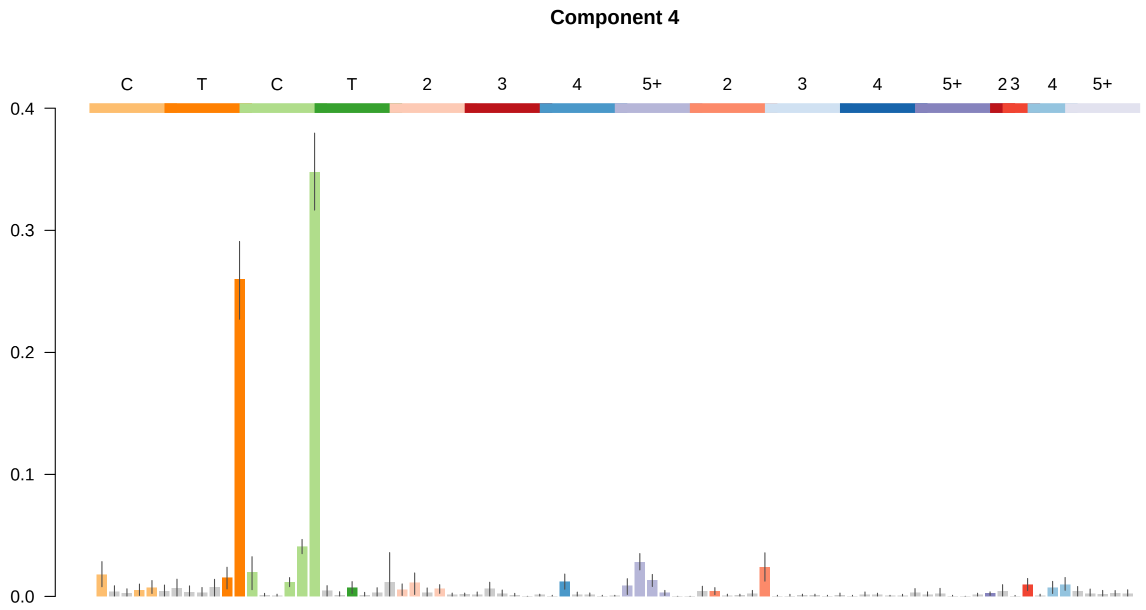

HDP ID Signature components - deconvolution

Five ID signature components were extracted. Two components bore close similarity to known mutational signatures. N1 is a good recapitulation of ID1 and N3 of ID18. These two components were therefore not deconvoluted. Signature deconvolution was performed on signature components N0,N2, and N4. Reference signatures from the PCAWG database were used for deconvolution (Methods).

Plots show mutational spectra of extracted component; mutational type / context (x-axis) and proportion (y-axis). Each HDP component is displayed in the uppermost panel followed by the reconstituted component after deconvolution and its cosine similarity to the original HDP component. PCAWG components and their relative contributions are displayed on the lower panels.

Supplementary Figure 32 - ID HDP N0

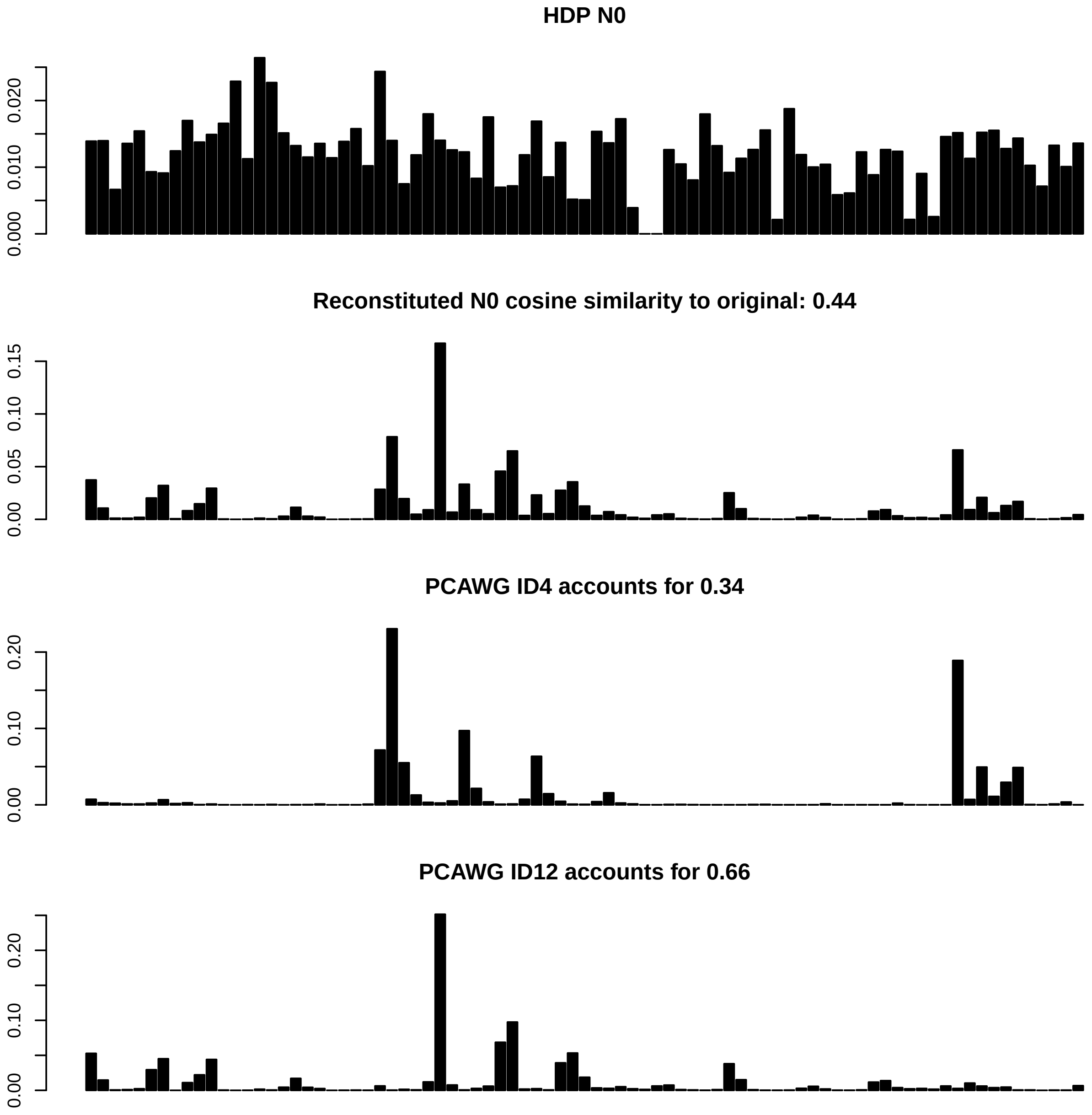

Supplementary Figure 33 - ID HDP N2

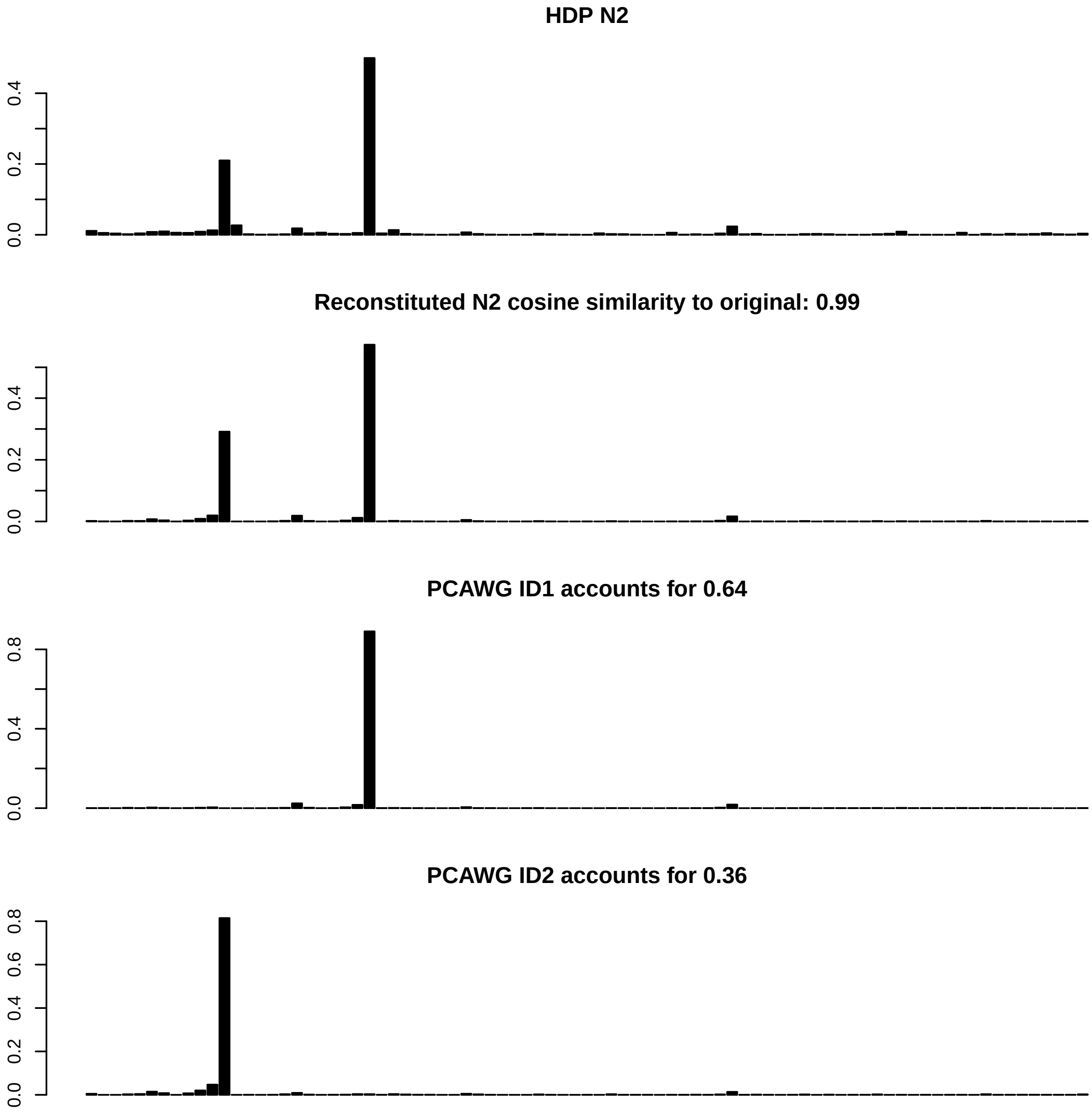

Supplementary Figure 34 - ID HDP N4

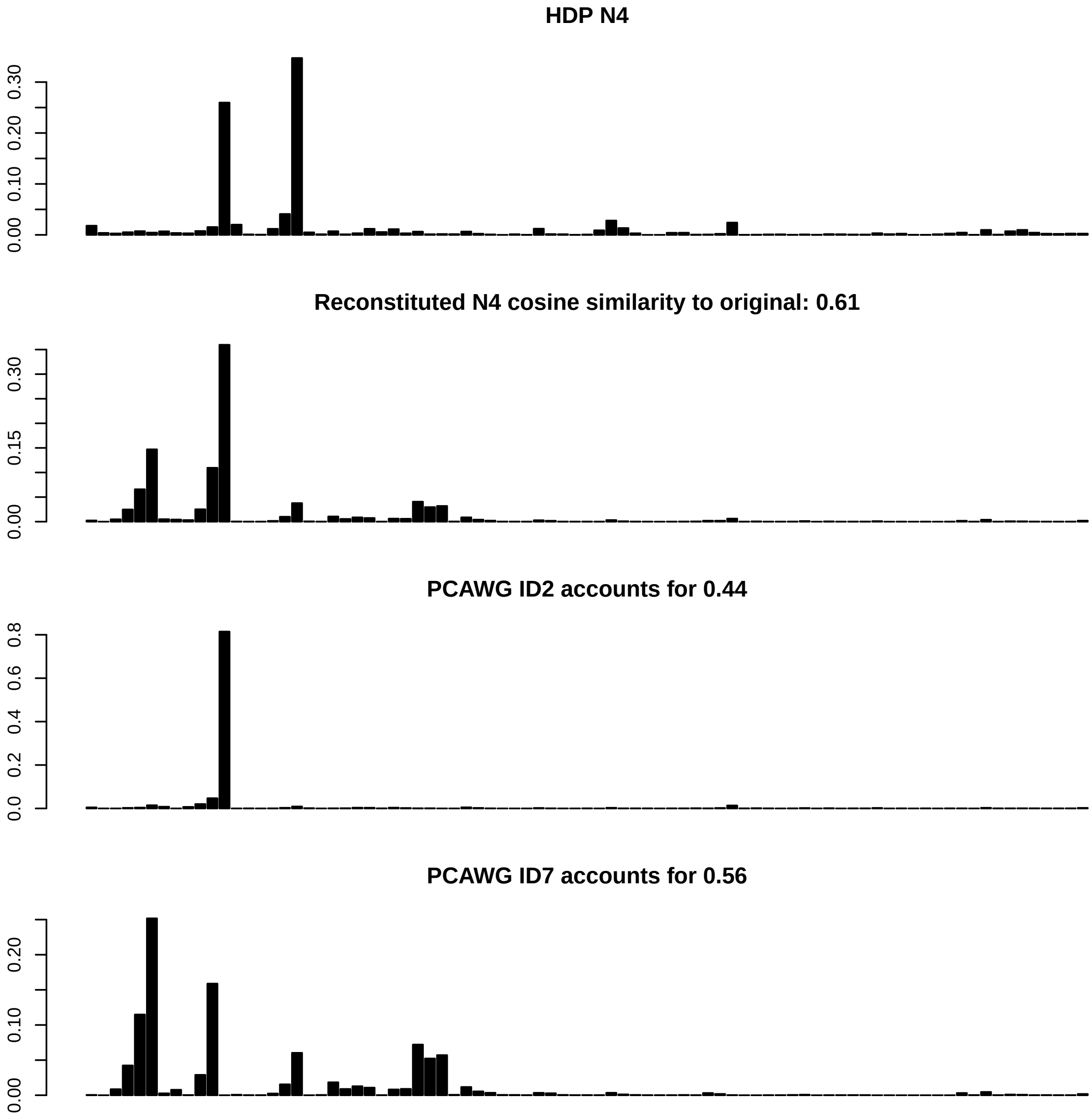

SigProfiler ID Signature components

Four ID signature components were identified via *de novo* extraction. Plots show mutational spectra of extracted components; mutational type / context (x-axis) and proportion (y-axis).

Supplementary Figure 35

ID SigProfiler A

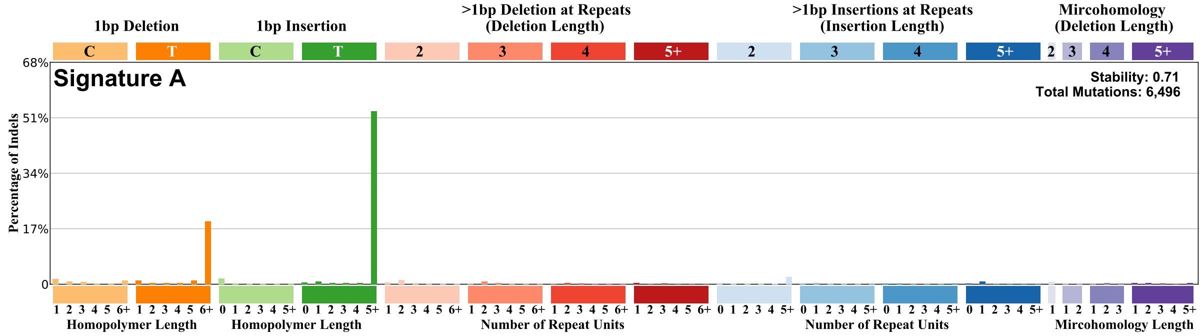

ID SigProfiler B

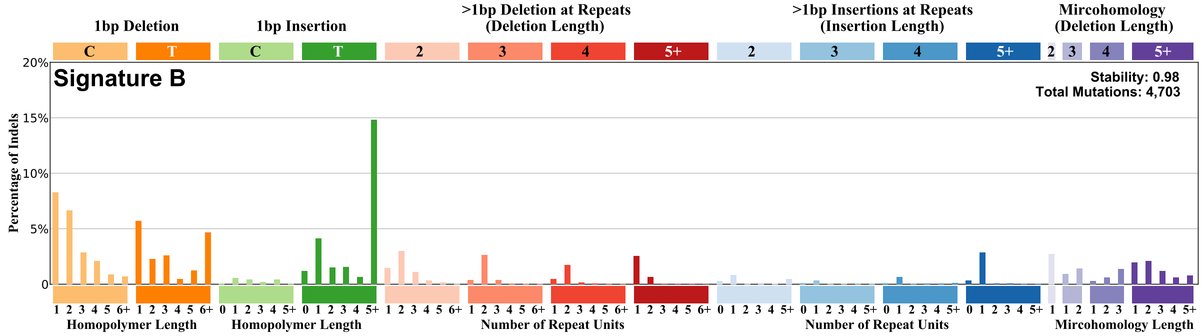

ID SigProfiler C

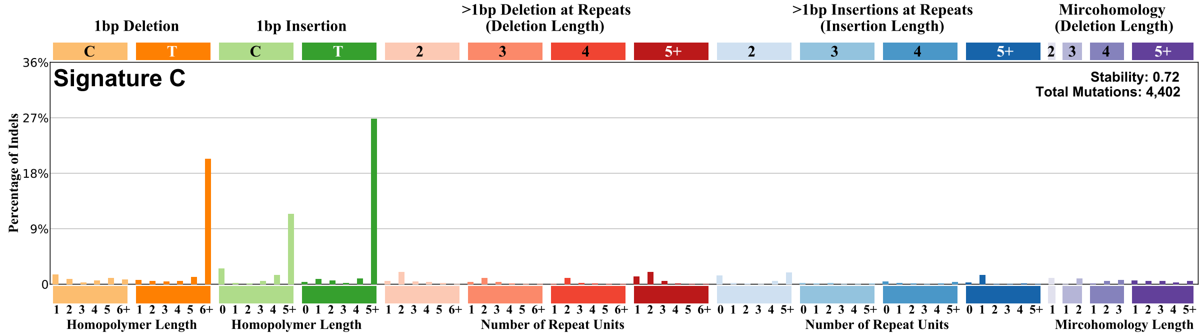

ID SigProfiler D

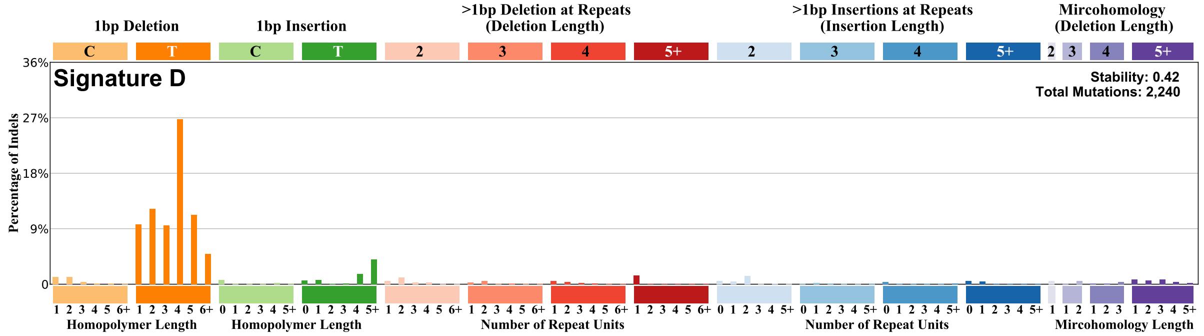

Supplementary Data - Legends

Supplementary Data 1

Supplementary Data 1 | Clinical summary and phenotypic characteristics of individuals with germline MUTYH mutations
Summary of phenotypic features and disease burden in all individuals in this cohort.

Supplementary Data 2

Supplementary Data 2 | Sample summary
Summary of samples sequenced including germline *MUTYH* mutation, sequencing method and mutation burdens

Supplementary Data 3

Supplementary Data 3 | Cancer driver mutations
Cancer driver mutations identified across all samples in this cohort

Supplementary Note 2 - Mutational signatures in PD44890

Individual PD44890 displayed a substantially elevated mutation burden (~33 fold increase) compared to healthy controls. This increased mutation burden is also many times greater than other individuals with *MUTYH mutations* included in this study. We identified two germline *OGG1* mutations which may potentially contribute to the mutation rate in this individual. In addition, the trinucleotide spectrum of mutations shows distinctive peaks, with overrepresentation of C>A mutations at GCA, ACA and to a lesser extent CCA (mutated base underlined). In a recent *in vitro* study, deletion of *OGG1* was shown to induce a distinctive mutational signature that shows some similarity to the spectra observed in samples from PD44890 (Zou et al 2021, see Supplementary Figure 1).

Signature components HDP N2 and SigProfiler A were abundant in PD44890 and were absent when this individual was removed from the signature extraction, suggesting that this component was heavily influenced by the mutations from this individual. Initial deconvolution of HDP N2 demonstrated contributions from reference signatures SBS18 and SBS36. The combination of these two signatures recapitulated most of the peaks seen in this individual (cosine similarity of original and reconstituted signature of 0.9). This degree of cosine similarity (>/=0.9) is a prospectively set threshold for acceptability of reconstruction and thus the absence of need to propose a new signature.

Nevertheless, since HDP N2 bore some similarity to the SBSOGG1 signature (Supplementary Figure 1), we were encouraged to further explore and quantify a potential relationship between the SBSOGG1 signature and our extracted signature component. To assess a potential contribution of the OGG1 mutational process, deconvolution of HDP N2 was repeated using SBS18, SBS36 and SBSOGG1. The cosine similarity was assessed, comparing the reconstituted component with the original signature component. The reconstituted signature component corresponding to HDP N2 showed improved cosine similarity metrics using SBSOGG1 when compared to deconvolution without SBSOGG1 (Table below). This would support the proposal that SBSOGG1 may contribute to the spectrum observed in samples from this individual.

Lastly, the combined spectra of SBS36 and SBSOGG1 also bore close similarity to the mutational spectra and signature components observed in several cell and tissue experiments ascribed to SBS18 which is thought to be due to the effect of *in vitro* or *in vivo* reactive oxygen species (ROS) related DNA damage. This may possibly suggest that SBS18 is a composite of the two mutational processes that underlie SBS36 and SBSOGG1.

Supplementary Table 1

The rows of this table indicate various permutations of reference signatures used in deconvolution. The columns show the cosine similarity of each combination to each reference signature SBS18, SBS36 and SBSOGG1 and finally the original HDP N2 signature component.

X	SBS18	SBS36	SBSOGG1	Cosine.sim..original.vs.reconstituted
SBS18 & SBS36	0.60	0.40	NA	0.90
SBS18 & SBS36 & SBSOGG1	0.00	0.52	0.48	0.98
SBS18 & SBSOGG1	0.52	NA	0.48	0.95
SBS36 & SBSOGG1	NA	0.52	0.48	0.98
