# Supplementary material for: Inherited MUTYH mutations cause elevated somatic mutation rates and distinctive mutational signatures in normal human cells
Source: Nat Commun. 2022 Jul 8;13:3949. doi: 10.1038/s41467-022-31341-0 (PMC9270427; doi:10.1038/s41467-022-31341-0)
Supplement: Supplementary file 5 — Reporting Summary [file 41467_2022_31341_MOESM5_ESM.pdf]

nature portfolio

Corresponding author(s): Michael R Stratton

Last updated by author(s): 04/02/2022

Reporting Summary

Nature Portfolio wishes to improve the reproducibility of the work that we publish. This form provides structure for consistency and transparency in reporting. For further information on Nature Portfolio policies, see our Editorial Policies and the Editorial Policy Checklist.

Statistics

For all statistical analyses, confirm that the following items are present in the figure legend, table legend, main text, or Methods section.

n/a	Confirmed	
☐	☒	The exact sample size (*n*) for each experimental group/condition, given as a discrete number and unit of measurement
☐	☒	A statement on whether measurements were taken from distinct samples or whether the same sample was measured repeatedly
☐	☒	The statistical test(s) used AND whether they are one- or two-sided<br>*Only common tests should be described solely by name; describe more complex techniques in the Methods section.*
☐	☒	A description of all covariates tested
☐	☒	A description of any assumptions or corrections, such as tests of normality and adjustment for multiple comparisons
☐	☒	A full description of the statistical parameters including central tendency (e.g. means) or other basic estimates (e.g. regression coefficient) AND variation (e.g. standard deviation) or associated estimates of uncertainty (e.g. confidence intervals)
☐	☒	For null hypothesis testing, the test statistic (e.g. *F*, *t*, *r*) with confidence intervals, effect sizes, degrees of freedom and *P* value noted<br>*Give P values as exact values whenever suitable.*
☒	☐	For Bayesian analysis, information on the choice of priors and Markov chain Monte Carlo settings
☒	☐	For hierarchical and complex designs, identification of the appropriate level for tests and full reporting of outcomes
☒	☐	Estimates of effect sizes (e.g. Cohen's *d*, Pearson's *r*), indicating how they were calculated

Our web collection on statistics for biologists contains articles on many of the points above.

Software and code

Policy information about availability of computer code

Data collection

No software was used in data collection

Data analysis

Somatic mutation calling was performed using the Wellcome Sanger Institute's mutation calling algorithms as part of a bespoke pipeline. Single Base Substitutions were called using CaVEMan (cancer variants through expectation maximization) algorithm (1.14.1 and 1.15.1). Small insertions and deletions (ID) were called using Pindel algorithm (3.3.0). Copy number alterations were called using ASCAT (4.3.2) Algorithms are publicly available through the Cancer IT / CASM GitHub page https://github.com/cancerit.

Rearrangements were called using GRIDSS algorithm (2.9.4). Available from https://github.com/PapenfussLab/gridss

Custom algorithms used in filtering somatic mutations are available from https://github.com/TimCoorens/Unmatched_NormSeq and additional scripts and data are available at https://github.com/PhilipSRobinson/mutyh DOI: DOI: 10.5281/zenodo.6504797

Histology images were viewed with NDP View 2 https://www.hamamatsu.com/jp/en/product/life-science-and-medical-systems/digital-slide-scanner/U12388-01.html

Mutational signature extraction was performed with the R package HDP https://github.com/nicolaroberts/hdp
Mutational signatures were fitted with the R package - SigFit https://github.com/kgori/sigfit

R packages:

ade4 1.7-15 CRAN
AnnotationDbi 1.48.0 Bioconductor

ape 5.4 CRAN
backports 1.1.8 CRAN
base64enc 0.1-3 CRAN
bayesplot 1.7.2 CRAN
BH 1.72.0-3 CRAN
Biobase 2.46.0 Bioconductor
BiocFileCache 1.10.2 Bioconductor
BiocGenerics 0.32.0 Bioconductor
BiocParallel 1.20.1 Bioconductor
BiocStyle 2.14.4 Bioconductor
biomaRt 2.42.1 Bioconductor
Biostrings 2.54.0 Bioconductor
bit 4.0.4 CRAN
bit64 4.0.2 CRAN
bitops 1.0-6 CRAN
blob 1.2.1 CRAN
bookdown 0.2 CRAN
broom 0.7.0 CRAN
BSgenome 1.54.0 Bioconductor
BSgenome.Hsapiens.1000genomes.hs37d5 0.99.1 Bioconductor
BSgenome.Hsapiens.UCSC.hg19 1.4.0 Bioconductor
cellranger 1.1.0 CRAN
checkmate 2.0.0 CRAN
clue 0.3-57 CRAN
clusterGeneration 1.3.5 CRAN
coda 0.19-3 CRAN
colourpicker 1.1.0 CRAN
combinat 0.0-8 CRAN
corpcor 1.6.9 CRAN
cpp11 0.2.1 CRAN
cubature 2.0.4.1 CRAN
data.table 1.13.0 CRAN
DBI 1.1.0 CRAN
dbplyr 1.4.4 CRAN
deconstructSigs 1.8.0 CRAN
DelayedArray 0.12.3 Bioconductor
digest 0.6.25 CRAN
dndscv 0.0.1.0 GitHub
dplyr 1.0.1 CRAN
dygraphs 1.1.1.6 CRAN
ellipsis 0.3.1 CRAN
expm 0.999-5 CRAN
fansi 0.4.1 CRAN
farver 2.0.3 CRAN
fastmatch 1.1-0 CRAN
flexclust 1.4-0 CRAN
forcats 0.5.0 CRAN
formatR 1.7 CRAN
futile.logger 1.4.3 CRAN
futile.options 1.0.1 CRAN
generics 0.0.2 CRAN
GenomeInfoDb 1.22.1 Bioconductor
GenomeInfoDbData 1.2.2 Bioconductor
GenomicAlignments 1.22.1 Bioconductor
GenomicFeatures 1.38.2 Bioconductor
GenomicRanges 1.38.0 Bioconductor
ggplot2 3.3.2 CRAN
ggridges 0.5.2 CRAN
ggtree 2.0.4 Bioconductor
glue 1.4.1 CRAN
gridExtra 2.3 CRAN
gtools 3.8.2 CRAN
haven 2.3.1 CRAN
hdp 0.1.5 GitHub
highr 0.8 CRAN
hms 0.5.3 CRAN
igraph 1.2.5 CRAN
inline 0.3.15 CRAN
IRanges 2.20.2 Bioconductor
isoband 0.2.2 CRAN
jsonlite 1.7.0 CRAN
knitr 1.29 CRAN
lambda.r 1.2.4 CRAN
lifecycle 0.2.0 CRAN
lme4 1.1-25 CRAN
loo 2.3.1 CRAN

lsa 0.73.2 CRAN
lubridate 1.7.9 CRAN
maps 3.3.0 CRAN
markdown 1.1 CRAN
matrixStats 0.56.0 CRAN
MCMCglmm 2.32 CRAN
mime 0.9 CRAN
miniUI 0.1.1.1 CRAN
minqa 1.2.4 CRAN
mnormt 2.0.2 CRAN
modelr 0.1.8 CRAN
modeltools 0.2-23 CRAN
MuMIn 1.43.17 CRAN
nloptr 1.2.2.2 CRAN
numDeriv 2016.8-1.1 CRAN
packrat 0.5.0 CRAN
phangorn 2.5.5 CRAN
phytools 0.7-70 CRAN
pixmap 0.4-11 CRAN
plogr 0.2.0 CRAN
plotrix 3.7-8 CRAN
poilog 0.4 CRAN
prettyunits 1.1.1 CRAN
progress 1.2.2 CRAN
ps 1.3.4 CRAN
quadprog 1.5-8 CRAN
rappdirs 0.3.1 CRAN
RColorBrewer 1.1-2 CRAN
Rcpp 1.0.5 CRAN
RcppEigen 0.3.3.7.0 CRAN
RcppParallel 5.0.2 CRAN
RCurl 1.98-1.2 CRAN
readr 1.3.1 CRAN
readxl 1.3.1 CRAN
rematch 1.0.1 CRAN
rematch2 2.1.2 CRAN
reprex 0.3.0 CRAN
reticulate 1.16 CRAN
Rhtslib 1.18.1 Bioconductor
rlang 0.4.7 CRAN
rlist 0.4.6.1 CRAN
rmarkdown 2.3 CRAN
Rsamtools 2.2.3 Bioconductor
rsconnect 0.8.16 CRAN
RSQLite 2.2.0 CRAN
rstan 2.21.2 CRAN
rstantools 2.1.1.9000 CRAN
rstudioapi 0.11 CRAN
rtracklayer 1.46.0 Bioconductor
RUnit 0.4.32 CRAN
rvcheck 0.1.8 CRAN
rvest 0.3.6 CRAN
S4Vectors 0.24.4 Bioconductor
scatterplot3d 0.3-41 CRAN
segmented 1.2-0 CRAN
selectr 0.4-2 CRAN
seqinr 3.6-1 CRAN
shinyjs 2.0.0 CRAN
shinythemes 1.1.2 CRAN
sigfit 2.0.0 GitHub
SigProfilerMatrixGeneratorR 0.1.0 GitHub
snow 0.4-3 CRAN
SnowballC 0.7.0 CRAN
sp 1.4-2 CRAN
StanHeaders 2.21.0-6 CRAN
statmod 1.4.35 CRAN
stringi 1.4.6 CRAN
SummarizedExperiment 1.16.1 Bioconductor
tensorA 0.36.2 CRAN
threejs 0.3.3 CRAN
tibble 3.0.3 CRAN
tidyr 1.1.1 CRAN
tidyselect 1.1.0 CRAN
tidytree 0.3.3 CRAN
tidyverse 1.3.0 CRAN
tinytex 0.25 CRAN

tmvnsim 1.0-2 CRAN
treeio 1.10.0 Bioconductor
V8 3.3.1 CRAN
VariantAnnotation 1.32.0 Bioconductor
vctrs 0.3.2 CRAN
VGAM 1.1-3 CRAN
viridis 0.5.1 CRAN
withr 2.2.0 CRAN
writexl 1.3.1 CRAN
xfun 0.16 CRAN
XML 3.99-0 CRAN
xts 0.12.1 CRAN
XVector 0.26.0 Bioconductor
yaml 2.2.1 CRAN
zoo 1.8-8 CRAN
askpass 1.1 CRAN
assertthat 0.2.1 CRAN
backports 1.1.5 CRAN
base64enc 0.1-3 CRAN
BH 1.72.0-2 CRAN
BiocManager 1.30.10 CRAN
BiocVersion 3.10.1 Bioconductor
brew 1.0-6 CRAN
callr 3.4.0 CRAN
cli 2.0.0 CRAN
clipr 0.7.0 CRAN
clisymbols 1.2.0 CRAN
colorspace 1.4-1 CRAN
commonmark 1.7 CRAN
covr 3.4.0 CRAN
crayon 1.3.4 CRAN
crosstalk 1.0.0 CRAN
curl 4.3 CRAN
desc 1.2.0 CRAN
devtools 2.2.1 CRAN
digest 0.6.23 CRAN
DT 0.11 CRAN
ellipsis 0.3.0 CRAN
evaluate 0.14 CRAN
fansi 0.4.0 CRAN
farver 2.0.1 CRAN
fastmap 1.0.1 CRAN
fs 1.3.1 CRAN
ggplot2 3.2.1 CRAN
gh 1.0.1 CRAN
git2r 0.26.1 CRAN
glue 1.3.1 CRAN
gtable 0.3.0 CRAN
htmltools 0.4.0 CRAN
htmlwidgets 1.5.1 CRAN
httpuv 1.5.2 CRAN
httr 1.4.1 CRAN
ini 0.3.1 CRAN
IRdisplay 1 CRAN
IRkernel 1.2 CRAN
jsonlite 1.6 CRAN
labeling 0.3 CRAN
later 1.0.0 CRAN
lazyeval 0.2.2 CRAN
lifecycle 0.1.0 CRAN
magrittr 1.5 CRAN
memoise 1.1.0 CRAN
mime 0.8 CRAN
munsell 0.5.0 CRAN
openssl 1.4.1 CRAN
pbdZMQ 0.3-5 CRAN
pillar 1.4.3 CRAN
pkgbuild 1.0.6 CRAN
pkgconfig 2.0.3 CRAN
pkgload 1.0.2 CRAN
plyr 1.8.5 CRAN
praise 1.0.0 CRAN
prettyunits 1.0.2 CRAN
processx 3.4.1 CRAN
promises 1.1.0 CRAN
ps 1.3.0 CRAN

purrr 0.3.3 CRAN
R6 2.4.1 CRAN
rcmdcheck 1.3.3 CRAN
RColorBrewer 1.1-2 CRAN
Rcpp 1.0.3 CRAN
remotes 2.1.0 CRAN
repr 1.1.3 CRAN
reshape2 1.4.3 CRAN
rex 1.1.2 CRAN
rlang 0.4.2 CRAN
roxygen2 7.0.2 CRAN
rprojroot 1.3-2 CRAN
rstudioapi 0.1 CRAN
rversions 2.0.1 CRAN
scales 1.1.0 CRAN
sessioninfo 1.1.1 CRAN
shiny 1.4.0 CRAN
sourcetools 0.1.7 CRAN
stringi 1.4.3 CRAN
stringr 1.4.0 CRAN
sys 3.3 CRAN
testthat 2.3.1 CRAN
tibble 2.1.3 CRAN
usethis 1.5.1 CRAN
utf8 1.1.4 CRAN
uuid 0.1-4 CRAN
vctrs 0.2.1 CRAN
viridisLite 0.3.0 CRAN
whisker 0.4 CRAN
withr 2.1.2 CRAN
xml2 1.2.2 CRAN
xopen 1.0.0 CRAN
xtable 1.8-4 CRAN
yaml 2.2.0 CRAN
zeallot 0.1.0 CRAN
zlibbioc 1.32.0 Bioconductor

Other software:
Telomerehunter 1.1.0 (2015) PIP in a Python 2.7 virtual environment

SigProfiler was installed in a conda3 environment
The following versions of SigProfiler dependencies were installed:
Python Version: 3.7.4
Sigproextractor Version: 0.0.5.77
SigprofilerPlotting Version: 1.1.8
SigprofilerMatrixGenerator Version: 1.1.22
Pandas version: 1.1.0
Numpy version: 1.19.1
Scipy version: 1.5.2
Scikit-learn version: 0.23.1
Nimfa version: 1.4.0

For manuscripts utilizing custom algorithms or software that are central to the research but not yet described in published literature, software must be made available to editors and reviewers. We strongly encourage code deposition in a community repository (e.g. GitHub). See the Nature Portfolio guidelines for submitting code & software for further information.

Data

Policy information about availability of data

All manuscripts must include a data availability statement. This statement should provide the following information, where applicable:

- Accession codes, unique identifiers, or web links for publicly available datasets
- A description of any restrictions on data availability
- For clinical datasets or third party data, please ensure that the statement adheres to our policy

DNA sequencing data are deposited in the European Genome-Phenome Archive (EGA) with accession code: EGAD00001007958 and EGAD00001007997. The data are available via managed access.

The cBioPortal MutationMapper database was accessed at: https://www.cbioportal.org/mutation_mapper?standaloneMutationMapperGeneTab=ATM

Source data and code required to reproduce the figures in this manuscript are available at: https://github.com/PhilipSRobinson/mutyh DOI: 10.5281/zenodo.6504797

Field-specific reporting

Please select the one below that is the best fit for your research. If you are not sure, read the appropriate sections before making your selection.

☒ Life sciences ☐ Behavioural & social sciences ☐ Ecological, evolutionary & environmental sciences

For a reference copy of the document with all sections, see nature.com/documents/nr-reporting-summary-flat.pdf

Life sciences study design

All studies must disclose on these points even when the disclosure is negative.

Sample size	No formal sample size calculations were performed. A limited number of cases /individuals were available given the rarity of the germline mutation. A diverse selection of individuals carrying mutations across the two main DNA polymerase genes were selected.
Data exclusions	Five glands (PD50745b_lo0009, PD50745c_lo0001, PD50745c_lo0002, PD50745c_lo0006, PD50746b_lo0013) were excluded from analysis of SBS mutation burden due to their appearance being potentially consistent with being Brunner's glands rather than intestinal crypts. Sequencing data from these glands showed a very low mutation burden and mutational signature profile consistent with previous reports of small intestine Brunner's glands (Moore et al 2020). One adenoma sample (PD50746a) had atypical features consistent with a mucinous neoplasm and hence was excluded from analysis of adenomas.
Replication	This study and its experiments were designed to ensure that each germline mutation / gene studied was represented by multiple samples per patient and where possible multiple patients per affected germline genotype. Validation of the DNA isolation, library preparation and sequencing of low-DNA input samples has been undertaken for intestinal crypts as part of the studies published in Ellis et al 2020, Lee Six et al 2019. In these experiments biological replicates were obtained for individuals crypts (serial sections from n=17 crypts). These replicates were independently isolated and processed and demonstrated good concordance. All replicates were successful. Further detail of the low-input method are detailed in Ellis et al Nature protocols 2020.<br>Replication of the modified duplex sequencing protocol used to sequence blood and sperm were performed as part of its development and are detailed in its manuscript (Abascal et al 2021).
Randomization	Not applicable - this study did not involve an intervention and as such no randomization was undertaken. Covariates such as age, germline MUTYH mutation and sequencing parameters were controlled for with statistical modeling where relevant.
Blinding	Not applicable - this study did not involve the allocation to groups and hence blinding was not performed.

Reporting for specific materials, systems and methods

We require information from authors about some types of materials, experimental systems and methods used in many studies. Here, indicate whether each material, system or method listed is relevant to your study. If you are not sure if a list item applies to your research, read the appropriate section before selecting a response.

Materials & experimental systems

n/a	Involved in the study	
☒	☐	Antibodies
☒	☐	Eukaryotic cell lines
☒	☐	Palaeontology and archaeology
☒	☐	Animals and other organisms
☐	☒	Human research participants
☒	☐	Clinical data
☒	☐	Dual use research of concern

Methods

n/a	Involved in the study	
☒	☐	ChIP-seq
☒	☐	Flow cytometry
☒	☐	MRI-based neuroimaging

Human research participants

Policy information about studies involving human research participants

Population characteristics	This study involves analysis of samples from individuals aged 16-79 years (male n=4, female n=6) with germline MUTYH mutations. Samples from normal healthy controls aged 19-80 years were also included for comparison. Analysis of other conditions and treatments was not performed.
Recruitment	Individuals were recruited in the studies outlined below under informed consent according to study protocols. In brief, individuals with clinically suspected FAP or MAP were recruited to the study under informed consent. Genetic testing was then undertaken to confirm the diagnosis of MAP. No obvious bias was introduced via the recruitment process. The recruitment process is therefore very unlikely to have impacted the results.

Intestinal mucosal biopsies were collected during routine endoscopy. Additional biopsies were taken for the purpose of research as per the protocol as outlined in the REC documentation.

Peripheral blood was collected and stored under informed consent according to the protocols outlined in the REC documentation.

Ethics oversight	MAP patients were recruited as part of Wales Research Ethics Committee (REC) 12-WA0071 and 15-WA0075 and samples collected were approved for use in this specific project by REC 18/ES/0133. Normal healthy controls were recruited as part of the following UK Research Ethics Committee (REC) studies; 15/WA/0131, 15/EE/0152, 18/ES/0133 and 08/h0304/85+5.

Note that full information on the approval of the study protocol must also be provided in the manuscript.
